# Cerebral physiologic insult burden in acute traumatic neural injury: a Canadian High Resolution-TBI (CAHR-TBI) descriptive analysis

**DOI:** 10.1186/s13054-024-05083-y

**Published:** 2024-09-04

**Authors:** Kevin Y. Stein, Alwyn Gomez, Donald Griesdale, Mypinder Sekhon, Francis Bernard, Clare Gallagher, Eric P. Thelin, Rahul Raj, Marcel Aries, Logan Froese, Andreas Kramer, Frederick A. Zeiler

**Affiliations:** 1 Department of Biomedical Engineering, Price Faculty of Engineering, University of Manitoba, Winnipeg, MB Canada; 2 Max Rady College of Medicine, Rady Faculty of Health Sciences, University of Manitoba, Winnipeg, MB Canada; 3 Department of Human Anatomy and Cell Science, Rady Faculty of Health Sciences, University of Manitoba, Winnipeg, MB Canada; 4 Section of Neurosurgery, Department of Surgery, Rady Faculty of Health Sciences, University of Manitoba, Winnipeg, MB Canada; 5 Department of Anesthesiology, Pharmacology, and Therapeutics, University of British Columbia, Vancouver, BC Canada; 6 Division of Critical Care, Department of Medicine, University of British Columbia, Vancouver, BC Canada; 7 Section of Critical Care, Department of Medicine, University of Montreal, Montreal, QC Canada; 8 Section of Neurosurgery, University of Calgary, Calgary, AB Canada; 9 Department of Clinical Neurosciences, University of Calgary, Calgary, AB Canada; 10 Hotchkiss Brain Institute, University of Calgary, Calgary, AB Canada; 11 Medical Unit Neurology, Karolinska University Hospital, Stockholm, Sweden; 12 Department of Clinical Neuroscience, Karolinska Institutet, Stockholm, Sweden; 13 Department of Neurosurgery, University of Helsinki and Helsinki University Hospital, Helsinki, Finland; 14 Department of Intensive Care, Maastricht University Medical Center+ and School of Mental Health and Neurosciences, University Maastricht, Maastricht, The Netherlands; 15 Department of Critical Care Medicine, University of Calgary, Calgary, AB Canada; 16 Pan Am Clinic Foundation, Winnipeg, MB Canada

**Keywords:** Traumatic brain injury, Cerebral physiologic insult burden, Multi-modal monitoring, Cerebral physiology

## Abstract

**Background:**

Over the recent decades, continuous multi-modal monitoring of cerebral physiology has gained increasing interest for its potential to help minimize secondary brain injury following moderate-to-severe acute traumatic neural injury (also termed traumatic brain injury; TBI). Despite this heightened interest, there has yet to be a comprehensive evaluation of the effects of derangements in multimodal cerebral physiology on global cerebral physiologic insult burden. In this study, we offer a multi-center descriptive analysis of the associations between deranged cerebral physiology and cerebral physiologic insult burden.

**Methods:**

Using data from the Canadian High-Resolution TBI (CAHR-TBI) Research Collaborative, a total of 369 complete patient datasets were acquired for the purposes of this study. For various cerebral physiologic metrics, patients were trichotomized into low, intermediate, and high cohorts based on mean values. Jonckheere–Terpstra testing was then used to assess for directional relationships between these cerebral physiologic metrics and various measures of cerebral physiologic insult burden. Contour plots were then created to illustrate the impact of preserved vs impaired cerebrovascular reactivity on these relationships.

**Results:**

It was found that elevated intracranial pressure (ICP) was associated with more time spent with cerebral perfusion pressure (CPP) < 60 mmHg and more time with impaired cerebrovascular reactivity. Low CPP was associated with more time spent with ICP > 20 or 22 mmHg and more time spent with impaired cerebrovascular reactivity. Elevated cerebrovascular reactivity indices were associated with more time spent with CPP < 60 mmHg as well as ICP > 20 or 22 mmHg. Low brain tissue oxygenation (PbtO_2_) only demonstrated a significant association with more time spent with CPP < 60 mmHg. Low regional oxygen saturation (rSO_2_) failed to produce a statistically significant association with any particular measure of cerebral physiologic insult burden.

**Conclusions:**

Mean ICP, CPP and, cerebrovascular reactivity values demonstrate statistically significant associations with global cerebral physiologic insult burden; however, it is uncertain whether measures of oxygen delivery provide any significant insight into such insult burden.

**Supplementary Information:**

The online version contains supplementary material available at 10.1186/s13054-024-05083-y.

## Background

The pathophysiology underlying traumatic brain injury (TBI) can generally be divided into two distinct phases: primary and secondary brain injury. Primary injury refers to the immediate disruptions in brain structure, such as contusions, hemorrhages, or diffuse axonal injuries, that occur as a direct result of the mechanical forces involved in the traumatic incident [1, 2]. Secondary brain injury encompasses the downstream systemic and cellular derangements, driven mainly by host responses to the primary injury, which result in ongoing neuronal death and delayed recovery [2, 3]. Since little can be done to reverse primary brain injury, moderate-to-severe TBI management is primarily focused on limiting secondary injury.

Intracranial hypertension and cerebral ischemia are two significant contributors to secondary brain injury and have been found to be strongly associated with poor outcomes [4–9]. Therefore, intracranial pressure (ICP) and cerebral perfusion pressure (CPP) have become key targets for therapeutic intervention in the management of moderate-to-severe TBI. Invasive ICP and arterial blood pressure (ABP) monitoring allow for the continuous monitoring of these parameters, CPP = ABP–ICP, and guide clinical management in this patient cohort. Current established management guidelines recommend maintaining ICP below a threshold of 20 or 22 mmHg and CPP between 60 and 70 mmHg [10, 11]. However, despite significant advances in our ability to achieve these targets over the past few decades, there has been limited improvement in the outcomes associated with moderate-to-severe TBI [2, 12, 13].

As a result, there has been increasing interest in the integration of additional continuous cerebral physiologic monitoring modalities to help further minimize secondary injury following TBI and improve outcomes. Due to their high metabolic demand and limited energy stores, cerebral tissues are highly sensitive to ischemia, with a mere five minutes of obstructed blood flow being able to result in irreversible damage [14]. Therefore, a handful of monitoring modalities that provide insight into the adequacy of cerebral perfusion and nutrient delivery have been considered for their potential to augment current clinical management. These include cerebrovascular reactivity, brain tissue oxygenation (PbtO_2_), and near-infrared spectroscopy (NIRS) monitoring.

Despite the increasing interest in multimodal cerebral physiologic monitoring, there is yet to be a comprehensive evaluation of the effects of derangements in continuous multimodal cerebral physiologic parameters on measures of cerebral physiologic insult burden. We, therefore, offer a multicenter descriptive analysis of the associations between derangements in continuous multimodal cerebral physiology and cerebral physiologic insult burden. Additionally, as a secondary aim, we hope to illustrate the protective effects that preserved cerebrovascular reactivity has on such relationships.

## Methods

### Study design

For the purposes of this retrospective descriptive analysis, we accessed prospectively collected datasets from the Canadian High Resolution-TBI (CAHR-TBI) Research Collaborative [15]. As part of this ongoing multicenter research collaborative, high-resolution physiologic data was collected from all adult (≥ 18 years) moderate-to-severe TBI patients admitted to the intensive care unit (ICU), for invasive physiologic monitoring, at one of four university-affiliated hospitals: Foothills Medical Centre (University of Calgary), Health Sciences Centre Winnipeg (University of Manitoba), Maastricht University Medical Center + (University of Maastricht), and Vancouver General Hospital (University of British Columbia).

Patients were included in this database if all of the following were satisfied: patient was admitted to the ICU of one of the member hospitals, patient was deemed to have suffered a moderate-to-severe TBI (as defined as having a Glasgow Coma Scale [GCS] of less than 13), patient had both invasive ICP and ABP monitoring available, patient was at least 18 years of age, initiation of data collection was possible within 24 h of the initial traumatic incident. Patients were excluded if any of these conditions were not satisfied, or if the patient had a cerebral shunt in place (as this would prevent observation of a proper ICP waveform).

In addition to high-resolution physiology data, demographics information (i.e. age, sex), admission characteristics (i.e. admission GCS, pupillary reactivity), imaging results (i.e. Marshall Computed Tomography score), and clinical outcome scoring (Glasgow Outcome Scale [GOS]) were also collected. All data collection occurred in an entirely de-identified fashion. Data entry spanned from 2011 to 2021 for the University of Calgary, 2019–2023 for the University of Manitoba, 2017–2022 for the University of Maastricht, and 2014–2019 for the University of British Columbia.

### Ethics

Ethics approval in regard to all aspects of data collection and anonymous data transfer between centers for this database has been granted by each site’s local research ethics board: University of Manitoba Biomedical Research Ethics Board (BREB, H2017:181, H2017:188, B2018:103, B2019:065, H2020:118, B2023:001), University of Calgary Conjoint Health Research Ethics Board (CHREB, H20-03759), University of British Columbia Clinical Research Ethics Board (CREB, REB20-0482) and University of Maastricht Medical Ethics Committee (16-4-243). As data collection is entirely anonymized, the respective research ethics boards have granted approval for data collection to operate under a waived consent model.

### Patient population

For our analysis, we retrospectively accessed all available archived datasets from the CAHR-TBI Research Collaborative. All patients had suffered a moderate-to-severe TBI, defined as having an admission Glasgow Coma Scale (GCS) of less than 13. Patients received standard care in accordance with Brain Trauma Foundation (BTF) guidelines, which recommends invasive monitoring of ICP and ABP, as well as therapeutic maintenance of ICP below 20–22 mmHg and CPP above 60 mmHg [10, 11]. It should be noted that hyperemic CPP was not typically treated as per local practice. Additionally, neither cerebrovascular reactivity monitoring nor NIRS-based rSO_2_ are part of any existing institutional clinical algorithms at any of the participating centers. However, it should be noted that these values are visible on patient bedside data collection carts and, therefore, could theoretically have been considered by treating clinicians during their clinical decision making.

At all participating hospitals, no established guideline on when PbtO_2_ monitoring should be utilized exists. Therefore, insertion of a Licox probe for monitoring PbtO_2_ is entirely at the discretion of the treating physician. Further, there is no established consensus among the participating centers on how PbtO_2_ should be used in guiding management, with local practice norms varying drastically between centers, from aggressive management to solely observational. However, the Brain Trauma Foundation guidelines do suggest maintenance of PbtO_2_ above 20 mmHg [11]. Physiologic data recordings were initiated within 24 h from the time of injury and following placement of invasive monitoring devices and admission into the ICU. Data collection continued until invasive physiologic monitoring was discontinued by the treating physician. At 6-month follow-up appointments, patients had their long-term outcomes evaluated and recorded using GOS.

### Physiologic data collection

ICP was continuously monitored using an intraparenchymal strain gauge probe (Codman ICP MicroSensor, Codman & Shurtlef Inc., Raynham, MA, USA; NEUROVENT-TEMP, RAUMEDIC, Helmbrechts, Germany) placed in the patient’s frontal lobe, or an external ventricular drain (EVD; Medtronic, Minneapolis, MN). No corrections were made for any zero drift of monitors. Continuous monitoring of ABP was performed using a radial or femoral line connected to a pressure transducer (Baxter Healthcare Corp. CardioVascular Group, Irvine, CA, USA; Edwards, Irvine, CA, USA), zeroed at the level of the tragus [16, 17].

rSO_2_ was recorded using NIRS regional oximetry of the left and right frontal lobes (INVOS 5100C or 7100, Covidien-Medtronic, Minneapolis, MN), whenever possible. At the discretion of the treating clinician, PbtO_2_ was monitored using an intra-parenchymal brain tissue oxygenation probe (Licox Brain Tissue Oxygen Monitoring System; Integra LifeSciences Corp., Plainsboro, New Jersey), placed in the frontal lobe. All available high-frequency full wave-form physiology was then recorded in digital time-series from the patients’ bedside ICU monitors using Intensive Care Monitoring “Plus” software (ICM+) (Cambridge Enterprise Ltd, Cambridge, UK, http://icmplus.neurosurg.cam.ac.uk) using either direct digital data transfer or analog-to-digital signal conversion (DT9804/DT9826, Data Translations, Marlboro, MA, USA).

### Data processing

All post-acquisition signal processing was performed using ICM+ software. To ensure quality of raw data, signal artifacts were removed by qualified personnel without knowledge of the study objectives or patient demographics. This involved removing segments of data that lacked proper waveform morphology or had implausibly high/low values. For any cases where ICP was monitored using an EVD, drain opening artifacts were addressed via manual curation. For each patient who received NIRS monitoring, a single rSO_2_ signal was obtained. The NIRS channel/side used was chosen based on radiographic information, when available, in order to avoid signal interference from hematomas or contusions underlying the sensors. In cases where both channels had such underlying injuries, neither channel was used. When both channels were viable, the signals were averaged to obtain a single rSO_2_ signal.

Following artifact clearing, pulse amplitude of ICP (AMP) was calculated by conducting Fourier analysis of the fundamental amplitude of the ICP pulse waveform over sequential 10-s windows of data [18, 19]. A 10-s non-overlapping moving average filter was applied to down-sample ICP, ABP (producing mean arterial pressure [MAP]), and AMP, in order to focus on the frequency range associated with cerebral vasomotion and minimize the effects of the respiratory cycle [13, 20, 21]. Then, CPP was calculated by subtracting ICP from MAP; CPP = MAP–ICP.

Three ICP-based cerebrovascular reactivity indices were derived: the pressure reactivity index (PRx—correlation between ICP and MAP), the pulse amplitude index (PAx—correlation between AMP and MAP), and RAC (the correlation (R) between AMP (A) and CPP (C)) [18, 22, 23]. These metrics were calculated as the Pearson correlations of 300-s windows of ICP and MAP (for PRx), AMP and MAP (for PAx), and AMP and CPP (for RAC), continuously updating every minute [22–26]. Two NIRS-based cerebrovascular reactivity indices were also derived in a similar manner, in accordance with the convention of recent literature: COx (correlation between rSO_2_ and CPP) and COx_a (correlation between rSO_2_ and ABP) [27–30]. Additionally, RAP (the correlation (R) between AMP (A) and ICP (P)) was calculated as a representative metric for cerebral compliance [19, 31]. Finally, all data was down-sampled to minute-by-minute resolution and output as a comma-separated values (CSV) file for each individual patient before further processing in R Statistical Computing software (R Core Team (2020). R: A language and environment for statistical computing. R Foundation for Statistical Computing, Vienna, Austria. URL https://www.R-project.org/).

### Data analysis

All statistical tests employed in this study were chosen prior to running any analyses. R Statistical Computing software was used to conduct all data analysis. The following add-on R packages were leveraged for the purposes of this analysis: *purr, dplyr, ggplot2, plotly, reticulate,* and *clinfun*. First, mean values of all physiologic metrics were computed for each patient over their entire recording period. Next, time spent with various cerebral physiologic metrics, with known associations with patient outcomes, above/below literature defined thresholds were calculated to represent cerebral physiologic insult burden. The thresholds that were used can be found in Supplemental Appendix A.

A summary of the cerebral physiologic insult burden and demographics of the entire patient cohort was then created using medians and interquartile ranges (IQR), or raw counts, where appropriate. Patients were then dichotomized based on 6-month outcome into the following: Alive (GOS > 1) versus Dead (GOS = 1) and Favorable (GOS > 3) versus Unfavorable (GOS ≤ 3) [32]. Physiologic and demographic differences between these outcome cohorts were then evaluated using Mann–Whitney U and Chi-square testing, for continuous and non-continuous variables, respectively. Next, patients were trichotomized based on mean values for each of the multimodal cerebral physiologic parameters of interest: ICP, CPP, PRx, PAx, RAC, PbtO_2_, and rSO_2_. For each patient, the mean values used for trichotomization were calculated by taking the average of all minute-by-minute data points of the parameter of interest across the patient’s entire recording period. The trichotomization groupings can be found in Supplemental Appendix B.

Jonckheere–Terpstra testing was then used to evaluate whether a directional relationship exists between these parameters and various measures of cerebral physiologic insult burden. Histograms were also created to illustrate the spread in insult burden metrics between the groupings of each trichotomization. Stacked bar plots were also produced to compare the distribution of % time spent with cerebral physiologic metrics in various ranges between the trichotomized groupings. Finally, contour plots were produced to demonstrate the effects that cerebrovascular reactivity has on the relationships between multimodal cerebral physiologic parameters and the insult burden metrics. Data points outside of two standard deviations from the mean value of each axis were excluded during the generation of these plots. This was done to reduce the area of the plots that needed to be interpolated. Data points used to produce these plots were overlayed to allow for visual inspection of the distribution of the data. Alpha was set to 0.05 for all statistical tests. Unadjusted *p*-values will be presented throughout the text; however, *p*-values corrected for multiple comparisons using the false discovery rate (FDR) method will also be presented in the tables [33–35].

## Results

### Patient population

A total of 369 patient datasets from the CAHR-TBI research collaborative were included in this analysis (120 originating from the University of Calgary, 125 from the University of Manitoba, 51 from the University of Maastricht, and 77 from the University of British Columbia). All patients had their ICP and ABP invasively monitored; however, only 146 (40%) and 116 (31%) patients had their rSO_2_ and PbtO_2_ monitored, respectively. The median age of this cohort was 38 years (IQR = 24–55) with approximately 78% of patients being male. All patients suffered a moderate-to-severe TBI, with a median admission GCS of 6 (IQR = 3–7). This cohort exhibited a 6-month mortality rate of 36%, with 77% of those who survived having a favorable outcome. A complete summary of the cerebral physiology and demographics of this patient cohort can be found in Supplemental Appendix C.

The results of the Mann–Whitney U/Chi-square testing comparing the *Alive* versus *Dead* and *Favorable* versus *Unfavorable* groupings can be found in Supplemental Appendix D. Older age, lower admission GCS, greater Marshal computerized tomography grade, greater mean values and time spent above thresholds of ICP and ICP-based cerebrovascular reactivity indices, lower mean CPP, and less time spent with CPP above 70 mmHg were seen in the non-survivors. Similarly, for the *Unfavorable* group, older age, lower admission GCS, and greater mean values and time spent above thresholds of ICP and ICP-based cerebrovascular reactivity indices were observed.

### ICP

Upon Jonckheere–Terpstra testing for ICP trichotomization, it was found that increasing ICP had a directional relationship with greater values in the following cerebral physiologic insult burden metrics: % time spent with CPP < 60 mmHg (*p* = 0.002), % time spent with PRx above its literature defined thresholds (*p* values range between 0.014 and 0.018), % time with PAx above its thresholds (*p* values range between 0.010 and 0.022), and % time spent with RAP > 0.4 (*p* = 0.036). Neither % time with RAC > 0 nor time spent above either of the NIRS-based cerebrovascular reactivity indices were able to demonstrate such an association. Interestingly, % time with CPP > 70 mmHg was found to be greater at lower ICP levels (*p* = 0.048). These findings can be found in Table 1.

**Table 1:** Jonckheere–Terpstra Testing for ICP Trichotomization

Variable	ICP < 20 mmHg (n = 337)	20 mmHg ≤ ICP ≤ 25 mmHg (n = 26)	ICP > 25 mmHg (n = 10)	J–T statistic	*p*-value	Adjusted *p*-value
Age (years)	39 (24.2–55)	33.5 (25–55)	27 (19–39.8)	5518	0.306	0.453
Sex (% male)	78.87%	61.54%	90%	5645	0.290	0.446
Admission GCS	6 (4–7)	6 (3–7)	3 (3–3.25)	3764	0.100	0.191
Admission GCS—motor	4 (1–5)	4 (1–5)	1 (1–1.75)	2779	0.466	0.583
Admission pupil response (% bilaterally reactive)	15.90%	3.85%	30%	6190	0.736	0.818
Marshall CT grade	3 (2–5)	3 (2–3)	4 (3.25–4)	5835	0.930	0.930
GOS	4 (1–5)	1 (1–2.5)	1 (1–1)	2879	**0.002**	**0.009**
Number with hypoxic episode	26.50%	35.29%	50%	2649	0.178	0.297
Number with hypotensive episode	15.08%	18.75%	33.33%	2412	0.344	0.469
Duration of recording (hours)	98.4 (53.4–186)	123 (83.2–197)	23.5 (16.6–69.5)	5600	0.322	0.460
Mean MAP (mmHg)	86.9 (81.1–92.9)	97 (90.2–102)	91.2 (87.3–97.3)	9192	**0.002**	**0.009**
Mean ICP (mmHg)	11.9 (8.17–14.8)	21.2 (20.7–22.8)	33.4 (29.7–50.6)	12,392	**0.002**	**0.009**
% Time ICP > 20 mmHg	3.81 (0.485–13.9)	56.8 (49.7–62.6)	82.8 (78.5–94)	12,330	**0.002**	**0.009**
% Time ICP > 22 mmHg	2.11 (0.229–7.69)	43.3 (35–50.3)	75.9 (71.5–91.7)	12,288	**0.002**	**0.009**
Mean CPP (mmHg)	74.9 (70.9–81.4)	76.1 (67.9–80)	57.3 (46.4–66.6)	4593	**0.006**	**0.024**
% Time CPP < 60 mmHg	4.27 (1.14–9.01)	5.6 (2.11–19.8)	61.9 (32.9–66.3)	8299	**0.002**	**0.009**
% Time CPP > 70 mmHg	68.1 (51.5–86.5)	74.8 (45.3–86.5)	16.1 (10.4–37.2)	4984	**0.048**	0.096
Mean PRx	0.108 (− 0.00288–0.216)	0.135 (− 0.0105–0.287)	0.421 (0.335–0.646)	7695	**0.020**	0.050
% Time PRx > 0	60.7 (48.2–73.4)	61.6 (49.7–81.4)	84.9 (77.6–91.4)	7637	**0.016**	**0.049**
% Time PRx > 0.25	35.8 (24.6–48.6)	40.2 (25–57.2)	70.5 (62.7–83.6)	7734	**0.018**	0.050
% Time PRx > 0.35	27.1 (17.4–38.7)	31.5 (17.5–45.9)	65.1 (55.9–80)	7761	**0.014**	**0.047**
% Time ICP > 20 mmHg & PRx > 0.35	1.66 (0.215–4.88)	19.6 (11.7–30.7)	61 (41.9–73.8)	11,711	**0.002**	**0.009**
Mean PAx	− 0.0269 (− 0.123–0.097)	0.0208 (− 0.137–0.253)	0.271 (0.0578–0.56)	7620	**0.026**	0.058
% Time PAx > 0	46.8 (34.9–61.7)	49.8 (32.9–80)	73.5 (57.5–90.3)	7655	**0.020**	0.050
% Time PAx > 0.2	26.2 (16.5–40.3)	33.2 (15.8–59)	57.2 (34.4–82.5)	7727	**0.022**	0.052
% Time PAx > 0.25	21.6 (13.7–34.9)	29.3 (12.8–53)	53.7 (28.6–80.1)	7760	**0.010**	**0.036**
% Time ICP > 20 mmHg & PAx > 0.25	1.27 (0.133–4.02)	18.7 (7.92–36.6)	41.8 (23–72.7)	11,707	**0.002**	**0.009**
Mean RAC	− 0.29 (− 0.447 to − 0.109)	− 0.332 (− 0.522 to − 0.176)	− 0.0242 (− 0.114 to 0.225)	6940	0.206	0.330
% Time RAC > 0	21.8 (11.8–36.6)	18.8 (8.46–33)	50.4 (36.8–62.9)	7016	0.156	0.271
% Time ICP > 20 mmHg & RAC > 0	0.705 (0.0888–2.41)	10.6 (4.58–26.2)	38.4 (30.5–60.9)	11,528	**0.002**	**0.009**
Mean RAP	0.668 (0.513–0.773)	0.707 (0.573–0.804)	0.732 (0.658–0.781)	7093	0.150	0.271
% Time RAP > 0.40	82 (68.3–90.5)	86.4 (75.3–94.6)	90.2 (83.2–93)	7501	**0.036**	0.076
rSO2*	70.1 (64.3–75.6)	67.6 (58.8–75)	70.8 (70.8–70.8)	293	0.652	0.767
Mean COx*	0.0164 (− 0.0203–0.0758)	− 0.0045 (− 0.00788–0.00588)	0.159 (0.159–0.159)	335	0.852	0.897
% Time COx > 0.20*	23.6 (17.8–31.2)	13.3 (12.4–17.5)	41.3 (41.3–41.3)	272	0.376	0.485
Mean COx_a*	0.0611 (0.0147–0.114)	0.00657 (0.00593–0.022)	0.166 (0.166–0.166)	217	0.598	0.725
% Time COx_a > 0.20*	28.8 (22–37.5)	16.1 (14.8–23.4)	37.2 (37.2–37.2)	190	0.352	0.469
Mean PbtO_2_ (mmHg)†	22.9 (13.8–31.2)	25.4 (20.6–31.7)	13.2 (9.74–19)	917	0.792	0.856
% Time PbtO_2_ < 15 mmHg†	7.27 (1.6–72.2)	3.46 (0.955–10.6)	61.7 (28.1–93)	924	0.730	0.818
% Time PbtO_2_ < 20 mmHg†	30.5 (7.26–91.2)	15.8 (6.09–33.2)	74.7 (50.9–99.4)	970	0.912	0.930

*ABP* arterial blood pressure, *AMP* pulse amplitude of ICP, *COx* cerebral oxygenation index (correlation between rSO2 and CPP), *COx_a* cerebral oxygenation index (correlation between rSO2 and ABP), *CPP* cerebral perfusion pressure, *CT* computed tomography, *GCS* Glasgow Coma Scale, *ICP* intracranial pressure, *IQR* interquartile range, *J–T* Jonckheere–Terpstra test, *MAP* mean arterial pressure, *PAx* pulse amplitude index (correlation between AMP and MAP), *PbtO*_2_ brain tissue oxygen tension, *PRx* pressure reactivity index (correlation between ICP and MAP), *RAC* correlation (R) between slow waves of AMP (A) and CPP (C), *RAP* compensatory reserve index (correlation between AMP and ICP), *rSO*_2_ regional cerebral oxygen saturation

Both unadjusted and adjusted *p* values are presented. Adjusted *p* values were calculated using the False Discovery Rate (FDR) method. Bolded *p* values are those reaching statistical significance, *p* < 0.05

*Only 146 patients had rSO_2_ data available

†Only 116 patients had PbtO_2_ data available

### CPP

Upon Jonckheere–Terpstra testing for CPP trichotomization, which is summarized in Table 2, it was observed that decreasing CPP demonstrates a directional relationship with greater % time with PRx, PAx, and RAC above thresholds (*p* values range between 0.002 and 0.042), and % time with PbtO_2_ < 15 and 20 mmHg (*p* = 0.014, 0.004). Notably, CPP did not demonstrate a directional relationship with time spent with ICP above thresholds. When trichotomization was done using higher thresholds, creating groupings of < 70 mmHg, 70–80 mmHg, and > 80 mmHg, very similar results can be observed. The results of this sub-analysis can be found in Supplemental Appendix E.

**Table 2:** Jonckheere–Terpstra Testing for CPP Trichotomization

Variable	CPP < 60 mmHg (n = 16)	60 mmHg ≤ CPP ≤ 70 mmHg (n = 69)	CPP > 70 mmHg (n = 288)	J–T statistic	*p*-value	Adjusted *p*-value
Age (years)	40.5 (21.8–56.2)	35 (23–55)	39 (25–55)	13,521	0.330	0.426
Sex (% male)	81.25%	65.22%	80.84%	14,198	**0.026**	0.061
Admission GCS	3 (3–7)	6 (4–7)	6 (3–7)	10,746	0.748	0.787
Admission GCS—motor	4 (1–5)	4 (3–5)	3 (1–5)	5148	0.148	0.211
Admission pupil response (% bilaterally reactive)	26.67%	13.04%	15.41%	13,736	**0.028**	0.062
Marshall CT grade	3.5 (3–4)	4.5 (2–5)	3 (2–5)	9782	**0.020**	0.053
GOS	1 (1–1)	4 (1–5)	4 (1–5)	11,376	0.352	0.426
Number with hypoxic episode	50%	30.61%	25.90%	4538	0.280	0.373
Number with hypotensive episode	25%	10.20%	17.07%	5065	0.500	0.541
Duration of recording (hours)	36.3 (16.8–105)	71.3 (45.9–116)	114 (66.1–200)	16,893	**0.002**	**0.009**
Mean MAP (mmHg)	82.1 (73.3–87.3)	80.2 (76.9–84)	90.5 (85.2–95.8)	21,025	**0.002**	**0.009**
Mean ICP (mmHg)	21.6 (16.5–32.9)	12.9 (9.68–16.1)	12.3 (8.16–15.5)	9905	**0.004**	**0.013**
% Time ICP > 20 mmHg	63.9 (25.2–82.7)	1.97 (0.226–18.6)	5.01 (0.905–17)	11,456	0.112	0.172
% Time ICP > 22 mmHg	44.9 (21.8–75.4)	0.899 (0.0863–11.6)	2.57 (0.47–10.4)	12,047	0.418	0.464
Mean CPP (mmHg)	56.7 (51.8–58.6)	67.5 (65.3–68.7)	77.3 (73.4–82.7)	25,584	**0.002**	**0.009**
% Time CPP < 60 mmHg	65.1 (52.5–77)	15.2 (9.92–23.5)	3.12 (0.822–6.06)	1766	**0.002**	**0.009**
% Time CPP > 70 mmHg	9.34 (5.54–13.9)	32.6 (26.8–41.4)	74.3 (61.6–89.9)	25,299	**0.002**	**0.009**
Mean PRx	0.417 (0.293–0.566)	0.14 (0.0376–0.253)	0.0921 (− 0.0161–0.212)	9190	**0.002**	**0.009**
% Time PRx > 0	84.9 (72.2–90)	64 (55–76.7)	59.4 (46.7–71)	9321	**0.002**	**0.009**
% Time PRx > 0.25	70.5 (57.8–78.8)	40.8 (26.8–56.8)	34.9 (23.6–47.1)	9475	**0.002**	**0.009**
% Time PRx > 0.35	63.8 (50–75.5)	30.1 (21.2–45.1)	26.4 (16.6–37)	9588	**0.002**	**0.009**
% Time ICP > 20 mmHg & PRx > 0.35	34.2 (12.8–63.2)	1.25 (0.0843–7.38)	2.13 (0.388–6.1)	11,075	0.050	0.087
Mean PAx	0.173 (0.0292–0.377)	0.0171 (− 0.171–0.16)	− 0.0311 (− 0.125–0.0967)	10,970	**0.042**	0.080
% Time PAx > 0	72.8 (53.1–84.8)	49 (33.9–68.7)	46 (35.1–61.5)	10,943	**0.026**	0.061
% Time PAx > 0.2	51.2 (32.4–73.9)	29.5 (16–44.7)	25.2 (16.5–40.5)	10,928	**0.042**	0.080
% Time PAx > 0.25	43.4 (28.2–70.1)	25.1 (12.6–39.4)	21 (13.5–34.9)	10,946	**0.034**	0.072
% Time ICP > 20 mmHg & PAx > 0.25	17 (6.78–51.9)	0.676 (0.0888–6.06)	1.76 (0.226–4.64)	11,392	0.130	0.193
Mean RAC	− 0.109 (− 0.242–0.142)	− 0.242 (− 0.44 to − 0.0153)	− 0.302 (− 0.467 to − 0.146)	9993	**0.004**	**0.013**
% Time RAC > 0	37.5 (25.4–64.1)	23.2 (12.5–47.4)	21.6 (10.9–34.1)	10,532	**0.012**	**0.037**
% Time ICP > 20 mmHg & RAC > 0	21.7 (4.63–47.7)	0.493 (0.0281–3.41)	0.882 (0.14–2.68)	11,232	0.068	0.113
Mean RAP	0.655 (0.49–0.727)	0.627 (0.534–0.746)	0.68 (0.517–0.787)	14,353	0.086	0.138
% Time RAP > 0.40	81.1 (66.3–87.7)	79.3 (70.2–90.1)	83 (71.1–91)	13,639	0.356	0.426
rSO_2_*	70.8 (65.4–74)	69.8 (61.6–75)	70.4 (64.7–76.1)	2042	0.394	0.450
Mean COx*	0.108 (− 0.008–0.109)	0.0242 (− 0.00451–0.0705)	0.012 (− 0.025–0.0714)	1816	0.362	0.426
% Time COx > 0.20*	35.7 (18.5–36.2)	22.1 (15.1–30.5)	24 (17.6–31)	2060	0.860	0.860
Mean COx_a*	0.0854 (0.0528–0.109)	0.0437 (0.00813–0.118)	0.0599 (0.0226–0.111)	1815	0.768	0.788
% Time COx_a > 0.20*	28.4 (24.3–33.2)	26.3 (18.4–37.1)	29 (23.3–37.7)	2028	0.186	0.257
Mean PbtO_2_ (mmHg)†	19 (13.2–31.2)	14.9 (9.98–19.9)	24 (15.5–31.7)	900	**0.044**	0.080
% Time PbtO_2_ < 15 mmHg†	28.1 (1.88–61.7)	89.3 (41.7–95.3)	5.93 (1.5–61.9)	434	**0.014**	**0.040**
% Time PbtO_2_ < 20 mmHg†	50.9 (23.2–74.7)	96.4 (87.1–98.9)	26 (6.24–88.4)	397	**0.004**	**0.013**

*AMP* pulse amplitude of ICP, *COx* cerebral oxygenation index (correlation between rSO_2_ and CPP), *COx_a* cerebral oxygenation index (correlation between rSO_2_ and ABP), *CPP* cerebral perfusion pressure, *CT* computed tomography, *GCS* Glasgow Coma Scale, *ICP* intracranial pressure, *IQR* interquartile range, *J–T* Jonckheere–Terpstra test, *MAP* mean arterial pressure, *PAx* pulse amplitude index (correlation between AMP and MAP), *PbtO*_*2*_ brain tissue oxygen tension, *PRx* pressure reactivity index (correlation between ICP and MAP), *RAC* correlation (R) between slow waves of AMP (A) and CPP (C), *RAP* compensatory reserve index (correlation between AMP and ICP), *rSO*_*2*_ regional cerebral oxygen saturation

Both unadjusted and adjusted *p* values are presented. Adjusted *p* values were calculated using the False Discovery Rate (FDR) method. Bolded *p* values are those reaching statistical significance, *p* < 0.05

*Only 146 patients had rSO_2_ data available

^†^Only 116 patients had PbtO_2_ data available

### Cerebrovascular reactivity metrics

Testing for PRx trichotomization demonstrated that, with increasing PRx, there is greater % time with CPP < 60 mmHg (*p* = 0.030), greater % time with RAP > 0.40 (*p* = 0.002), greater % time with PbtO_2_ below its thresholds (*p* = 0.024, 0.040), and less % time with CPP > 70 mmHg (*p* = 0.002). However, no directional relationship with ICP was found. These findings are summarized in Table 3. Increasing PAx demonstrated similar results; however, was found to be associated with greater % time with ICP above thresholds (*p* = 0.032, 0.040) and not with % time with RAP > 0.40. The Jonckheere–Terpstra testing results for PAx can be found in Supplemental Appendix F. RAC yielded differing results, only demonstrating directional relationships with less % time with CPP > 70 mmHg (*p* = 0.004) and greater % time with RAP > 0.40 (*p* = 0.002). The summary for these results can be found in Supplemental Appendix G.

**Table 3:** Jonckheere–Terpstra Testing for PRx Trichotomization

Variable	Intact (PRx < 0) (n = 95)	Transitional (0 ≤ PRx ≤ 0.20) (n = 164)	Deranged (PRx > 0.20) (n = 114)	J–T statistic	*p*-value	Adjusted *p*-value
Age (years)	33.5 (22.2–50.8)	37 (25–56)	42 (25–55)	24,310	0.062	0.092
Sex (% male)	77.89%	77.44%	78.76%	22,557	0.886	0.933
Admission GCS	6 (3–8)	6 (4–7)	6 (3–7)	18,716	0.778	0.871
Admission GCS—motor	3 (1–5)	4 (1–5)	4 (1–5)	10,511	0.384	0.466
Admission pupil response (% bilaterally reactive)	15.22%	12.96%	19.27%	19,485	0.052	0.080
Marshall CT grade	3 (2–4)	3 (2–5)	3 (2–5)	23,339	**0.004**	**0.009**
GOS	4 (1–5)	4 (1–5)	2 (1–5)	16,177	**0.006**	**0.013**
Number with hypoxic episode	18.03%	28.57%	35.94%	8973	**0.022**	**0.042**
Number with hypotensive episode	13.56%	14.29%	20.31%	8253	0.302	0.378
Duration of recording (hours)	141 (75.9–242)	96.9 (55.3–168)	74.7 (39.3–165)	17,879	**0.002**	**0.005**
Mean MAP (mmHg)	91 (82.6–96.7)	87.2 (81.8–93.8)	86.5 (81.1–91.7)	19,263	**0.002**	**0.005**
Mean ICP (mmHg)	13.3 (9.67–17.2)	11.8 (8.17–15)	13.3 (8.72–17.7)	22,446	0.960	0.960
% Time ICP > 20 mmHg	6.62 (1.31–24.3)	3.53 (0.385–15.7)	6.43 (0.852–29.3)	22,787	0.834	0.902
% Time ICP > 22 mmHg	3.6 (0.872–14)	2.08 (0.188–9.21)	3.62 (0.421–18.2)	22,642	0.926	0.950
Mean CPP (mmHg)	76.6 (71.4–81.9)	75.3 (70.8–82)	73.2 (67.5–78)	18,331	**0.002**	**0.005**
% Time CPP < 60 mmHg	3.88 (1.35–7.97)	3.92 (0.848–8.16)	6.05 (1.78–17.1)	25,150	**0.030**	0.050
% Time CPP > 70 mmHg	75.2 (57.3–89.3)	69.3 (52.7–88.3)	58.9 (33.5–74)	17,821	**0.002**	**0.005**
Mean PRx	− 0.0814 (− 0.131 to − 0.0345)	0.101 (0.0512–0.14)	0.321 (0.251–0.434)	45,106	**0.002**	**0.005**
% Time PRx > 0	38.6 (34.3–44)	60.2 (55–64.3)	81.7 (76.5–87.8)	44,708	**0.002**	**0.005**
% Time PRx > 0.25	19 (14.7–22.4)	35.4 (29.9–40)	61.9 (53.9–72.9)	44,587	**0.002**	**0.005**
% Time PRx > 0.35	13.2 (9.95–16.2)	26.7 (22.2–30.5)	51.3 (42.2–65.2)	44,147	**0.002**	**0.005**
% Time ICP > 20 mmHg & PRx > 0.35	1.75 (0.468–4.21)	1.63 (0.205–5.42)	4.76 (0.367–17)	26,227	**0.004**	**0.009**
Mean PAx	− 0.147 (− 0.228 to − 0.0881)	− 0.0285 (− 0.118–0.0576)	0.129 (0.0408–0.289)	36,995	**0.002**	**0.005**
% Time PAx > 0	31.4 (23.8–38.4)	46.7 (35.4–57.3)	66 (56.4–78.1)	37,366	**0.002**	**0.005**
% Time PAx > 0.2	15.7 (11–19.8)	25.3 (17.6–34.3)	44.6 (34.6–60.4)	37,642	**0.002**	**0.005**
% Time PAx > 0.25	12.5 (8.51–16.1)	21.3 (14.5–29.4)	39.6 (30.4–55.7)	37,549	**0.002**	**0.005**
% Time ICP > 20 mmHg & PAx > 0.25	1.62 (0.218–4.74)	1.25 (0.139–3.66)	2.98 (0.227–12.6)	25,148	**0.026**	**0.045**
Mean RAC	− 0.471 (− 0.59 to − 0.328)	− 0.302 (− 0.44 to − 0.16)	− 0.0977 (− 0.275 to 0.0843)	34,604	**0.002**	**0.005**
% Time RAC > 0	10.2 (5.93–19.7)	21.7 (12.9–32.1)	39.1 (23.5–58.6)	34,525	**0.002**	**0.005**
% Time ICP > 20 mmHg & RAC > 0	0.877 (0.212–2.46)	0.713 (0.0856–2.53)	1.67 (0.221–9.23)	25,595	**0.014**	**0.028**
Mean RAP	0.74 (0.635–0.839)	0.66 (0.507–0.757)	0.627 (0.47–0.723)	16,727	**0.002**	**0.005**
% Time RAP > 0.40	89.4 (80–94.8)	81.7 (67.6–89.2)	78.9 (65.2–88.5)	17,177	**0.002**	**0.005**
rSO_2_*	70.4 (64.6–74.5)	69.8 (64.2–76.6)	70.8 (63.4–75.6)	2997	0.784	0.871
Mean COx*	0 (− 0.0229–0.0488)	0.0166 (− 0.0109–0.0909)	0.0178 (− 0.00187–0.0726)	3689	0.152	0.212
% Time COx > 0.20*	21.5 (13.2–28.1)	24.5 (18.3–33.3)	22.3 (18.1–31.1)	3660	0.228	0.304
Mean COx_a*	0.0485 (0.012–0.0946)	0.0572 (0.0145–0.117)	0.0721 (0.0136–0.117)	3003	0.286	0.369
% Time COx_a > 0.20*	26.7 (20.2–32.5)	29.1 (23.5–37.4)	28.5 (21.4–37.7)	2962	0.538	0.633
Mean PbtO_2_ (mmHg)†	25.3 (21.7–31.7)	22.7 (13.5–31.6)	20 (13.7–27.7)	1874	0.154	0.212
% Time PbtO_2_ < 15 mmHg†	1.77 (0.893–10.6)	12.1 (1.72–75.1)	17.5 (4.17–80.8)	2594	**0.024**	**0.044**
% Time PbtO_2_ < 20 mmHg†	15.8 (5.93–36.5)	37 (5.09–94.2)	50 (23.2–90.7)	2568	**0.040**	0.064

*AMP* pulse amplitude of ICP, *COx* cerebral oxygenation index (correlation between rSO_2_ and CPP), *COx_a* cerebral oxygenation index (correlation between rSO_2_ and ABP), *CPP* cerebral perfusion pressure, *CT* computed tomography, *GCS* Glasgow Coma Scale, *ICP* intracranial pressure, *IQR* interquartile range, *J–T* Jonckheere–Terpstra test, *MAP* mean arterial pressure, *PAx* pulse amplitude index (correlation between AMP and MAP), *PbtO*_2_ brain tissue oxygen tension, *PRx* pressure reactivity index (correlation between ICP and MAP), *RAC* correlation (R) between slow waves of AMP (A) and CPP (C), *RAP* compensatory reserve index (correlation between AMP and ICP), *rSO*_2_ regional cerebral oxygen saturation

Both unadjusted and adjusted *p* values are presented. Adjusted *p* values were calculated using the False Discovery Rate (FDR) method. Bolded *p* values are those reaching statistical significance, *p* < 0.05

*Only 146 patients had rSO_2_ data available

^†^Only 116 patients had PbtO_2_ data available

### Oxygen delivery metrics

Next, Jonckheere–Terpstra testing for PbtO_2_ found that, with decreasing values, there was greater % time with CPP < 60 mmHg (*p* = 0.002), less % time with CPP > 70 mmHg (*p* = 0.002), greater % time with PAx and RAC above their literature defined thresholds (*p* values range between 0.020 and 0.048), and greater % time with COx > 0.20 (*p* = 0.020). These results can be found in Supplemental Appendix H. Testing for rSO_2_ only demonstrated a directional relationship with greater % time with PAx above its thresholds (*p* values range between 0.036 and 0.049), see Supplemental Appendix I.

### Generated plots

**Fig. 1:**
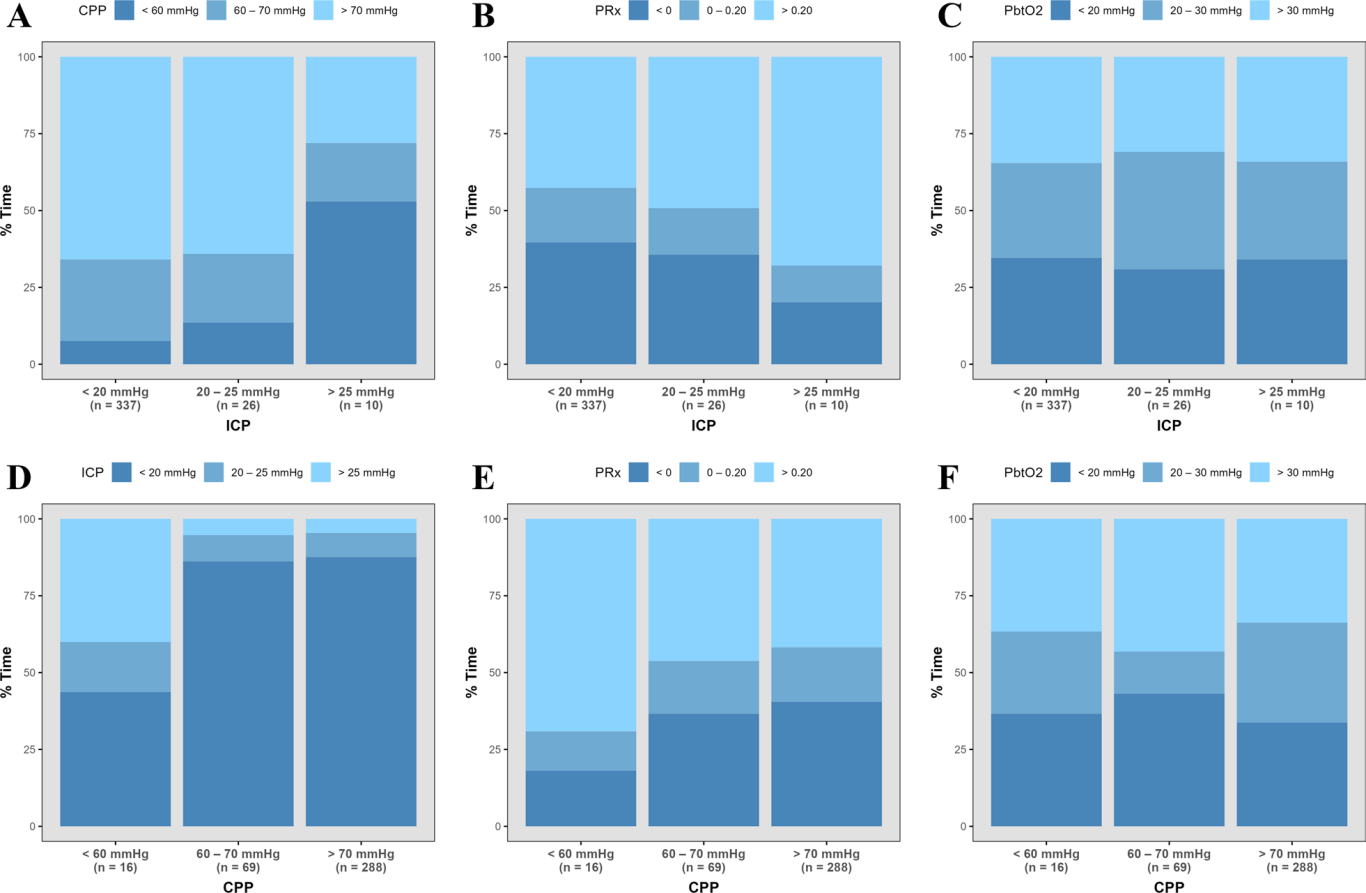
Stacked bar plots comparing cerebral physiology between patients trichotomized using mean ICP or CPP. **A** comparison of % times with CPP in three ranges between patients trichotomized using mean ICP; **B** comparison of % times with PRx in three ranges between patients trichotomized using mean ICP; **C** comparison of % times with PbtO_2_ in three ranges between patients trichotomized using mean ICP; **D** comparison of % times with ICP in three ranges between patients trichotomized using mean CPP; **E** comparison of % times with PRx in three ranges between patients trichotomized using mean CPP; **F** comparison of % times with PbtO_2_ in three ranges between patients trichotomized using mean CPP. *AMP* pulse amplitude of ICP, *CPP* cerebral perfusion pressure, *ICP* intracranial pressure, *MAP* mean arterial pressure, *PbtO*_2_ brain tissue oxygen tension, *PRx* pressure reactivity index (correlation between ICP and MAP)

Histograms illustrating the spread of various insult burden metrics between the three groupings for ICP, CPP, PRx, PbtO_2_, and rSO_2_ can be found in Supplemental Appendices J–N. In Fig. 1 we display stacked bar plots that compare the distribution of % time with key cerebral physiologic metrics in various ranges between trichotomized patients. Panels A–C demonstrate that with increasing ICP, there tends to be greater % time with CPP < 60 mmHg and PRx > 0.20, but that no clear directional relationship with PbtO_2_ is observable. Panels D–F illustrate that with low CPP, there tends to be greater % time with ICP > 25 mmHg, and PRx > 0.20, but again, no clear directional relationship with PbtO_2_ is observed.

**Fig. 2:**
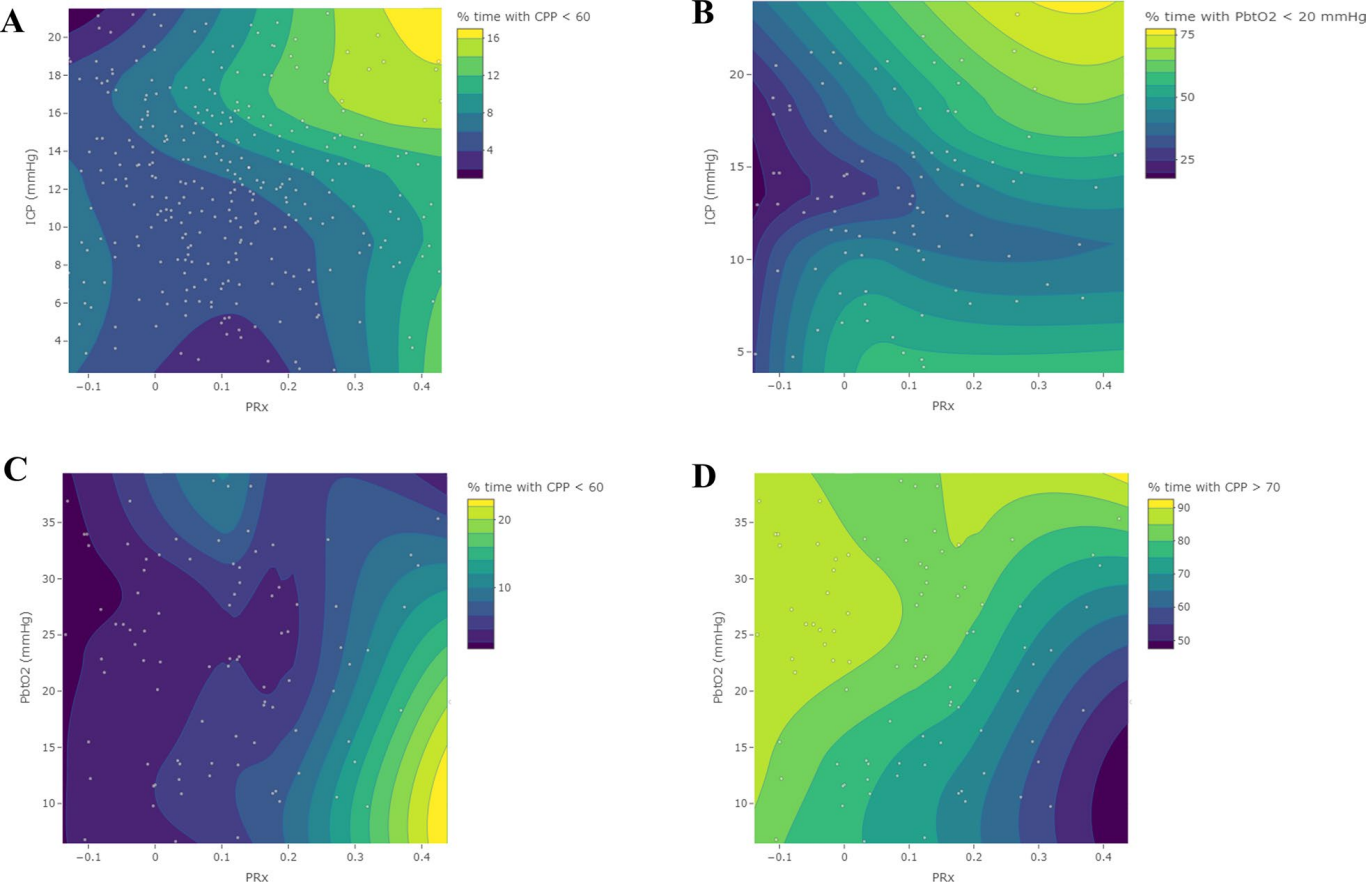
Contour plots demonstrating the protective effects of preserved cerebrovascular reactivity on global cerebral insult burden. Plots illustrate the effects of PRx on the relationship between: **A** ICP and % time with CPP < 60 mmHg; **B** ICP and % time with PbtO_2_ < 20 mmHg; **C** PbtO_2_ and % time with CPP < 60 mmHg; and **D** PbtO_2_ and % time with CPP > 70 mmHg. Data points used to construct the contour plots are overlayed on each plot. *CPP* cerebral perfusion pressure, *ICP* intracranial pressure, *PbtO*_2_ brain tissue oxygen tension, *PRx* pressure reactivity index

Contour plots demonstrating the effects that cerebrovascular reactivity intactness has on the relationship between the above mentioned physiologic metrics and metrics of insult burden can be found in Supplemental Appendices O–R. Figure 2 displays some of the key plots that demonstrate the protective effects of intact cerebrovascular reactivity on insult burden. Panels A and B show that greater mean ICP is associated with more time spent with CPP < 60 mmHg as well as more time spent with PbtO_2_ < 20 mmHg, but that these relationships are stronger when PRx is positive and diminish when PRx is negative. Similarly, panel C illustrates that low PbtO_2_ is associated with greater % time with CPP < 60 mmHg when PRx is deranged. Interestingly, panel D shows that % time with CPP > 70 mmHg is associated with high PbtO_2_, and that intact PRx maintains this relationship to a further extent.

## Discussion

In this descriptive analysis on the effects of derangements in continuous multimodal cerebral physiology on cerebral physiologic insult burden, we were able to uncover multiple interesting findings that deserve highlighting. Firstly, we found that increasing ICP is associated with increasing burden of cerebral hypoperfusion, deranged cerebrovascular reactivity, and reduced cerebral compliance. This is in keeping with the findings of a 2020 study by Zeiler and colleagues, where the authors compared cerebral physiology between patients with mean ICP < 15 mmHg and those with mean ICP > 20 mmHg [23]. This study, however, found that elevated ICP was also associated with worse PbtO_2_, while our study failed to find such an association. This discrepancy could potentially be explained by the low proportion of patients in our cohort who had PbtO_2_ monitored. In total, only 116 patients had this metric monitored during their ICU stay and, therefore, analyses involving PbtO_2_ may have been too underpowered to undercover this relationship.

Next, our analysis indicated that decreasing CPP is associated with increased time suffering from deranged cerebrovascular reactivity and low PbtO_2_. The fact that these directional relationships were found is interesting, as it suggests that higher mean CPP is associated with less cerebral physiologic insult burden. This is further suggested by finding that cerebral hyperperfusion, as defined as CPP > 70 mmHg, was associated with lower mean ICP. This is somewhat surprising, considering that current management guidelines recommend maintaining CPP below this threshold, suggesting that it contributes to secondary brain injury [10, 11]. Further, it was previously believed that having CPP above 70 mmHg may be associated with a range of systematic complications, such as acute respiratory distress syndrome (ARDS). However, these findings are actually in keeping with recent literature that suggests that, though preventing hypoperfusion is vital, preventing hyperemic CPP may not significantly affect outcomes [36, 37]. Thiara et al*.* found that time spent with CPP above CPP optimal (CPPopt) is not associated with acute respiratory distress syndrome (ARDS) [37]. Furthermore, a recent study by our group even suggested that spending time with elevated CPP may actually be associated with improvement in outcome, possibly due to the injured brain requiring greater blood flow to fulfil increased metabolic activity associated with injury repair [38]. This contributes to an increasing body of literature that questions the utility of a 70 mmHg CPP threshold. Additionally, the association found between low ICP and more cerebral hyperperfusion can be explained with the CPP derivation formula: CPP = MAP–ICP. Interestingly, no association between CPP and burden of intracranial hypertension was uncovered. This may be explained by the fact that ICP is highly controlled during moderate-to-severe TBI management, with our cohort having an average % time with elevated ICP of only about 5%.

Our analysis of the three ICP-based cerebrovascular reactivity indices yielded unexpectedly differing results. Increasing (deranged) PRx was found to be associated with more time suffering from cerebral hypoperfusion, poor cerebral compliance (as measured using RAP), and low PbtO_2_, and less time with cerebral hyperperfusion. This supports the growing narrative that PRx monitoring can provide valuable insight that can help minimize secondary brain injury [39, 40]. Increasing PAx displayed somewhat similar results but was also found to be associated with more time suffering from intracranial hypertension; however, was not found to be associated with poor cerebral compliance. Increasing mean RAC, on the other hand, was only found to be related to less time with cerebral hyperperfusion and greater time with poor cerebral compliance. These inconsistent findings suggest that these three indices offer different insights into a patient’s cerebral physiology and have different utility. This variation may be explained by the fact that RAC reflects both cerebrovascular reactivity and cerebral compliance [23], and that PAx and RAC (but not PRx) utilize AMP for derivation. The ICP-based cerebrovascular reactivity indices did not demonstrate associations with NIRS-based indices. This suggests that these NIRS-based indices may not closely mirror ICP-based indices for the evaluation of cerebral autoregulatory status.

Looking at the results for PbtO_2_ trichotomization, it was shown that decreasing mean values were only associated with more time suffering from cerebral hypoperfusion, more time with PAx and RAC above thresholds, more time with COx above threshold, and less time with cerebral hyperperfusion. Overall, it seems that PbtO_2_ offers mixed insight into cerebral physiologic insult burden. This finding is supported by a recent study by Svedung Wettervik and colleagues, which could only find weak associations between cerebral physiologic variables and PbtO_2_ [41]. It is interesting that an association was found with PAx and RAC, but not with PRx; however, this may again be due to the low sample size of patients with PbtO_2_ monitoring. It is possible that the lack of association between PbtO_2_ and insult burden metrics can be partially explained by the fact that low/deranged PbtO_2_ can often be relatively quickly treated with oxygen supplementation, thus reducing the incidence of low PbtO_2_ with other deranged cerebral physiology. This could potentially weaken any relationships between PbtO_2_ and measures of cerebral physiologic insult burden. The lack of associations between this cerebral oxygen delivery measure and insult burden metrics may also suggest that different pathophysiologic mechanisms are at play. It is possible that deranged cerebral oxygen delivery causes insult through separate mechanisms from other drivers of secondary brain injury. However, their lack of associations with outcomes, as seen in Supplemental Appendix D, also suggests a general lack of association with secondary brain injury.

With regard to PbtO_2_, it is important to acknowledge the complex nature of this measure, which is influenced by a wide range of physiological factors. PbtO_2_ is not solely a reflection of cerebral oxygen delivery. While it is influenced by arterial oxygen tension (PbtO_2_) and CBF, PbtO_2_ is better described as an indicator of the balance between regional oxygen supply and cellular oxygen consumption [42]. A large range of factors can contribute to changes in this balance. Factors such as the diffusion distance between capillaries and cells, the location of the probe relative to arterioles and venules, hemoglobin concentration, vascular reactivity, metabolic demand, and secondary brain injury can all contribute to variations in PbtO_2_ readings. Understanding the complexity of the interplay of factors influencing PbtO_2_ is crucial for interpreting PbtO_2_ results.

Testing for rSO_2_ demonstrated even poorer results, with no associations with any of the cerebral physiologic insult burden metrics, except time spent with PAx above its thresholds. This suggests that raw rSO_2_ values offer limited insight into secondary brain injury. A recent study from the CAHR-TBI consortium by Gomez and colleagues supports this finding as we found that raw rSO_2_ demonstrates limited prognostic utility [27]. Though there is some interest in the clinical application of NIRS in TBI management, the role of NIRS in this setting is arguably very limited as a result of various major limitations of the technology. NIRS devices use near-infrared light, which is able to penetrate through the scalp and skull into the cerebral parenchyma where it is absorbed and reflected by hemoglobin in the microvasculature. Then by detecting the intensity of light, at specific wavelengths, that is scattered and reflected back to the device, the relative concentrations of oxygenated and deoxygenated hemoglobin can be determined [43, 44]. These relative concentrations can then be used to derive the regional cerebral oxygen saturation (rSO_2_). The most significant limitation of NIRS technology arises from the problem of extracerebral interference from sources such as the scalp, bone, cerebrospinal fluid, and subdural blood [45]. There is significant homogeneity in these structures between patients, as well as across the skull. This results in relatively large amounts of variation in signal depending on the placement of the pads and patient-specific factors. This raises questions about the validity of NIRS-based values and their clinical utility.

Finally, the generated contour plots strongly suggest that intact cerebrovascular reactivity has protective effects on the relationships uncovered in this analysis. This finding has been highlighted in multiple recent studies [9, 46–48], promoting the importance of maintaining cerebrovascular reactivity in order to minimize secondary brain insult.

This study provides a unique comprehensive assessment of the various associations that cerebral physiologic derangements have with cerebral physiologic insult burden. The results above shed unique light on the important relationship that cerebral physiology as a whole has with secondary brain injury following moderate-to-severe TBI. We have provided supporting evidence of the importance of ICP, CPP, and cerebrovascular reactivity control in minimizing secondary brain injury. Our analysis also raises some questions of the utility of PbtO_2_ and rSO_2_ monitoring, in their raw signal output form, in detecting and protecting against secondary brain injury.

### Limitations

Despite the important findings of this analysis, a couple of important limitations must be highlighted. Firstly, though this study utilized a large multi-centered cohort, only 116 patients had PbtO_2_ monitored and were included in analyses involving this parameter. This may have limited our ability to find associations between PbtO_2_ and other cerebral physiologic parameters. Next, our analysis utilized grand-averaged values. Such values fail to accurately reflect the state of the parameter over the patient’s time in the ICU. For example, a patient who has extremely high CPP for half their recording period and extremely low CPP for the other half, may have a relatively moderate mean CPP value. Additionally, acute extreme derangements in physiology can be diluted if the recording period is long enough. Thus, future works should focus on using metrics that better reflect the actual cerebral physiologic picture of patients, such as dose-time above/below thresholds. Another limitation of this study is that it could have included more continuous multimodal cerebral physiologic monitoring modalities, such as electroencephalogram and bispectral index score. This could have offered a fuller understanding of the effects of deranged cerebral physiology on insult burden.

## Conclusion

In this multi-centered descriptive analysis, we evaluated the associations between derangements in continuous multimodal cerebral physiology and cerebral physiologic insult burden. We found that mean ICP, CPP, and ICP-based cerebrovascular reactivity indices demonstrated statistically significant associations with global cerebral physiologic insult burden. However, it remains unclear whether measures of oxygen delivery provide any meaningful insight into such insult burden.

## Supplementary Information

Supplementary Material 1.

## Data Availability

The datasets analyzed in this study are currently not publicly available as the Canadian and EU jurisdictions, including the research ethics boards and regional privacy bodies under which data was collected, do not allow for data sharing.

## Abbreviations

AMP: Pulse amplitude of ICP
ABP: Arterial blood pressure
BTF: Brain Trauma Foundation
CAHR-TBI: Canadian High Resolution-TBI Research Collaborative
COx: Correlation between rSO_2_ and CPP
COx_a: Correlation between rSO_2_ and ABP
CPP: Cerebral perfusion pressure
EVD: External ventricular drain
GCS: Glasgow Coma Scale
GOS: Glasgow Outcome Scale
ICM+: Intensive Care Monitoring “Plus” software
ICP: Intracranial pressure
ICU: Intensive care unit
IQR: Interquartile range
FDR: False discovery rate
MAP: Mean arterial pressure
NIRS: Near-infrared spectroscopy
PbtO_2_: Brain tissue oxygenation
PAx: Pulse amplitude index
PRx: Pressure reactivity index
RAC: Correlation (R) between AMP (A) and CPP (C)
RAP: The correlation (R) between AMP (A) and ICP (P)
rSO_2_: Regional cerebral oxygen saturation
TBI: Traumatic brain injury
