# Supplementary material for: Cerebral physiologic insult burden in acute traumatic neural injury: a Canadian High Resolution-TBI (CAHR-TBI) descriptive analysis
Source: Crit Care. 2024 Sep 4;28:294. doi: 10.1186/s13054-024-05083-y (PMC11373089; doi:10.1186/s13054-024-05083-y)
Supplement: Supplementary file 1 — Supplementary Material 1. [file 13054_2024_5083_MOESM1_ESM.docx]

**Supplemental Material**

**Tables of Contents:**

| **A** | Thresholds used for deriving cerebral physiologic insult burden metrics | 3 |
| --- | --- | --- |
| **B** | Trichotomization groupings for cerebral physiologic metrics | 4 |
| **C** | Patient Cohort Demographics and Cerebral Physiology | 5 |
| **D** | Mann-Whitney U/Chi-Square Testing for Alive vs Dead and Favorable vs Unfavorable | 6-7 |
| **E** | Jonckheere-Terpstra Testing for CPP Trichotomization with Alternative Thresholds | 8-9 |
| **F** | Jonckheere-Terpstra Testing for PAx Trichotomization | 10-11 |
| **G** | Jonckheere-Terpstra Testing for RAC Trichotomization | 12-13 |
| **H** | Jonckheere-Terpstra Testing for PbtO_2_ Trichotomization | 14-15 |
| **I** | Jonckheere-Terpstra Testing for rSO_2_ Trichotomization | 16-17 |
| **J** | Insult Burden Histograms for ICP Trichotomization | 18 |
| **K** | Insult Burden Histograms for CPP Trichotomization | 19 |
| **L** | Insult Burden Histograms for PRx Trichotomization | 20 |
| **M** | Insult Burden Histograms for PbtO_2_ Trichotomization | 21 |
| **N** | Insult Burden Histograms for rSO_2_ Trichotomization | 22 |
| **O** | Contour Plots Demonstrating the Effects of Cerebrovascular Reactivity on the Relationship Between ICP and Global Cerebral Insult Burden | 23 |
| **P** | Contour Plots Demonstrating the Effects of Cerebrovascular Reactivity on the Relationship Between CPP and Global Cerebral Insult Burden | 24 |
| **Q** | Contour Plots Demonstrating the Effects of Cerebrovascular Reactivity on the Relationship Between PbtO_2_ and Global Cerebral Insult Burden | 25 |
| **R** | Contour Plots Demonstrating the Effects of Cerebrovascular Reactivity on the Relationship Between rSO_2_ and Global Cerebral Insult Burden | 26 |

Supplemental Appendix A. Thresholds used for deriving cerebral physiologic insult burden metrics

| **Cerebral Physiologic Variable** | **Thresholds** |
| --- | --- |
| ICP | > 20 mmHg, > 22 mmHg |
| CPP | < 60 mmHg, > 70 mmHg |
| PRx | > 0 a.u., > 0.25 a.u., > 0.35 a.u. |
| PAx | > 0 a.u., > 0.20 a.u., > 0.25 a.u. |
| RAC | > 0 a.u. |
| RAP | > 0.40 a.u. |
| PbtO_2_ | < 15 mmHg, < 20 mmHg |
| COx | > 0.20 a.u. |
| COx_a | > 0.20 a.u. |

*ABP = arterial blood pressure, AMP = pulse amplitude of ICP, COx = cerebral oxygenation index (correlation between rSO2 and CPP), COx_a = cerebral oxygenation index (correlation between rSO2 and ABP), CPP = cerebral perfusion pressure, ICP = intracranial pressure, MAP = mean arterial pressure, PAx = pulse amplitude index (correlation between AMP and MAP), PbtO2 = brain tissue oxygen tension, PRx = pressure reactivity index (correlation between ICP and MAP), RAC = correlation (R) between slow waves of AMP (A) and CPP (C), RAP = compensatory reserve index (correlation between AMP and ICP), rSO2 = regional cerebral oxygen saturation.*

Supplemental Appendix B. Trichotomization groupings for cerebral physiologic metrics

| **Cerebral Physiologic Variable** | **Trichotomization Grouping** |
| --- | --- |
| ICP | < 20 mmHg, 20 – 25 mmHg, > 25 mmHg |
| CPP | < 60 mmHg, 60 – 70 mmHg, > 70 mmHg |
| PRx, PAx, RAC | < 0 a.u., 0 – 0.2 a.u., > 0.2 a.u. |
| PbtO_2_ | < 20 mmHg, 20 – 30 mmHg, > 30 mmHg |
| rSO_2_ | < 60 mmHg, 60 – 70 mmHg, > 70 mmHg |

*ABP = arterial blood pressure, AMP = pulse amplitude of ICP, CPP = cerebral perfusion pressure, ICP = intracranial pressure, MAP = mean arterial pressure, PAx = pulse amplitude index (correlation between AMP and MAP), PbtO2 = brain tissue oxygen tension, PRx = pressure reactivity index (correlation between ICP and MAP), RAC = correlation (R) between slow waves of AMP (A) and CPP (C), rSO2 = regional cerebral oxygen saturation.*

Supplemental Appendix C. Patient Cohort Demographics and Cerebral Physiology

| **Variable** | | **Median (IQR) or Raw Numbers (%)** |
| --- | --- | --- |
| Number of Patients | | 373 |
| Age (years) | | 38 (24 – 55) |
| Sex | Male | 290 (78%) |
|  | Female | 82 (22%) |
| Admission GCS | | 6 (3 – 7) |
| Admission GCS – Motor | | 4 (1 – 5) |
| Admission Pupil Response | Bilaterally Reactive | 244 (67%) |
|  | Unilaterally Unreactive | 64 (18%) |
|  | Bilaterally Unreactive | 56 (15%) |
| Marshall CT Score | | 3 (2 – 5) |
| GOS | | 3 (1 – 5) |
| Number Alive (GOS > 1) | | 214 (64%) |
| Number Dead (GOS = 1) | | 122 (36%) |
| Number Favorable (GOS 4 – 5) | | 167 (50%) |
| Number Unfavorable (GOS 1 – 3) | | 169 (50%) |
| Number with Hypoxia Episode | | 62 (28%) |
| Number with Hypotension Episode | | 35 (16%) |
| Recording Duration (hours) | | 98.2 (51.62 – 184.5) |
| Mean MAP (mmHg) | | 87.37 (81.8 – 93.98) |
| Mean ICP (mmHg) | | 12.51 (8.622 – 16.04) |
| % Time ICP > 20 mmHg | | 5.245 (0.8306 – 19.1) |
| % Time ICP > 22 mmHg | | 2.604 (0.3292 – 11.64) |
| Mean CPP (mmHg) | | 74.72 (70.2 – 80.96) |
| % Time CPP < 60 mmHg | | 4.645 (1.378 – 9.843) |
| % Time CPP > 70 mmHg | | 67.95 (48.88 – 85.96) |
| Mean PRx | | 0.11 (-0.002602 – 0.2341) |
| % Time PRx > 0 | | 61.54 (48.59 – 74.4) |
| % Time PRx > 0.25 | | 36.33 (24.8 – 50.84) |
| % Time PRx > 0.35 | | 27.65 (17.43 – 39.62) |
| % Time ICP > 20 mmHg & PRx > 0.35 | | 2.209 (0.3338 – 6.667) |
| Mean PAx | | -0.02027 (-0.1251 – 0.1096) |
| % Time PAx > 0 | | 47.33 (35.09 – 63.61) |
| % Time PAx > 0.20 | | 26.47 (16.38 – 42.42) |
| % Time PAx > 0.25 | | 22.05 (13.59 – 36.17) |
| % Time ICP > 20 mmHg & PAx > 0.25 | | 1.762 (0.1739 – 5.866) |
| Mean RAC | | -0.29 (-0.45 – -0.1064) |
| % Time RAC > 0 | | 21.99 (11.56 – 37.94) |
| % Time ICP > 20 mmHg & RAC > 0 | | 0.9327 (0.1038 – 3.148) |
| Mean RAP | | 0.6753 (0.5172 – 0.7734) |
| % Time RAP > 0.40 | | 82.36 (70.88 – 90.8) |
| rSO_2_ | | 70.11 (64.19 – 75.6) |
| Mean COx | | 0.015 (-0.01966 – 0.07537) |
| % Time COx > 0.20 | | 23.54 (17.4 – 31.18) |
| Mean COx_a | | 0.05993 (0.01399 – 0.1142) |
| % Time COx_a > 0.20 | | 28.84 (21.65 – 37.23) |
| Mean PbtO2 (mmHg) | | 22.98 (13.79 – 31.37) |
| % Time PbtO2 < 15 mmHg | | 6.976 (1.543 – 72.25) |
| % Time PbtO2 < 20 mmHg | | 28.93 (7.103 – 91.37) |

*AMP = pulse amplitude of ICP, COx = cerebral oxygenation index (correlation between rSO_2_ and CPP), COx_a = cerebral oxygenation index (correlation between rSO2 and ABP), CPP = cerebral perfusion pressure, CT = computed tomography, GCS = Glasgow Coma Scale, GOS = Glasgow Outcome Scale, ICP = intracranial pressure, IQR = interquartile range, LLR = lower limit of reactivity, MAP = mean arterial pressure, PAx = pulse amplitude index (correlation between AMP and MAP), PbtO2 = brain tissue oxygen tension, PRx = pressure reactivity index (correlation between ICP and MAP), RAC = correlation (R) between slow waves of AMP (A) and CPP (C), RAP = compensatory reserve index (correlation between AMP and ICP), rSO_2_ = regional cerebral oxygen saturation, ULR = upper limit of reactivity.*

Supplemental Appendix D. Mann-Whitney U/Chi-Square Testing for Alive vs Dead and Favorable vs Unfavorable

| **Variable** | **Alive/Dead Outcome Groups** | | | | **Favorable/Unfavorable Outcome Groups** | | | |
| --- | --- | --- | --- | --- | --- | --- | --- | --- |
|  | **Alive**  **Median (IQR)** | **Dead**  **Median (IQR)** | **p-value** | **Adjusted p-value** | **Favorable**  **Median (IQR)** | **Unfavorable**  **Median (IQR)** | **p-value** | **Adjusted p-value** |
| Age (years) | 33.5 (23 – 47.8) | 50 (28 – 63) | **<0.0001** | **<0.0001** | 34 (23 – 46.5) | 44 (25 – 60) | **<0.0001** | **0.0001** |
| Sex (% Male) | 77.60% | 79.50% | 0.7820 | 0.8026 | 78.40% | 78.10% | 1.0000 | 1.0000 |
| Admission GCS | 6 (4 – 8) | 5 (3 – 7) | **0.0026** | **0.0056** | 7 (5 – 8) | 5 (3 – 7) | **<0.0001** | **<0.0001** |
| Admission GCS – Motor | 4 (2 – 5) | 3 (1 – 5) | 0.1440 | 0.2160 | 4 (2.25 – 5) | 2 (1 – 5) | **0.0018** | **0.0036** |
| Admission Pupil Response  (% Bilaterally Reactive) | 68.70% | 61.50% | 0.3140 | 0.4223 | 73.70% | 58.60% | **0.0227** | **0.0422** |
| Marshall CT Grade | 3 (2 – 5) | 4 (2.5 – 5) | **0.0032** | **0.0065** | 3 (2 – 5) | 3 (2 – 5) | 0.2200 | 0.3432 |
| Number with Hypoxic Episode | 20.56% | 13.93% | 0.3620 | 0.4554 | 20.96% | 15.38% | 0.9970 | 1.0000 |
| Number with Hypotensive Episode | 13.55% | 4.92% | **0.0445** | 0.0789 | 13.17% | 7.69% | 0.6800 | 0.7577 |
| Duration of Recording (hours) | 94.6 (49.3 – 166) | 97 (51.9 – 173) | 0.6810 | 0.7378 | 86 (46.3 – 154) | 111 (62.7 – 189) | **0.0102** | **0.0199** |
| Mean MAP (mmHg) | 86.9 (81.8 – 94.7) | 88.2 (81 – 93.9) | 0.9410 | 0.9410 | 86.3 (80.9 – 93.4) | 88.4 (81.9 – 94.8) | 0.0916 | 0.1624 |
| Mean ICP (mmHg) | 11.7 (8.2 – 14.8) | 14 (9.06 – 19.1) | **0.0003** | **0.0007** | 11.4 (7.59 – 14.6) | 13.3 (9.32 – 17.8) | **0.0002** | **0.0006** |
| % Time ICP > 20 mmHg | 4.27 (0.449 – 14.1) | 9.68 (1.18 – 41.4) | **0.0004** | **0.0009** | 3.18 (0.265 – 14.2) | 8.88 (1.59 – 32.3) | **0.0001** | **0.0002** |
| % Time ICP > 22 mmHg | 2.3 (0.243 – 8.01) | 4.66 (0.598 – 27.6) | **0.0004** | **0.0010** | 1.72 (0.104 – 7.61) | 4 (0.802 – 19) | **0.0001** | **0.0003** |
| Mean CPP (mmHg) | 75.4 (71.2 – 81.7) | 73.7 (69.8 – 79.8) | **0.0135** | **0.0263** | 75.4 (71.1 – 81.3) | 74.1 (70.1 – 81) | 0.2670 | 0.3719 |
| % Time CPP < 60 mmHg | 4.56 (1.18 – 8.07) | 4.82 (1.46 – 13.6) | 0.1080 | 0.1685 | 4.65 (1.47 – 8.72) | 4.72 (1.13 – 10.1) | 0.5810 | 0.6664 |
| % Time CPP > 70 mmHg | 69.6 (53.9 – 87.8) | 61.7 (42.6 – 82.7) | **0.0252** | **0.0468** | 69.4 (53.6 – 85.3) | 65.1 (45.5 – 86.6) | 0.3750 | 0.4570 |
| Mean PRx | 0.0862 (-0.0127 – 0.201) | 0.176 (0.0585 – 0.311) | **<0.0001** | **0.0001** | 0.0731 (-0.0122 – 0.186) | 0.148 (0.0326 – 0.29) | **0.0001** | **0.0003** |
| % Time PRx > 0 | 58 (46.2 – 70.9) | 66.8 (55.9 – 81.2) | **<0.0001** | **0.0001** | 57.4 (45.1 – 70.1) | 65.4 (54.2 – 80.5) | **0.0001** | **0.0004** |
| % Time PRx > 0.25 | 34.3 (23.7 – 46.3) | 42.5 (30.8 – 57.9) | **0.0001** | **0.0005** | 32.5 (23.5 – 45.1) | 41.2 (26.8 – 56.6) | **0.0004** | **0.0009** |
| % Time PRx > 0.35 | 25.2 (17.3 – 36.1) | 33.1 (22.2 – 48.3) | **0.0004** | **0.0009** | 24.6 (17.2 – 35.2) | 31.6 (19.2 – 45.9) | **0.0006** | **0.0012** |
| % Time ICP > 20 mmHg & PRx > 0.35 | 1.73 (0.21 – 4.86) | 3.93 (0.629 – 14.2) | **0.0001** | **0.0005** | 1.52 (0.0974 – 4.76) | 3.17 (0.739 – 10.9) | **<0.0001** | **0.0001** |
| Mean PAx | -0.0433 (-0.156 – 0.0769) | 0.0572 (-0.0645 – 0.215) | **<0.0001** | **<0.0001** | -0.0457 (-0.146 – 0.0578) | 0.0255 (-0.0973 – 0.176) | **<0.0001** | **<0.0001** |
| % Time PAx > 0 | 44.4 (32.4 – 59.1) | 57.4 (41.5 – 73.6) | **<0.0001** | **<0.0001** | 43.8 (32.3 – 58.2) | 54.9 (39.1 – 70.6) | **<0.0001** | **<0.0001** |
| % Time PAx > 0.2 | 23.6 (16 – 37.9) | 34.2 (22 – 51.3) | **<0.0001** | **<0.0001** | 23.6 (16 – 36.4) | 32.2 (20 – 49.9) | **<0.0001** | **<0.0001** |
| % Time PAx > 0.25 | 19.6 (12.6 – 32.6) | 29.7 (19.1 – 46.6) | **<0.0001** | **<0.0001** | 19.6 (12.6 – 30.8) | 27.1 (16.3 – 43.8) | **<0.0001** | **<0.0001** |
| % Time ICP > 20 mmHg & PAx > 0.25 | 1.31 (0.141 – 3.95) | 3.31 (0.281 – 14.8) | **0.0002** | **0.0006** | 0.904 (0.0771 – 3.62) | 2.56 (0.38 – 9.46) | **<0.0001** | **0.0001** |
| Mean RAC | -0.33 (-0.474 – -0.149) | -0.223 (-0.346 – -0.00772) | **<0.0001** | **<0.0001** | -0.327 (-0.492 – -0.16) | -0.238 (-0.384 – -0.0285) | **0.0001** | **0.0002** |
| % Time RAC > 0 | 19.9 (9.78 – 33.9) | 28 (19.5 – 47.8) | **<0.0001** | **<0.0001** | 20.2 (9.44 – 32.8) | 26.7 (16.5 – 46.6) | **<0.0001** | **0.0001** |
| % Time ICP > 20 mmHg & RAC > 0 | 0.713 (0.0872 – 2.36) | 1.78 (0.193 – 8.84) | **0.0001** | **0.0004** | 0.564 (0.0174 – 1.87) | 1.55 (0.287 – 6.63) | **<0.0001** | **<0.0001** |
| Mean RAP | 0.676 (0.518 – 0.773) | 0.65 (0.507 – 0.768) | 0.5590 | 0.6229 | 0.68 (0.526 – 0.787) | 0.656 (0.508 – 0.754) | 0.2650 | 0.3719 |
| % Time RAP > 0.40 | 82.8 (70.3 – 90.4) | 80.4 (68.5 – 91.9) | 0.7530 | 0.7937 | 84 (69.1 – 90.9) | 81.2 (69.3 – 89.9) | 0.3530 | 0.4570 |
| rSO2 | 70 (64.5 – 76.5) | 69.8 (63.1 – 74.7) | 0.4040 | 0.4924 | 70 (64.6 – 76.3) | 69.8 (63.4 – 75.1) | 0.5670 | 0.6664 |
| Mean COx | 0.0121 (-0.0376 – 0.0669) | 0.0264 (-0.00559 – 0.109) | 0.0643 | 0.1090 | 0.0134 (-0.0318 – 0.0651) | 0.0201 (-0.0108 – 0.0949) | 0.2530 | 0.3719 |
| % Time COx > 0.20 | 23.2 (18.4 – 30.7) | 24.5 (18.4 – 35.6) | 0.3140 | 0.4223 | 24.3 (19.7 – 30.5) | 24.4 (15.9 – 32.6) | 0.8920 | 0.9663 |
| Mean COx_a | 0.0575 (0.0147 – 0.109) | 0.0568 (0.00934 – 0.121) | 0.5130 | 0.6063 | 0.0577 (0.0174 – 0.11) | 0.0532 (0.00832 – 0.12) | 0.9260 | 0.9761 |
| % Time COx_a > 0.20 | 29.4 (22 – 36.8) | 26.7 (20.6 – 37.5) | 0.5420 | 0.6217 | 29.4 (23.5 – 36.8) | 26.7 (19.6 – 37.2) | 0.2820 | 0.3792 |
| Mean PbtO2 (mmHg) | 22.9 (14.2 – 31.7) | 18.9 (11.9 – 27.7) | 0.0963 | 0.1565 | 22.9 (13.8 – 32.6) | 19.7 (12.5 – 28.6) | 0.1520 | 0.2577 |
| % Time PbtO2 < 15 mmHg | 7.48 (1.56 – 73.6) | 21.4 (1.87 – 86.8) | 0.3360 | 0.4368 | 7.4 (1.45 – 69.8) | 12 (1.8 – 84.9) | 0.3710 | 0.4570 |
| % Time PbtO2 < 20 mmHg | 30.9 (5.47 – 91.8) | 66.7 (12.5 – 97.8) | 0.1790 | 0.2586 | 27.4 (4.35 – 91.5) | 52.6 (12.6 – 97.2) | 0.1750 | 0.2844 |

*Both unadjusted and adjusted p values are presented. Adjusted p values were calculated using the False Discovery Rate (FDR) method. Bolded p values are those reaching statistical significance, p < 0.05.*

*ABP = arterial blood pressure, AMP = pulse amplitude of ICP, COx = cerebral oxygenation index (correlation between rSO2 and CPP), COx_a = cerebral oxygenation index (correlation between rSO2 and ABP), CPP = cerebral perfusion pressure, CT = computed tomography, GCS = Glasgow Coma Scale, ICP = intracranial pressure, IQR = interquartile range, MAP = mean arterial pressure, PAx = pulse amplitude index (correlation between AMP and MAP), PbtO2 = brain tissue oxygen tension, PRx = pressure reactivity index (correlation between ICP and MAP), RAC = correlation (R) between slow waves of AMP (A) and CPP (C), RAP = compensatory reserve index (correlation between AMP and ICP), rSO2 = regional cerebral oxygen saturation.*

Supplemental Appendix E. Jonckheere-Terpstra Testing for CPP Trichotomization with Alternative Thresholds

| **Variable** | **CPP < 60 mmHg**  **[n = 16]** | **60 mmHg ≤ CPP ≤ 70 mmHg**  **[n = 69]** | **CPP > 70 mmHg**  **[n = 288]** | **J-T Statistic** | **p-value** | **Adjusted p-value** |
| --- | --- | --- | --- | --- | --- | --- |
| Age (years) | 35 (22 - 55) | 40 (25 - 57) | 38 (25 - 50) | 21834.5 | 0.762 | 0.847 |
| Sex (% Male) | 68.24% | 80.22% | 81.90% | 23450.5 | 0.034 | 0.068 |
| Admission GCS | 6 (4 - 7) | 6 (3 - 8) | 6 (3 - 7) | 17091 | 0.542 | 0.638 |
| Admission GCS – Motor | 4 (2 - 5) | 3 (1 - 5) | 3 (1 - 4) | 8695 | 0.042 | 0.073 |
| Admission Pupil Response  (% Bilaterally Reactive) | 15.48% | 16.38% | 13.73% | 22386 | 0.068 | 0.105 |
| Marshall CT Grade | 4 (2.25 - 5) | 3 (2 - 4) | 3 (2 - 5) | 18464.5 | 0.148 | 0.211 |
| GOS | 3 (1 - 5) | 4 (1 - 5) | 3 (1 - 5) | 18466 | 0.458 | 0.573 |
| Number with Hypoxic Episode | 33.33% | 22.64% | 31.67% | 7846.5 | 0.940 | 0.990 |
| Number with Hypotensive Episode | 12.28% | 11.43% | 27.12% | 8481.5 | **0.040** | 0.073 |
| Duration of Recording (hours) | 65 (40.5 - 116) | 120 (65.6 - 228) | 103 (66.4 - 184) | 24969 | **0.004** | **0.016** |
| Mean MAP (mmHg) | 80.2 (76 - 85.2) | 86.7 (82.6 - 91.2) | 96.1 (92.7 - 101) | 36865 | **0.002** | **0.010** |
| Mean ICP (mmHg) | 13.6 (9.91 - 18.1) | 12.5 (8.44 - 15.7) | 11.5 (7.23 - 15.3) | 18371 | **0.002** | **0.010** |
| % Time ICP > 20 mmHg | 8.73 (0.356 - 33.7) | 5.75 (1.11 - 18.2) | 4.32 (0.8 - 15.4) | 19973 | 0.064 | 0.102 |
| % Time ICP > 22 mmHg | 3.56 (0.121 - 19) | 2.67 (0.555 - 11.5) | 2.39 (0.207 - 9.22) | 20656 | 0.324 | 0.428 |
| Mean CPP (mmHg) | 67.1 (63.3 - 68.6) | 74.4 (72.4 - 76.8) | 84.3 (82.1 - 88.8) | 43695 | **0.002** | **0.010** |
| % Time CPP < 60 mmHg | 19 (11.2 - 33.3) | 4.89 (2.92 - 7.71) | 0.627 (0.139 - 1.79) | 4315 | **0.002** | **0.010** |
| % Time CPP > 70 mmHg | 29.2 (18.8 - 39.3) | 65.4 (57.1 - 74) | 93.2 (88 - 97.8) | 42762.5 | **0.002** | **0.010** |
| Mean PRx | 0.178 (0.0605 - 0.325) | 0.0981 (-0.0146 - 0.237) | 0.0818 (-0.0165 - 0.151) | 17706 | **0.002** | **0.010** |
| % Time PRx > 0 | 68.8 (57 - 81.9) | 59.5 (47 - 74.9) | 58.4 (46.2 - 66.4) | 18183 | **0.004** | **0.016** |
| % Time PRx > 0.25 | 43.1 (30.1 - 62.8) | 35 (23.8 - 50.8) | 33.3 (23.5 - 42.7) | 18108 | **0.002** | **0.010** |
| % Time PRx > 0.35 | 32.8 (22.8 - 53.9) | 27 (16.2 - 41.2) | 25.2 (17.3 - 33.4) | 18116 | **0.002** | **0.010** |
| % Time ICP > 20 mmHg & PRx > 0.35 | 3.14 (0.179 - 15) | 2.49 (0.482 - 6.62) | 1.65 (0.215 - 4.42) | 19249.5 | **0.014** | **0.040** |
| Mean PAx | 0.025 (-0.128 - 0.175) | -0.0175 (-0.121 - 0.108) | -0.0414 (-0.129 - 0.0511) | 19403 | **0.030** | 0.063 |
| % Time PAx > 0 | 54.7 (34.8 - 72.7) | 47.9 (35.1 - 63) | 43.8 (35.2 - 58.2) | 19559 | **0.048** | 0.080 |
| % Time PAx > 0.2 | 32.1 (16.3 - 50) | 26.8 (16.2 - 42.9) | 23.9 (17.7 - 33.9) | 19488 | **0.030** | 0.063 |
| % Time PAx > 0.25 | 26.4 (14 - 45) | 22.2 (13.3 - 37.9) | 19.4 (14.2 - 28.2) | 19483 | **0.020** | 0.053 |
| % Time ICP > 20 mmHg & PAx > 0.25 | 2.29 (0.122 - 10.2) | 1.75 (0.29 - 4.95) | 1.77 (0.144 - 4.3) | 20095 | 0.124 | 0.184 |
| Mean RAC | -0.222 (-0.373 - -0.00687) | -0.298 (-0.468 - -0.144) | -0.335 (-0.453 - -0.148) | 18836 | **0.010** | 0.033 |
| % Time RAC > 0 | 27.4 (13.1 - 49.1) | 21.7 (10.8 - 34) | 20.9 (11.9 - 35) | 19501 | **0.030** | 0.063 |
| % Time ICP > 20 mmHg & RAC > 0 | 1.46 (0.0719 - 7.12) | 1.05 (0.208 - 2.78) | 0.739 (0.0892 - 2.66) | 19615.5 | **0.038** | 0.072 |
| Mean RAP | 0.629 (0.53 - 0.741) | 0.698 (0.567 - 0.791) | 0.652 (0.464 - 0.775) | 21989 | 0.966 | 0.991 |
| % Time RAP > 0.40 | 80.4 (70.2 - 89.8) | 85.8 (73.2 - 91.4) | 79.8 (62.4 - 89.2) | 21095 | 0.496 | 0.601 |
| rSO_2_ | 69.9 (61.7 - 75) | 70.7 (66.3 - 75.6) | 67.9 (64.3 - 76.5) | 2986 | 0.748 | 0.847 |
| Mean COx | 0.0307 (-0.00662 - 0.108) | 0.0149 (-0.0201 - 0.0727) | 0.0102 (-0.0574 - 0.0491) | 2953.5 | 0.274 | 0.378 |
| % Time COx > 0.20 | 22.3 (15.3 - 33.6) | 24.1 (18.4 - 30) | 23.2 (14.3 - 31.8) | 3239.5 | 0.992 | 0.992 |
| Mean COx_a | 0.0585 (0.00813 - 0.118) | 0.062 (0.0296 - 0.116) | 0.0586 (0.0171 - 0.0964) | 2755 | 0.926 | 0.990 |
| % Time COx_a > 0.20 | 26.5 (19.4 - 37.1) | 29 (25.9 - 39.4) | 29.5 (22.5 - 36.3) | 2972 | 0.332 | 0.428 |
| Mean PbtO_2_ (mmHg) | 16 (11.1 - 23.9) | 22.5 (13.8 - 31.4) | 26 (19.8 - 32.7) | 2430 | **0.028** | 0.063 |
| % Time PbtO_2_ < 15 mmHg | 61.7 (16.6 - 93) | 12 (1.44 - 72.3) | 2.6 (1.5 - 23.3) | 1484 | **0.010** | **0.033** |
| % Time PbtO_2_ < 20 mmHg | 6.7 (1.12 - 10.5) | 11.4 (1.91 - 48.1) | 23.9 (2.66 - 62.4) | 2423 | **0.012** | **0.037** |

*Both unadjusted and adjusted p values are presented. Adjusted p values were calculated using the False Discovery Rate (FDR) method. Bolded p values are those reaching statistical significance, p < 0.05.*

*AMP = pulse amplitude of ICP, COx = cerebral oxygenation index (correlation between rSO_2_ and CPP), COx_a = cerebral oxygenation index (correlation between rSO_2_ and ABP), CPP = cerebral perfusion pressure, CT = computed tomography, GCS = Glasgow Coma Scale, ICP = intracranial pressure, IQR = interquartile range, J-T = Jonckheere-Terpstra test, MAP = mean arterial pressure, PAx = pulse amplitude index (correlation between AMP and MAP), PbtO_2_ = brain tissue oxygen tension, PRx = pressure reactivity index (correlation between ICP and MAP), RAC = correlation (R) between slow waves of AMP (A) and CPP (C), RAP = compensatory reserve index (correlation between AMP and ICP), rSO_2_ = regional cerebral oxygen saturation.*

Supplemental Appendix F. Jonckheere-Terpstra Testing for PAx Trichotomization

| **Variable** | **Intact (PAx < 0)**  **[n = 200]** | **Transitional (0 ≤ PAx ≤ 0.20)**  **[n = 120]** | **Deranged (PAx > 0.20)**  **[n = 53]** | **J-T Statistic** | **p-value** | **Adjusted p-value** |
| --- | --- | --- | --- | --- | --- | --- |
| Age (years) | 32 (22 – 48.5) | 41 (28.2 – 57) | 53 (39 – 58) | 25749 | **0.002** | **0.005** |
| Sex (% Male) | 80.50% | 73.95% | 77.36% | 19515 | 0.298 | 0.351 |
| Admission GCS | 5 (3 – 7) | 6 (4 – 7) | 6 (3.25 – 9) | 18617 | 0.058 | 0.080 |
| Admission GCS – Motor | 3 (1 – 5) | 4 (2 – 5) | 4 (2 – 5) | 10628 | **0.004** | **0.009** |
| Admission Pupil Response  (% Bilaterally Reactive) | 15.82% | 15.65% | 13.46% | 19464 | 0.846 | 0.905 |
| Marshall CT Grade | 3 (2 – 4) | 3 (2 – 5) | 3 (3 – 5) | 20914 | **0.022** | **0.038** |
| GOS | 4 (2 – 5) | 3 (1 – 5) | 1 (1 – 3.25) | 13402 | **0.002** | **0.005** |
| Number with Hypoxic Episode | 25% | 23.19% | 44.74% | 8162 | 0.090 | 0.120 |
| Number with Hypotensive Episode | 15.79% | 17.39% | 13.16% | 7361 | 0.948 | 0.948 |
| Duration of Recording (hours) | 106 (60.8 – 185) | 97.8 (48.9 – 191) | 69 (39.1 – 140) | 18321 | **0.036** | 0.053 |
| Mean MAP (mmHg) | 88.6 (82.6 – 95.5) | 86.6 (80.7 – 92) | 86.4 (81.9 – 91.2) | 18032 | **0.014** | **0.026** |
| Mean ICP (mmHg) | 12.1 (8.29 – 15.3) | 12.6 (9.18 – 15.7) | 14.9 (7.9 – 21.3) | 23008 | **0.032** | 0.051 |
| % Time ICP > 20 mmHg | 3.76 (0.962 – 15.4) | 7 (0.413 – 19.2) | 10.6 (0.727 – 56.8) | 22969 | **0.032** | 0.051 |
| % Time ICP > 22 mmHg | 2.2 (0.472 – 8.08) | 3.41 (0.225 – 11.6) | 9.74 (0.464 – 44.9) | 22758 | **0.040** | 0.057 |
| Mean CPP (mmHg) | 76 (71.4 – 82.3) | 73.7 (70 – 79.7) | 73.2 (65.5 – 78.3) | 16334 | **0.002** | **0.005** |
| % Time CPP < 60 mmHg | 3.56 (1.06 – 8.1) | 5.26 (1.71 – 9.3) | 7.38 (1.45 – 23.5) | 23525 | **0.008** | **0.015** |
| % Time CPP > 70 mmHg | 72.5 (54.2 – 89.3) | 63.9 (46.1 – 81.1) | 58.3 (25.1 – 78) | 16479 | **0.002** | **0.005** |
| Mean PRx | 0.0261 (-0.0759 – 0.112) | 0.184 (0.103 – 0.276) | 0.384 (0.243 – 0.558) | 34299 | **0.002** | **0.005** |
| % Time PRx > 0 | 51.2 (40.3 – 60.5) | 69.1 (61.9 – 79.2) | 85.3 (71 – 91) | 34560 | **0.002** | **0.005** |
| % Time PRx > 0.25 | 26.8 (19.7 – 37.4) | 45 (35.5 – 56.2) | 69.5 (50.7 – 80.2) | 33934 | **0.002** | **0.005** |
| % Time PRx > 0.35 | 19.9 (13.3 – 28.1) | 33.9 (25.9 – 45.2) | 60.5 (39.6 – 74.8) | 33424 | **0.002** | **0.005** |
| % Time ICP > 20 mmHg & PRx > 0.35 | 1.6 (0.4 – 4.22) | 3.09 (0.241 – 8.97) | 7.61 (0.284 – 31.1) | 25033 | **0.002** | **0.005** |
| Mean PAx | -0.118 (-0.202 – -0.0697) | 0.0864 (0.0385 – 0.122) | 0.318 (0.254 – 0.436) | 40960 | **0.002** | **0.005** |
| % Time PAx > 0 | 35.4 (27.5 – 41.4) | 60.7 (55.2 – 65.2) | 83 (74.9 – 91.2) | 40742 | **0.002** | **0.005** |
| % Time PAx > 0.2 | 16.9 (12.4 – 21.8) | 38.4 (32.3 – 44) | 66.4 (57 – 77.7) | 40592 | **0.002** | **0.005** |
| % Time PAx > 0.25 | 14.1 (9.83 – 18.4) | 33.3 (26.9 – 38.9) | 61.3 (52.9 – 73.7) | 40479 | **0.002** | **0.005** |
| % Time ICP > 20 mmHg & PAx > 0.25 | 1.08 (0.15 – 3.36) | 2.63 (0.172 – 7.86) | 6.64 (0.248 – 36.4) | 25858 | **0.002** | **0.005** |
| Mean RAC | -0.415 (-0.546 – -0.281) | -0.213 (-0.326 – -0.0522) | 0.11 (-0.0153 – 0.3) | 34941 | **0.002** | **0.005** |
| % Time RAC > 0 | 14.5 (7.62 – 22) | 30.2 (20.5 – 41.9) | 62.7 (47.9 – 82.3) | 35063 | **0.002** | **0.005** |
| % Time ICP > 20 mmHg & RAC > 0 | 0.695 (0.112 – 1.93) | 1.37 (0.104 – 3.96) | 6.64 (0.177 – 34.3) | 25544 | **0.002** | **0.005** |
| Mean RAP | 0.679 (0.517 – 0.799) | 0.651 (0.515 – 0.752) | 0.662 (0.518 – 0.764) | 19711 | 0.440 | 0.503 |
| % Time RAP > 0.40 | 82.8 (70.7 – 90.9) | 81.3 (70 – 90.1) | 82.8 (71.5 – 91.3) | 20693 | 0.860 | 0.905 |
| rSO2 | 70.7 (64.9 – 76.6) | 70 (64.1 – 75.6) | 67.4 (59.5 – 71.8) | 2420 | 0.140 | 0.181 |
| Mean COx | 0.0108 (-0.0196 – 0.0635) | 0.0169 (-0.0393 – 0.0688) | 0.0601 (-0.000288 – 0.162) | 3479 | 0.164 | 0.205 |
| % Time COx > 0.20 | 24.3 (15.7 – 30.6) | 22.7 (18.4 – 29.8) | 25.4 (17.1 – 41.8) | 3303 | 0.482 | 0.536 |
| Mean COx_a | 0.0518 (0.0117 – 0.11) | 0.0617 (0.0239 – 0.104) | 0.102 (0.0291 – 0.166) | 2908 | 0.226 | 0.274 |
| % Time COx_a > 0.20 | 29 (23.5 – 36.8) | 26.7 (20.9 – 37) | 29 (22.9 – 42.3) | 2668 | 0.906 | 0.929 |
| Mean PbtO2 (mmHg) | 25.4 (19.1 – 32) | 18.9 (12.6 – 26.1) | 19.2 (12.5 – 30.5) | 1483 | **0.034** | 0.052 |
| % Time PbtO2 < 15 mmHg | 3.09 (0.905 – 25.5) | 41.8 (3.62 – 86.8) | 12 (4.22 – 69.7) | 2437 | **0.006** | **0.012** |
| % Time PbtO2 < 20 mmHg | 18.2 (4.81 – 53.5) | 66.7 (15.4 – 95.2) | 49.9 (16.8 – 95.7) | 2404 | **0.006** | **0.012** |

*Both unadjusted and adjusted p values are presented. Adjusted p values were calculated using the False Discovery Rate (FDR) method. Bolded p values are those reaching statistical significance, p < 0.05.*

*ABP = arterial blood pressure, AMP = pulse amplitude of ICP, COx = cerebral oxygenation index (correlation between rSO2 and CPP), COx_a = cerebral oxygenation index (correlation between rSO2 and ABP), CPP = cerebral perfusion pressure, CT = computed tomography, GCS = Glasgow Coma Scale, ICP = intracranial pressure, IQR = interquartile range, J-T = Jonckheere-Terpstra test, MAP = mean arterial pressure, PAx = pulse amplitude index (correlation between AMP and MAP), PbtO2 = brain tissue oxygen tension, PRx = pressure reactivity index (correlation between ICP and MAP), RAC = correlation (R) between slow waves of AMP (A) and CPP (C), RAP = compensatory reserve index (correlation between AMP and ICP), rSO2 = regional cerebral oxygen saturation.*

Supplemental Appendix G. Jonckheere-Terpstra Testing for RAC Trichotomization

| **Variable** | **Intact (RAC < 0)**  **[n = 320]** | **Transitional (0 ≤ RAC ≤ 0.20)**  **[n = 33]** | **Deranged (RAC > 0.20)**  **[n = 20]** | **J-T Statistic** | **p-value** | **Adjusted p-value** |
| --- | --- | --- | --- | --- | --- | --- |
| Age (years) | 36 (24 – 52) | 55 (39 – 62) | 54.5 (39 – 57.2) | 11552 | **0.002** | **0.005** |
| Sex (% Male) | 77.43% | 84.85% | 75% | 9064 | 0.686 | 0.784 |
| Admission GCS | 6 (3 – 7) | 6 (4 – 8) | 4 (3 – 7.5) | 7768 | 0.744 | 0.804 |
| Admission GCS – Motor | 4 (1 – 5) | 3.5 (1.75 – 5) | 2.5 (1.75 – 5) | 4602 | 0.532 | 0.645 |
| Admission Pupil Response  (% Bilaterally Reactive) | 15.38% | 16.13% | 15% | 7796 | 0.386 | 0.483 |
| Marshall CT Grade | 3 (2 – 5) | 4 (3 – 5) | 4 (3 – 5) | 9992 | **0.012** | **0.027** |
| GOS | 4 (1 – 5) | 2.5 (1 – 4) | 1 (1 – 1) | 5224 | **0.002** | **0.005** |
| Number with Hypoxic Episode | 25.81% | 23.81% | 56.25% | 4078 | 0.104 | 0.166 |
| Number with Hypotensive Episode | 17.93% | 0% | 12.50% | 3167 | 0.098 | 0.163 |
| Duration of Recording (hours) | 107 (59.4 – 193) | 75.2 (49.2 – 129) | 49.4 (17.4 – 70.5) | 6010 | **0.002** | **0.005** |
| Mean MAP (mmHg) | 88 (82.2 – 94.8) | 85.3 (78.9 – 89.4) | 86.9 (82.3 – 90.2) | 7152 | **0.022** | **0.044** |
| Mean ICP (mmHg) | 12.5 (9.02 – 15.8) | 10.4 (6.41 – 13.8) | 19.1 (8.67 – 24.1) | 9127 | 0.698 | 0.784 |
| % Time ICP > 20 mmHg | 5.37 (0.99 – 18.5) | 1.22 (0.136 – 10.6) | 46.5 (0.108 – 70) | 8655 | 0.822 | 0.828 |
| % Time ICP > 22 mmHg | 2.72 (0.442 – 11.5) | 0.539 (0.0857 – 4.54) | 23.3 (0.0516 – 60.3) | 8616 | 0.814 | 0.828 |
| Mean CPP (mmHg) | 75.3 (70.9 – 81.4) | 72.8 (68.1 – 78.6) | 67.9 (62.2 – 74.5) | 6307 | **0.002** | **0.005** |
| % Time CPP < 60 mmHg | 4.38 (1.4 – 8.9) | 5.95 (0.636 – 13.4) | 8.98 (1.15 – 34.9) | 10020 | 0.092 | 0.160 |
| % Time CPP > 70 mmHg | 69.3 (53 – 86.6) | 56.5 (36.7 – 82.1) | 39.4 (15.1 – 69.3) | 6451 | **0.004** | **0.009** |
| Mean PRx | 0.0832 (-0.0174 – 0.188) | 0.276 (0.199 – 0.399) | 0.541 (0.36 – 0.729) | 15138 | **0.002** | **0.005** |
| % Time PRx > 0 | 58.2 (45.6 – 69.2) | 76.1 (72.8 – 85.8) | 91.9 (83.2 – 95.4) | 15102 | **0.002** | **0.005** |
| % Time PRx > 0.25 | 33.7 (22.8 – 45.1) | 57.9 (45.6 – 69.5) | 83.6 (66.9 – 90.8) | 14892 | **0.002** | **0.005** |
| % Time PRx > 0.35 | 25.1 (16.2 – 35.8) | 46.7 (32.8 – 62.6) | 77.9 (56.4 – 88.3) | 14744 | **0.002** | **0.005** |
| % Time ICP > 20 mmHg & PRx > 0.35 | 2.22 (0.42 – 6.33) | 0.389 (0.0843 – 7.61) | 28.5 (0.0539 – 63.8) | 9449 | 0.356 | 0.459 |
| Mean PAx | -0.0565 (-0.139 – 0.0511) | 0.219 (0.16 – 0.29) | 0.523 (0.425 – 0.627) | 16908 | **0.002** | **0.005** |
| % Time PAx > 0 | 43.3 (33.3 – 57) | 73.6 (67.2 – 78.1) | 92.5 (88.7 – 95.6) | 16833 | **0.002** | **0.005** |
| % Time PAx > 0.2 | 23.3 (16 – 34.2) | 55.3 (44.7 – 62) | 84.1 (76.4 – 89.4) | 16818 | **0.002** | **0.005** |
| % Time PAx > 0.25 | 19.2 (12.6 – 29.7) | 51.2 (39.4 – 57.2) | 81 (71.9 – 88.1) | 16818 | **0.002** | **0.005** |
| % Time ICP > 20 mmHg & PAx > 0.25 | 1.77 (0.215 – 5.01) | 0.38 (0.0562 – 5.47) | 34.8 (0.0539 – 60.1) | 9537 | 0.302 | 0.417 |
| Mean RAC | -0.339 (-0.479 – -0.205) | 0.0668 (0.0393 – 0.111) | 0.372 (0.289 – 0.518) | 17620 | **0.002** | **0.005** |
| % Time RAC > 0 | 19.5 (9.71 – 29.5) | 57.5 (54.1 – 63.3) | 85 (80.9 – 92.3) | 17589 | **0.002** | **0.005** |
| % Time ICP > 20 mmHg & RAC > 0 | 0.911 (0.133 – 2.71) | 0.513 (0.0686 – 6.64) | 37.6 (0.0539 – 61.1) | 10156 | 0.068 | 0.130 |
| Mean RAP | 0.684 (0.563 – 0.787) | 0.487 (0.355 – 0.588) | 0.606 (0.455 – 0.76) | 5565 | **0.002** | **0.005** |
| % Time RAP > 0.40 | 84 (73.3 – 91.2) | 66 (54.5 – 76.3) | 78.2 (66.1 – 90.8) | 6082 | **0.002** | **0.005** |
| rSO2 | 70.1 (64.3 – 75.6) | 73.3 (66.2 – 77.1) | 70.3 (63.4 – 72.2) | 1278 | 0.706 | 0.784 |
| Mean COx | 0.0127 (-0.0287 – 0.0639) | 0.0178 (0 – 0.112) | 0.149 (0.00794 – 0.17) | 1987 | **0.018** | **0.038** |
| % Time COx > 0.20 | 23.5 (16.6 – 30) | 22.9 (16.1 – 32.7) | 41.3 (21 – 41.8) | 1751 | 0.260 | 0.385 |
| Mean COx_a | 0.0577 (0.0145 – 0.11) | 0.0861 (0.0111 – 0.121) | 0.102 (0.00529 – 0.166) | 1410 | 0.326 | 0.435 |
| % Time COx_a > 0.20 | 28.3 (22.2 – 36.8) | 29.8 (21.1 – 34.9) | 29 (19.9 – 42.3) | 1273 | 0.828 | 0.828 |
| Mean PbtO2 (mmHg) | 23.1 (15.4 – 31.5) | 24.5 (16.9 – 28.7) | 12.2 (10.9 – 16) | 549 | 0.244 | 0.375 |
| % Time PbtO2 < 15 mmHg | 6.56 (1.5 – 66.8) | 7.19 (3.76 – 41) | 72.4 (4.17 – 86.8) | 820 | 0.276 | 0.394 |
| % Time PbtO2 < 20 mmHg | 28.7 (5.62 – 90.1) | 30.2 (18.7 – 78.8) | 97.8 (89.4 – 99.5) | 910 | 0.072 | 0.131 |

*Both unadjusted and adjusted p values are presented. Adjusted p values were calculated using the False Discovery Rate (FDR) method. Bolded p values are those reaching statistical significance, p < 0.05.*

*ABP = arterial blood pressure, AMP = pulse amplitude of ICP, COx = cerebral oxygenation index (correlation between rSO2 and CPP), COx_a = cerebral oxygenation index (correlation between rSO2 and ABP), CPP = cerebral perfusion pressure, CT = computed tomography, GCS = Glasgow Coma Scale, ICP = intracranial pressure, IQR = interquartile range, J-T = Jonckheere-Terpstra test, MAP = mean arterial pressure, PAx = pulse amplitude index (correlation between AMP and MAP), PbtO2 = brain tissue oxygen tension, PRx = pressure reactivity index (correlation between ICP and MAP), RAC = correlation (R) between slow waves of AMP (A) and CPP (C), RAP = compensatory reserve index (correlation between AMP and ICP), rSO2 = regional cerebral oxygen saturation.*

Supplemental Appendix H. Jonckheere-Terpstra Testing for PbtO2 Trichotomization

| **Variable** | **PbtO2 < 20 mmHg**  **[n = 44]** | **20 mmHg ≤ PbtO2 ≤ 30 mmHg**  **[n = 39]** | **PbtO2 > 30 mmHg**  **[n = 33]** | **J-T Statistic** | **p-value** | **Adjusted p-value** |
| --- | --- | --- | --- | --- | --- | --- |
| Age (years) | 39 (27 – 50.5) | 48 (26.2 – 57.8) | 36 (24 – 50) | 2122 | 0.850 | 0.850 |
| Sex (% Male) | 88.37% | 79.49% | 78.79% | 2045 | 0.252 | 0.360 |
| Admission GCS | 5 (3 – 6.25) | 3 (3 – 6) | 3 (3 – 6) | 990 | **0.048** | 0.113 |
| Admission GCS – Motor | 2 (1 – 4) | 1 (1 – 4) | 1 (1 – 4) | 959 | 0.558 | 0.638 |
| Admission Pupil Response  (% Bilaterally Reactive) | 16.28% | 12.82% | 9.09% | 2479 | **0.030** | 0.100 |
| Marshall CT Grade | 3 (2 – 4) | 3 (2 – 3) | 3 (2 – 3) | 1523 | 0.180 | 0.277 |
| GOS | 3 (1 – 4) | 3 (1 – 4) | 4 (3 – 5) | 1803 | 0.102 | 0.196 |
| Number with Hypoxic Episode | 46.15% | 30% | 30.43% | 466 | 0.444 | 0.555 |
| Number with Hypotensive Episode | 30.77% | 35% | 39.13% | 537 | 0.662 | 0.716 |
| Duration of Recording (hours) | 155 (92.2 – 231) | 146 (88.3 – 197) | 123 (69 – 276) | 2145 | 0.682 | 0.718 |
| Mean MAP (mmHg) | 87.6 (82.6 – 94.9) | 96.1 (91.7 – 99.9) | 93.4 (90.9 – 98.5) | 2805 | **0.006** | **0.034** |
| Mean ICP (mmHg) | 14.8 (8.25 – 18.7) | 14.5 (9.5 – 17.9) | 13.3 (11.3 – 15.6) | 2165 | 0.710 | 0.728 |
| % Time ICP > 20 mmHg | 14.6 (2.12 – 37.7) | 12.3 (2.53 – 29.2) | 7.26 (3.32 – 20.9) | 2056 | 0.406 | 0.541 |
| % Time ICP > 22 mmHg | 9.7 (1.33 – 25.9) | 7.69 (1.68 – 18) | 4.63 (1.94 – 12.6) | 2091 | 0.484 | 0.569 |
| Mean CPP (mmHg) | 73.1 (70.2 – 81.5) | 81.4 (77.1 – 88.7) | 80 (77 – 82.7) | 2842 | **0.004** | **0.027** |
| % Time CPP < 60 mmHg | 4.92 (1.7 – 13.2) | 2.07 (0.337 – 4.89) | 1.32 (0.491 – 4.93) | 1590 | **0.002** | **0.016** |
| % Time CPP > 70 mmHg | 66.3 (50.9 – 88.2) | 89.8 (74.6 – 96.4) | 84.1 (76.1 – 96.7) | 2952 | **0.002** | **0.016** |
| Mean PRx | 0.124 (0.0331 – 0.244) | 0.11 (-0.0426 – 0.188) | 0.0955 (-0.0164 – 0.151) | 1936 | 0.140 | 0.244 |
| % Time PRx > 0 | 65.4 (52.9 – 77) | 57.9 (44 – 68.5) | 59.4 (47.4 – 64.5) | 1890 | 0.090 | 0.196 |
| % Time PRx > 0.25 | 38.8 (29.9 – 53.8) | 37.4 (22.8 – 45) | 35.7 (21.7 – 42.8) | 1897 | 0.104 | 0.196 |
| % Time PRx > 0.35 | 28.6 (22 – 40.8) | 26.2 (16.9 – 35.2) | 27.5 (14 – 35.8) | 1934 | 0.152 | 0.253 |
| % Time ICP > 20 mmHg & PRx > 0.35 | 5.73 (1.16 – 13.2) | 4.49 (1.07 – 7.1) | 2.98 (1.52 – 8.72) | 2048 | 0.348 | 0.480 |
| Mean PAx | 0.019 (-0.0501 – 0.116) | -0.0763 (-0.13 – 0.0636) | -0.0537 (-0.153 – 0.0583) | 1806 | **0.038** | 0.101 |
| % Time PAx > 0 | 52.3 (43.2 – 64.9) | 41.8 (34.5 – 58.8) | 44.4 (31.4 – 60.5) | 1782 | **0.026** | 0.095 |
| % Time PAx > 0.2 | 29.2 (24.6 – 43.1) | 21.3 (16.2 – 35.5) | 22.5 (14.6 – 36.8) | 1767 | **0.020** | 0.089 |
| % Time PAx > 0.25 | 25.1 (20.4 – 38.9) | 17 (13.4 – 31.1) | 19.2 (12.8 – 30.6) | 1768 | **0.024** | 0.095 |
| % Time ICP > 20 mmHg & PAx > 0.25 | 4.62 (0.967 – 9.65) | 3.64 (0.922 – 7.13) | 2.22 (0.871 – 5.15) | 1915 | 0.108 | 0.196 |
| Mean RAC | -0.278 (-0.407 – -0.11) | -0.342 (-0.506 – -0.191) | -0.384 (-0.55 – -0.234) | 1793 | **0.034** | 0.101 |
| % Time RAC > 0 | 22.8 (15.2 – 37.4) | 17.1 (9.91 – 30.7) | 16.3 (7.76 – 28.2) | 1848 | **0.048** | 0.113 |
| % Time ICP > 20 mmHg & RAC > 0 | 2.37 (0.46 – 7.27) | 1.52 (0.602 – 5.71) | 1.19 (0.239 – 2.95) | 1952 | 0.176 | 0.277 |
| Mean RAP | 0.665 (0.535 – 0.779) | 0.656 (0.521 – 0.799) | 0.705 (0.528 – 0.788) | 2321 | 0.626 | 0.696 |
| % Time RAP > 0.40 | 80.9 (70.7 – 90) | 81.3 (70.9 – 91) | 84.9 (72.1 – 90.8) | 2376 | 0.442 | 0.555 |
| rSO_2_ | 69.9 (64.1 – 72.2) | 73.8 (69.7 – 84.4) | 68.1 (66.7 – 73.1) | 3.12 | 0.480 | 0.569 |
| Mean COx | 0.0102 (-0.0196 – 0.074) | 0.00195 (0 – 0.0258) | -0.008 (-0.0615 – 0) | 4.51 | **0.036** | 0.101 |
| % Time COx > 0.20 | 24.6 (16.4 – 30.9) | 14.1 (0 – 23) | 16 (11.7 – 19.9) | 7.75 | **0.020** | 0.089 |
| Mean COx_a | 0.0491 (0.00797 – 0.0903) | 0.0728 (0.0532 – 0.0998) | 0.097 (0.0839 – 0.111) | 1.54 | 0.216 | 0.320 |
| % Time COx_a > 0.20 | 26.7 (19.1 – 30.7) | 26.7 (23.7 – 33.3) | 39.9 (37.9 – 40) | 3.91 | 0.096 | 0.196 |
| Mean PbtO2 (mmHg) | 12.6 (10.1 – 15.6) | 24.9 (22.8 – 27.4) | 34 (32.1 – 38.2) | 4455 | **0.002** | **0.016** |
| % Time PbtO_2_ < 15 mmHg | 86.3 (61 – 95) | 4.59 (1.59 – 11.3) | 0.885 (0.157 – 1.77) | 295 | **0.002** | **0.016** |
| % Time PbtO_2_ < 20 mmHg | 96.6 (88.9 – 98.9) | 24.4 (12.7 – 33.3) | 2.88 (0.515 – 6.56) | 118 | **0.002** | **0.016** |

*Both unadjusted and adjusted p values are presented. Adjusted p values were calculated using the False Discovery Rate (FDR) method. Bolded p values are those reaching statistical significance, p < 0.05.*

*AMP = pulse amplitude of ICP, COx = cerebral oxygenation index (correlation between rSO_2_ and CPP), COx_a = cerebral oxygenation index (correlation between rSO_2_ and ABP), CPP = cerebral perfusion pressure, CT = computed tomography, GCS = Glasgow Coma Scale, ICP = intracranial pressure, IQR = interquartile range, J-T = Jonckheere-Terpstra test, MAP = mean arterial pressure, PAx = pulse amplitude index (correlation between AMP and MAP), PbtO_2_ = brain tissue oxygen tension, PRx = pressure reactivity index (correlation between ICP and MAP), RAC = correlation (R) between slow waves of AMP (A) and CPP (C), RAP = compensatory reserve index (correlation between AMP and ICP), rSO_2_ = regional cerebral oxygen saturation.*

Supplemental Appendix I. Jonckheere-Terpstra Testing for rSO_2_ Trichotomization

| **Variable** | **rSO2 < 60 mmHg**  **[n = 19]** | **60 mmHg ≤ rSO2 ≤ 70 mmHg**  **[n = 58]** | **rSO2 > 70 mmHg**  **[n = 69]** | **J-T Statistic** | **p-value** | **Adjusted p-value** |
| --- | --- | --- | --- | --- | --- | --- |
| Age (years) | 42 (30.5 – 58) | 39 (28 – 50.8) | 40 (27.5 – 57) | 3152 | 0.186 | 0.591 |
| Sex (% Male) | 78.95% | 72.41% | 88.24% | 3505 | **0.040** | 0.250 |
| Admission GCS | 5 (4 – 6) | 7 (5 – 8.75) | 5 (3 – 7) | 2677 | **0.046** | 0.250 |
| Admission GCS – Motor | 3 (2 – 4.5) | 5 (3 – 5) | 4 (1 – 5) | 1896 | **0.006** | 0.120 |
| Admission Pupil Response  (% Bilaterally Reactive) | 15.79% | 10.34% | 17.65% | 3003 | 0.192 | 0.591 |
| Marshall CT Grade | 4 (3 – 5) | 4 (3 – 5) | 4 (3 – 5) | 2885 | 0.690 | 0.863 |
| GOS | 4 (1 – 5) | 4 (1 – 5) | 3 (1 – 5) | 2798 | 0.336 | 0.616 |
| Number with Hypoxic Episode | 29.41% | 22.92% | 30.51% | 2412 | 0.780 | 0.868 |
| Number with Hypotensive Episode | 0% | 8.33% | 10.34% | 2406 | 0.152 | 0.591 |
| Duration of Recording (hours) | 79.2 (33 – 124) | 115 (57.3 – 160) | 71.2 (40.4 – 132) | 2893 | 0.150 | 0.591 |
| Mean MAP (mmHg) | 82.6 (78.6 – 88.2) | 82.5 (78.8 – 88.7) | 85.1 (79 – 89.4) | 3504 | 0.314 | 0.616 |
| Mean ICP (mmHg) | 9.64 (4.26 – 11.9) | 10.4 (6.19 – 13) | 9.91 (7.62 – 14.4) | 3503 | 0.296 | 0.616 |
| % Time ICP > 20 mmHg | 1.66 (0.237 – 5.69) | 2.06 (0.0964 – 8.18) | 1.66 (0.159 – 9.06) | 3300 | 0.952 | 0.952 |
| % Time ICP > 22 mmHg | 0.985 (0.0576 – 3.74) | 1.28 (0.0417 – 4.99) | 0.767 (0 – 4.22) | 3191 | 0.758 | 0.868 |
| Mean CPP (mmHg) | 73.4 (70 – 78.9) | 73.4 (70.1 – 79.4) | 73.6 (69.9 – 78.1) | 3183 | 0.846 | 0.868 |
| % Time CPP < 60 mmHg | 5.52 (2.35 – 10) | 6.57 (1.92 – 11.8) | 4.65 (1.45 – 10.3) | 2996 | 0.416 | 0.616 |
| % Time CPP > 70 mmHg | 64.4 (47.1 – 74.1) | 62.7 (43.8 – 76.1) | 61.7 (44 – 76.8) | 3198 | 0.798 | 0.868 |
| Mean PRx | 0.145 (0.0729 – 0.242) | 0.0832 (0.00483 – 0.198) | 0.148 (0.0371 – 0.263) | 3372 | 0.456 | 0.651 |
| % Time PRx > 0 | 64.2 (53.1 – 73.2) | 58.1 (49.7 – 72.6) | 65.7 (53.1 – 78.9) | 3455 | 0.404 | 0.616 |
| % Time PRx > 0.25 | 39.5 (33 – 52.9) | 32.8 (25.4 – 45.6) | 41.2 (27.7 – 54.5) | 3398 | 0.380 | 0.616 |
| % Time PRx > 0.35 | 31.2 (26 – 42.4) | 24.5 (18.7 – 37.5) | 31.2 (19.3 – 45.3) | 3340 | 0.398 | 0.616 |
| % Time ICP > 20 mmHg & PRx > 0.35 | 0.999 (0.221 – 2.63) | 1.18 (0.00375 – 3.34) | 0.884 (0.0596 – 4.59) | 3179 | 0.830 | 0.868 |
| Mean PAx | 0.0357 (-0.0863 – 0.25) | -0.0038 (-0.118 – 0.111) | -0.0329 (-0.147 – 0.111) | 2877 | **0.024** | 0.250 |
| % Time PAx > 0 | 54 (37.9 – 71.3) | 48.4 (36.1 – 65.2) | 46.7 (34.5 – 65.1) | 2961 | **0.036** | 0.250 |
| % Time PAx > 0.2 | 34.5 (20.4 – 54.8) | 29.2 (16.6 – 41.9) | 26.1 (15.5 – 42.8) | 2892 | **0.042** | 0.250 |
| % Time PAx > 0.25 | 30 (17.1 – 50.9) | 24.6 (13.6 – 36.1) | 21.4 (12.5 – 38.3) | 2867 | **0.049** | 0.250 |
| % Time ICP > 20 mmHg & PAx > 0.25 | 0.535 (0.183 – 2.17) | 0.803 (0.0204 – 2.45) | 0.487 (0.0404 – 3.26) | 3165 | 0.682 | 0.863 |
| Mean RAC | -0.213 (-0.311 – 0.0333) | -0.265 (-0.41 – -0.102) | -0.275 (-0.433 – -0.0431) | 3060 | 0.210 | 0.600 |
| % Time RAC > 0 | 34.1 (16.6 – 55) | 23.9 (13.5 – 38) | 22 (14.3 – 43.1) | 3038 | 0.238 | 0.616 |
| % Time ICP > 20 mmHg & RAC > 0 | 0.45 (0.0801 – 1.26) | 0.352 (0.0136 – 1.64) | 0.252 (0 – 2.18) | 3192 | 0.816 | 0.868 |
| Mean RAP | 0.599 (0.526 – 0.682) | 0.706 (0.561 – 0.801) | 0.619 (0.483 – 0.748) | 3022 | 0.362 | 0.616 |
| % Time RAP > 0.40 | 77.7 (68.2 – 84.2) | 87.1 (73.3 – 92.8) | 78.8 (62 – 87.9) | 2969 | 0.184 | 0.591 |
| rSO2 | 56.6 (52 – 58.3) | 64.9 (62.5 – 67.2) | 75.4 (72.5 – 78.4) | 5480 | **0.002** | 0.080 |
| Mean COx | 0.0615 (-0.015 – 0.142) | 0.00533 (-0.0559 – 0.0667) | 0.0177 (-0.00304 – 0.0748) | 2810 | 0.788 | 0.868 |
| % Time COx > 0.20 | 28.6 (21.9 – 39.5) | 23.5 (18.6 – 31) | 24.4 (17 – 31.8) | 2513 | 0.376 | 0.616 |
| Mean COx_a | 0.0936 (0.0364 – 0.168) | 0.0528 (0.0154 – 0.102) | 0.0627 (0.0131 – 0.111) | 2506 | 0.642 | 0.863 |
| % Time COx_a > 0.20 | 31.3 (26.3 – 44.5) | 28.1 (22.7 – 37) | 28.6 (20.6 – 37.2) | 2369 | 0.288 | 0.616 |
| Mean PbtO2 (mmHg) | 10.9 (6.85 – 12.4) | 18.7 (10.9 – 32.4) | 15.7 (12.4 – 25.9) | 196 | 0.658 | 0.863 |
| % Time PbtO2 < 15 mmHg | 86.8 (72.2 – 93.4) | 26.1 (7.69 – 93.7) | 40.7 (3.61 – 86) | 160 | 0.354 | 0.616 |
| % Time PbtO2 < 20 mmHg | 99.5 (99.1 – 99.8) | 70.6 (13.4 – 97.8) | 83.9 (22.8 – 98.2) | 162 | 0.384 | 0.616 |

*Both unadjusted and adjusted p values are presented. Adjusted p values were calculated using the False Discovery Rate (FDR) method. Bolded p values are those reaching statistical significance, p < 0.05.*

*ABP = arterial blood pressure, AMP = pulse amplitude of ICP, COx = cerebral oxygenation index (correlation between rSO2 and CPP), COx_a = cerebral oxygenation index (correlation between rSO2 and ABP), CPP = cerebral perfusion pressure, CT = computed tomography, GCS = Glasgow Coma Scale, ICP = intracranial pressure, IQR = interquartile range, J-T = Jonckheere-Terpstra test, MAP = mean arterial pressure, PAx = pulse amplitude index (correlation between AMP and MAP), PbtO2 = brain tissue oxygen tension, PRx = pressure reactivity index (correlation between ICP and MAP), RAC = correlation (R) between slow waves of AMP (A) and CPP (C), RAP = compensatory reserve index (correlation between AMP and ICP), rSO2 = regional cerebral oxygen saturation.*

Supplemental Appendix J. Insult Burden Histograms for ICP Trichotomization


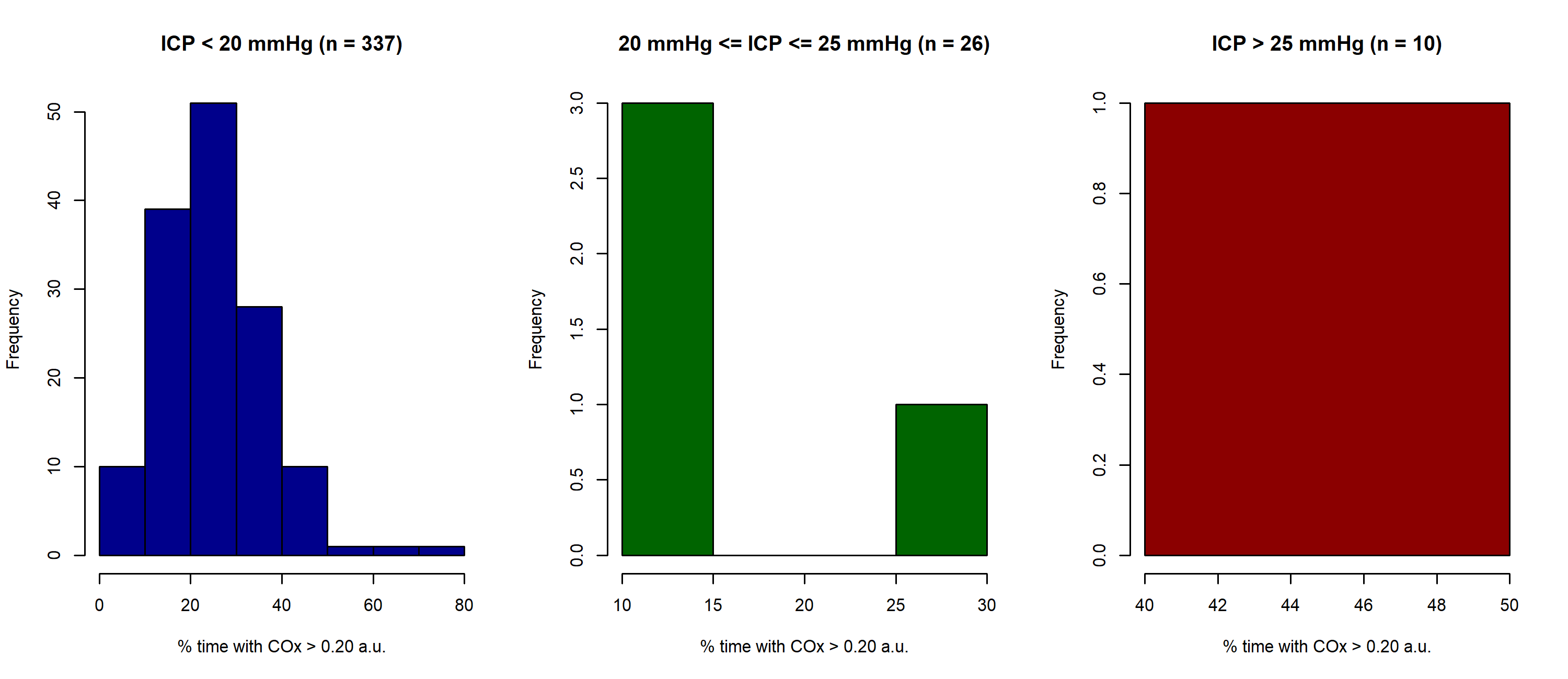

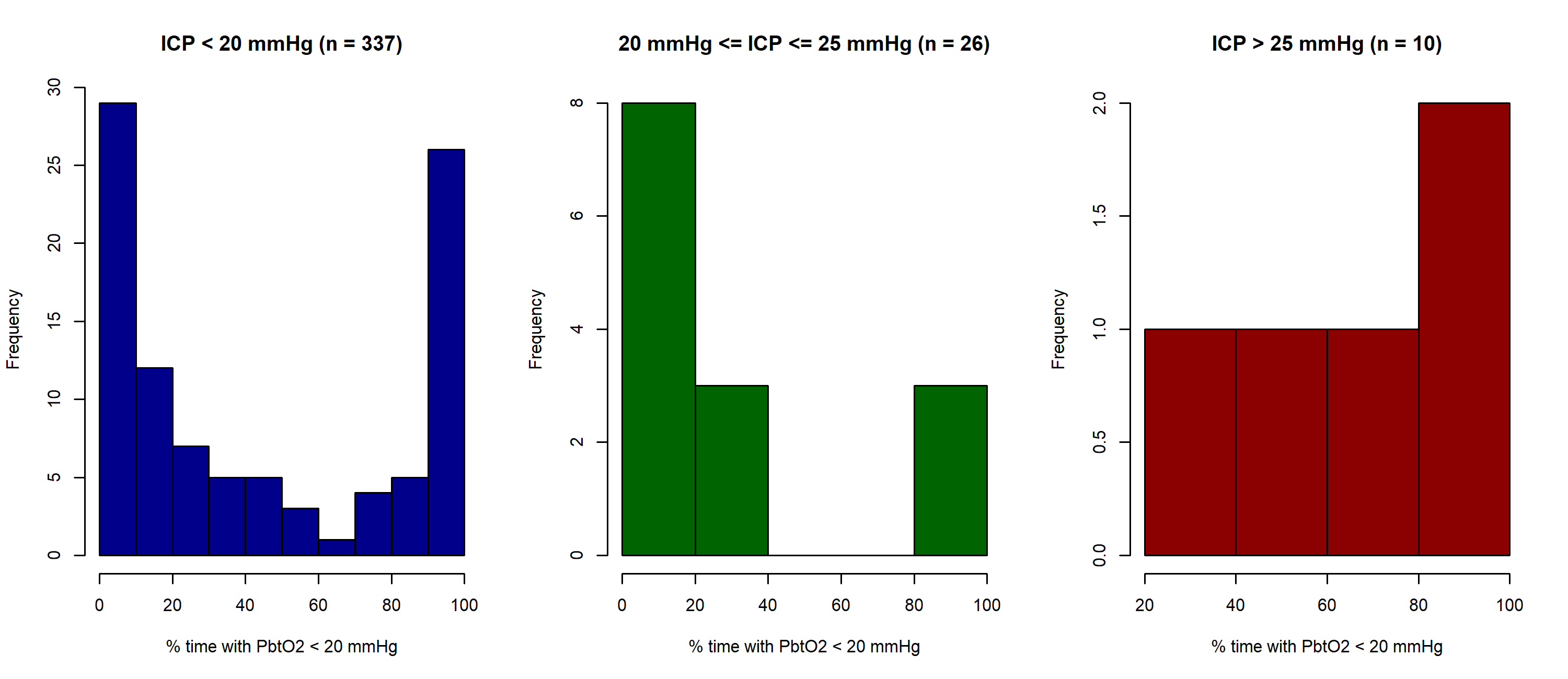

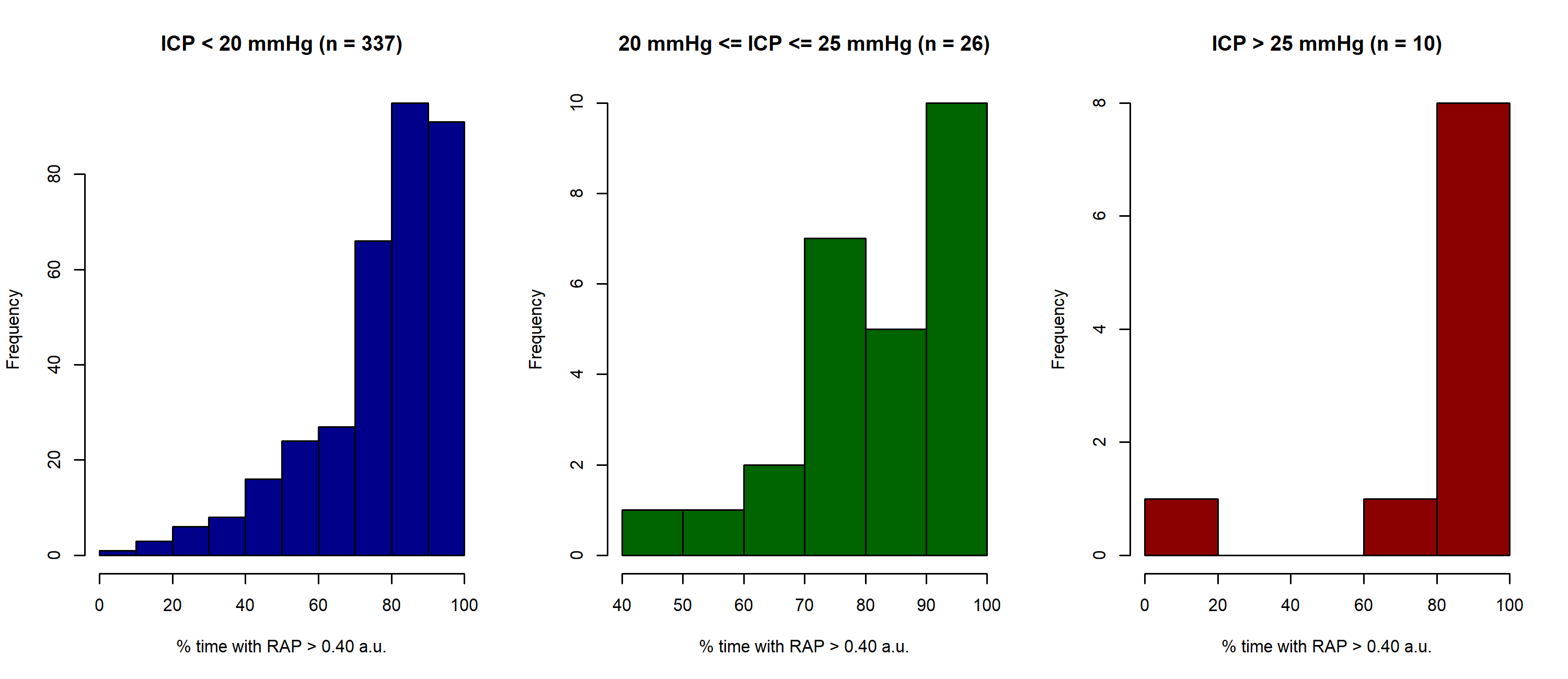

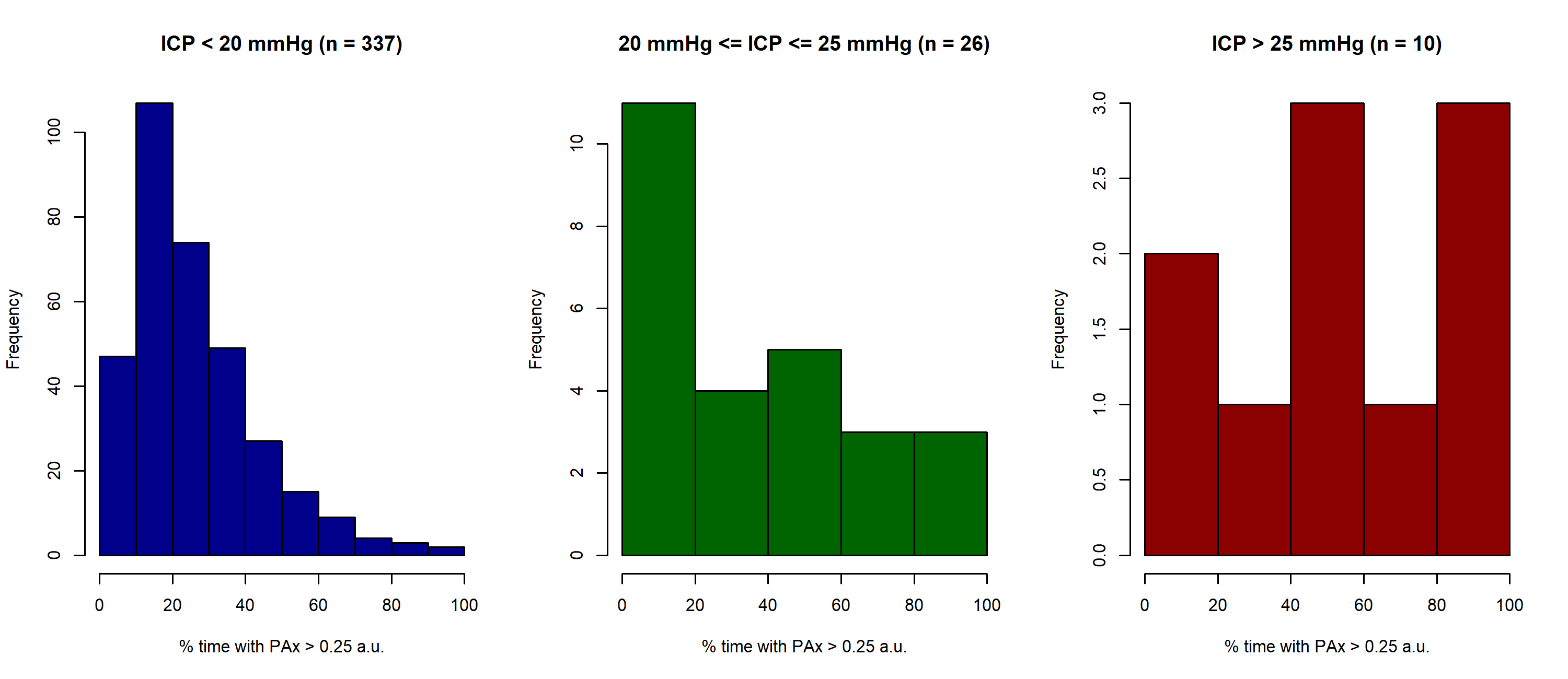

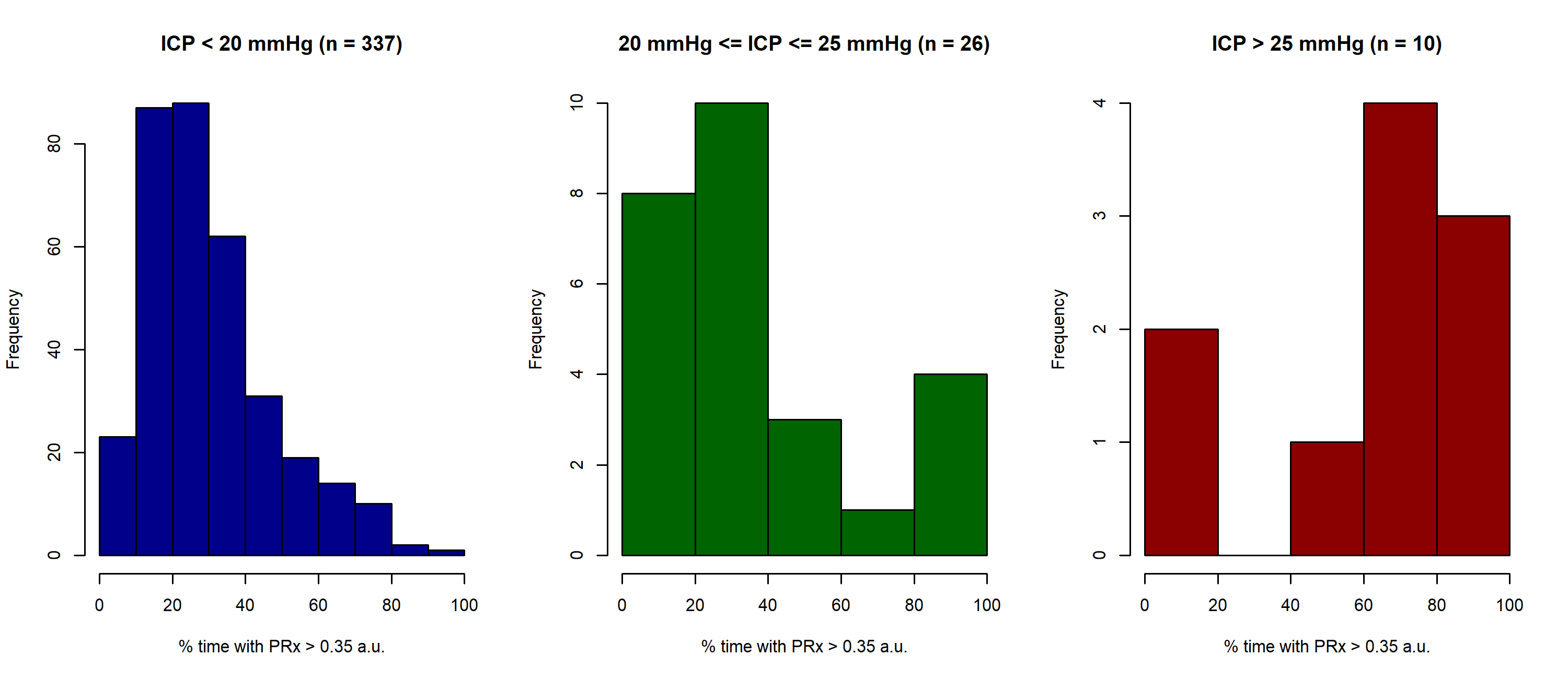

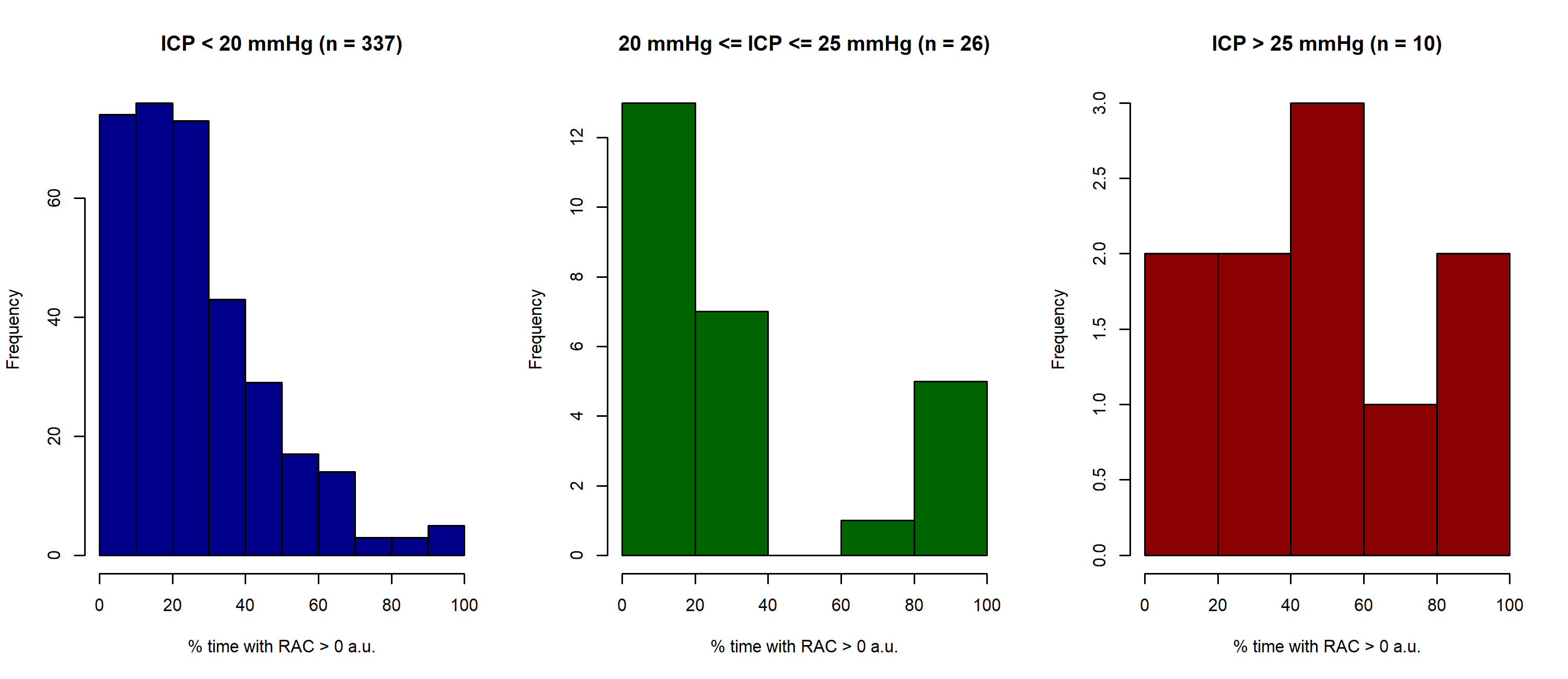

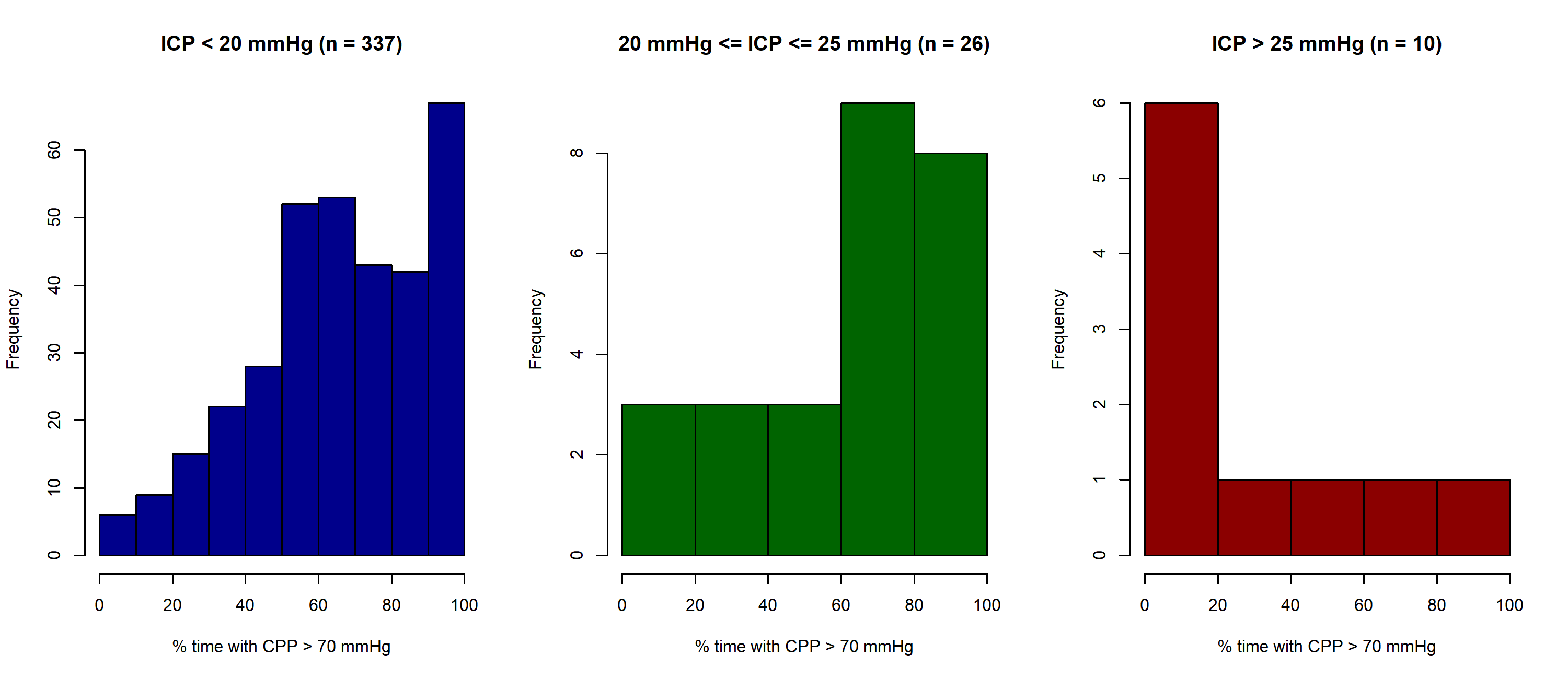

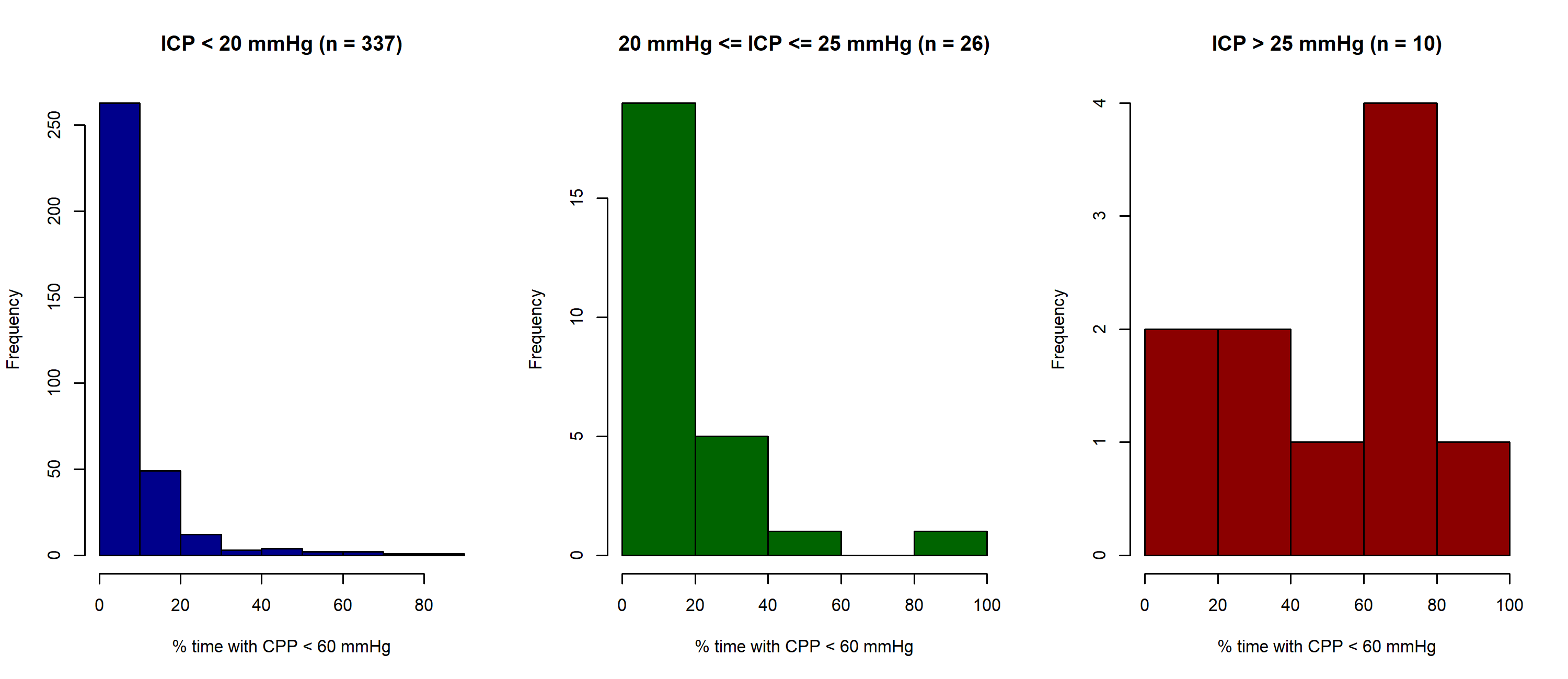

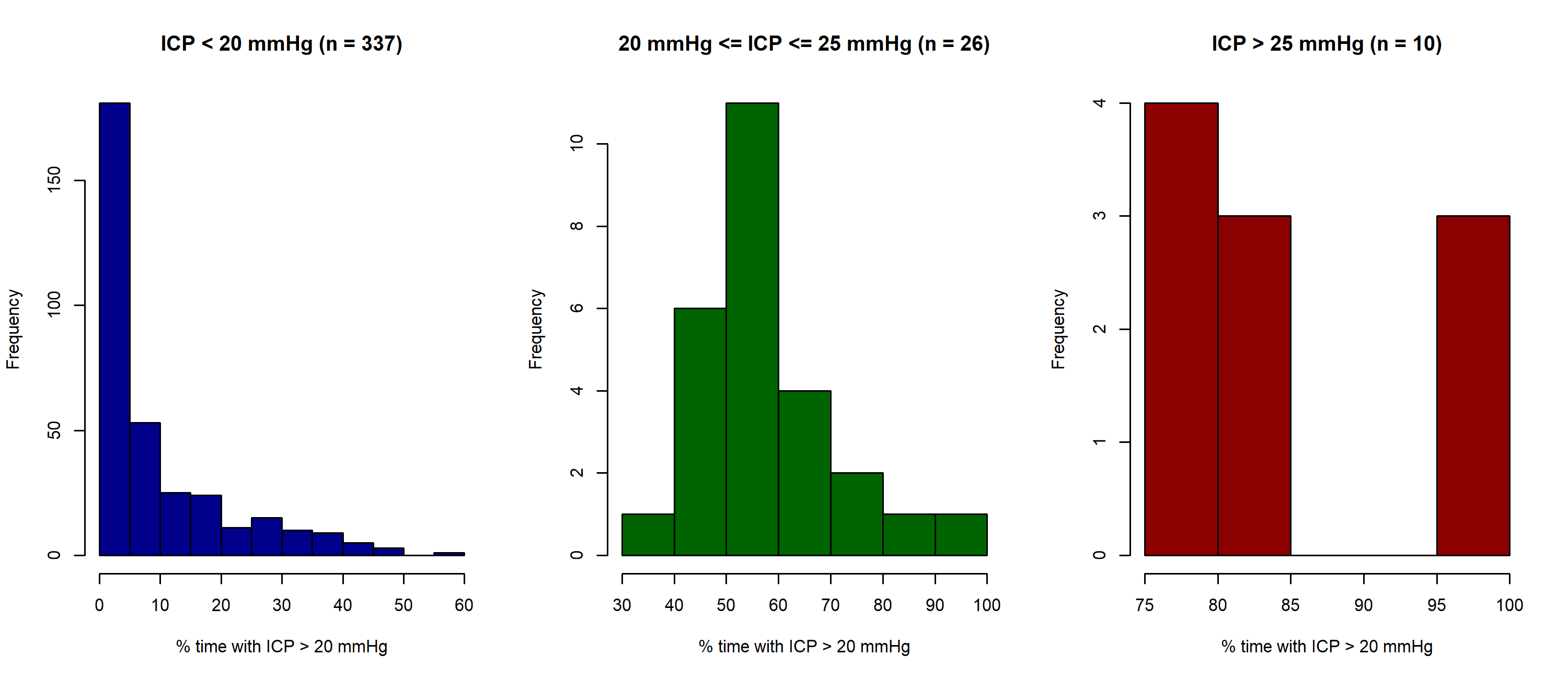


*AMP = pulse amplitude of ICP, COx = cerebral oxygenation index (correlation between rSO2 and CPP), CPP = cerebral perfusion pressure, ICP = intracranial pressure, MAP = mean arterial pressure, PAx = pulse amplitude index (correlation between AMP and MAP), PbtO2 = brain tissue oxygen tension, PRx = pressure reactivity index (correlation between ICP and MAP), RAC = correlation (R) between slow waves of AMP (A) and CPP (C), RAP = compensatory reserve index (correlation between AMP and ICP), rSO2 = regional cerebral oxygen saturation.*

Supplemental Appendix K. Insult Burden Histograms for CPP Trichotomization


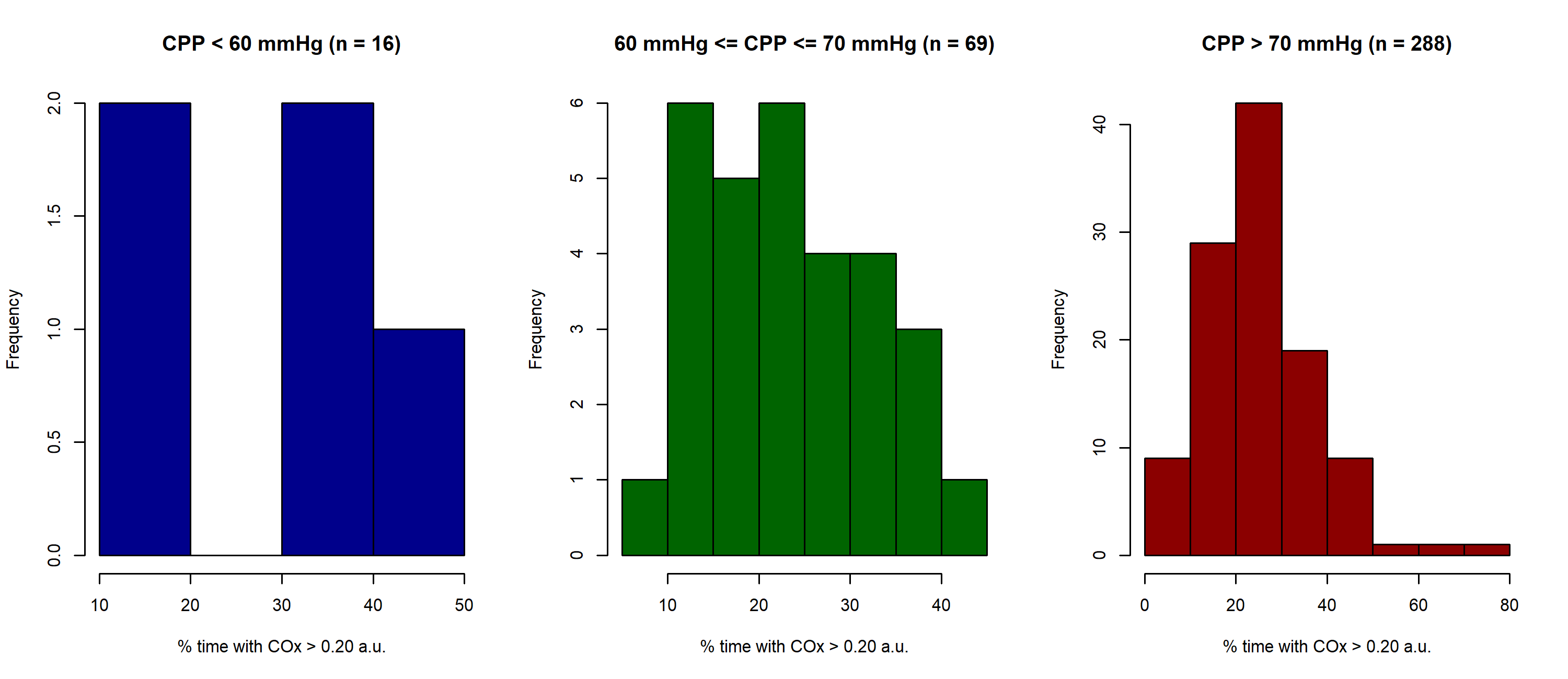

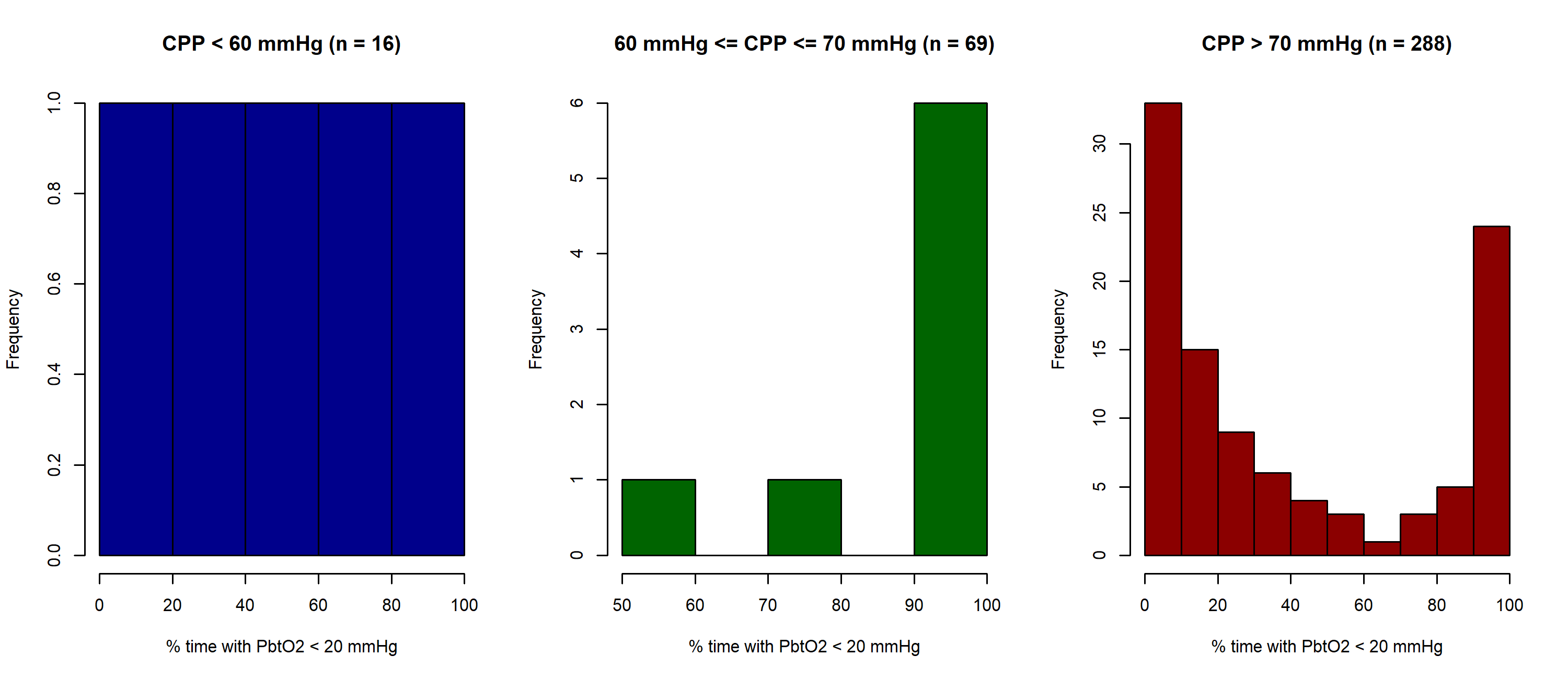

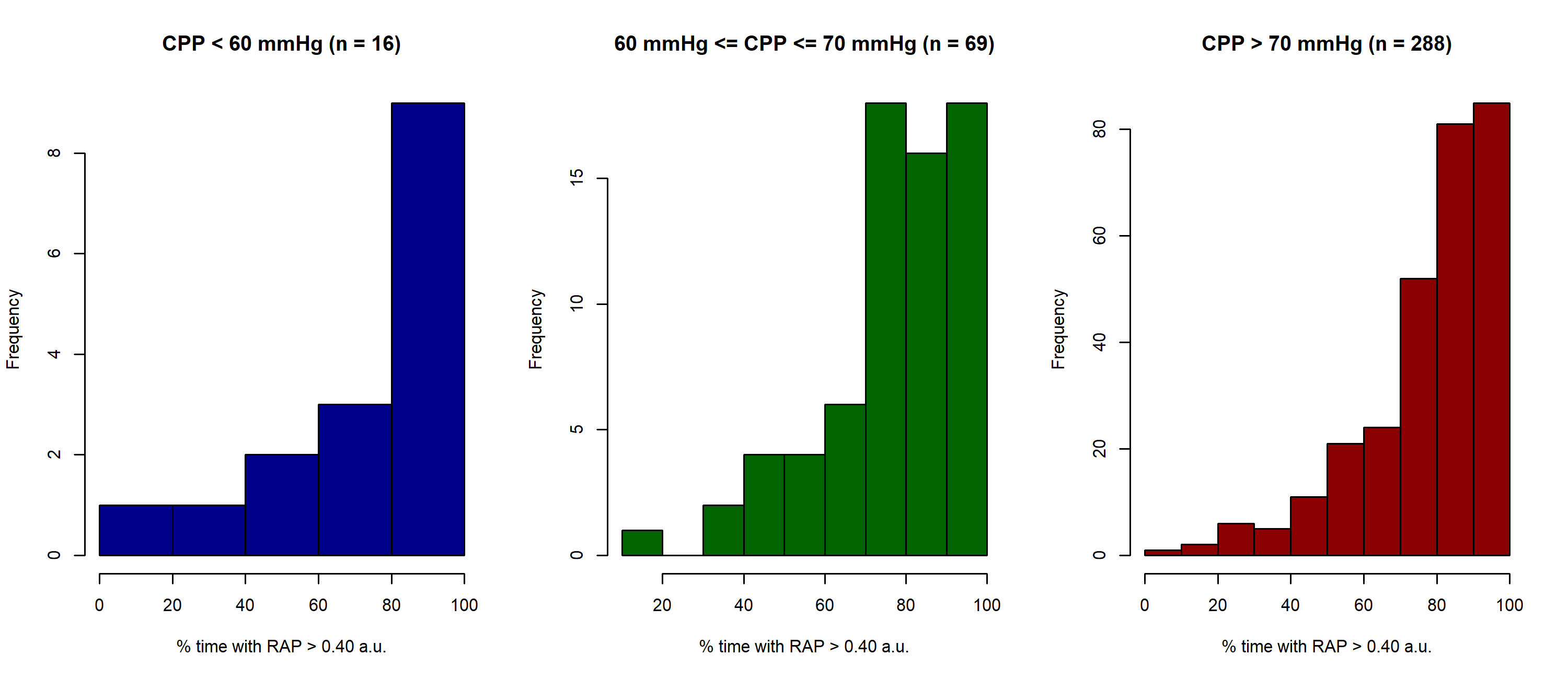

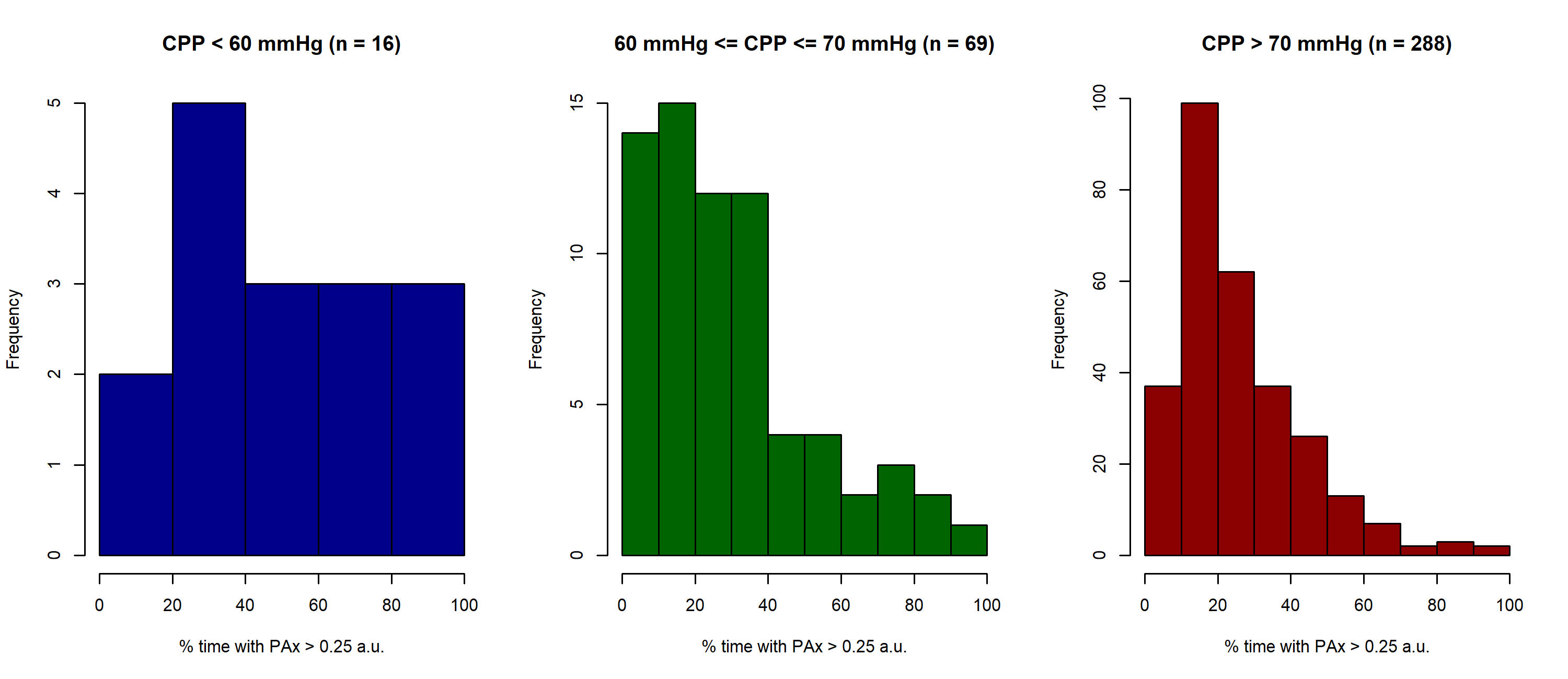

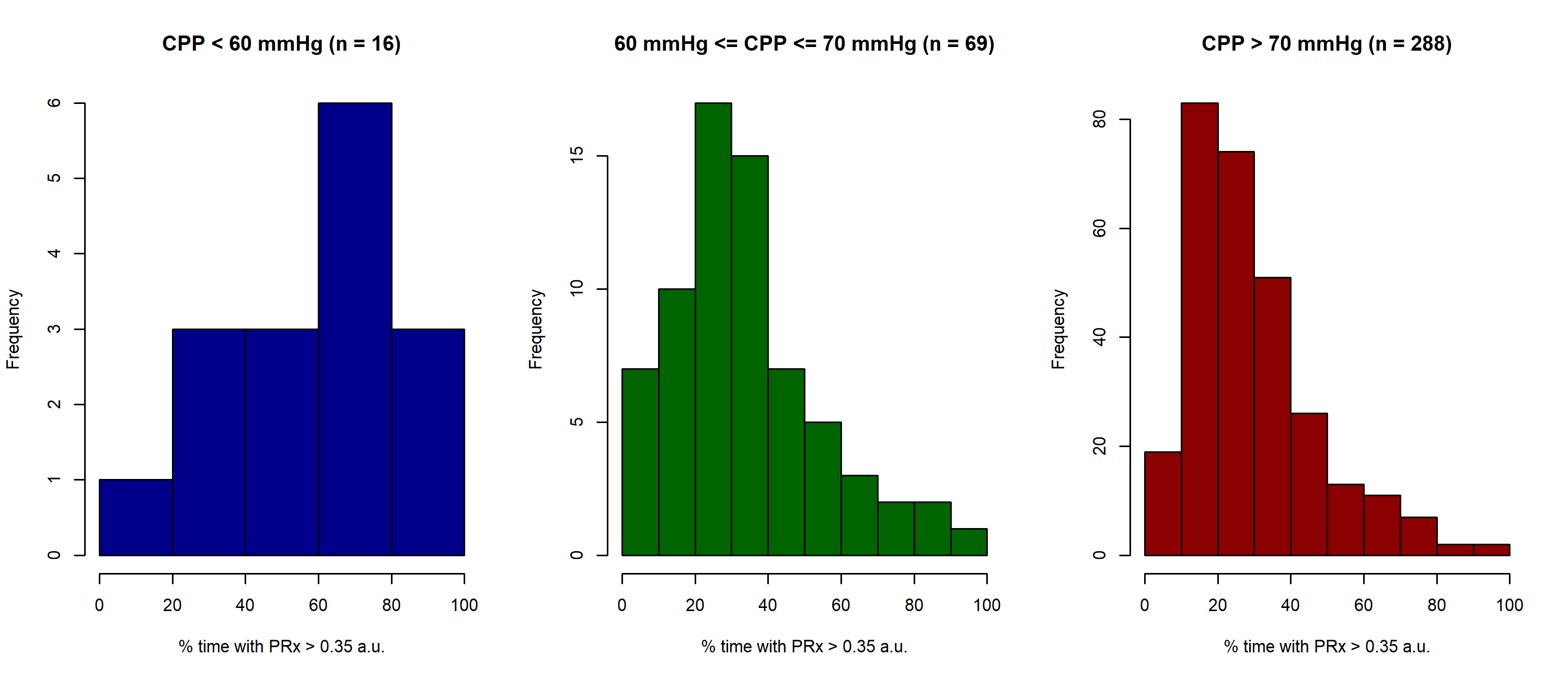

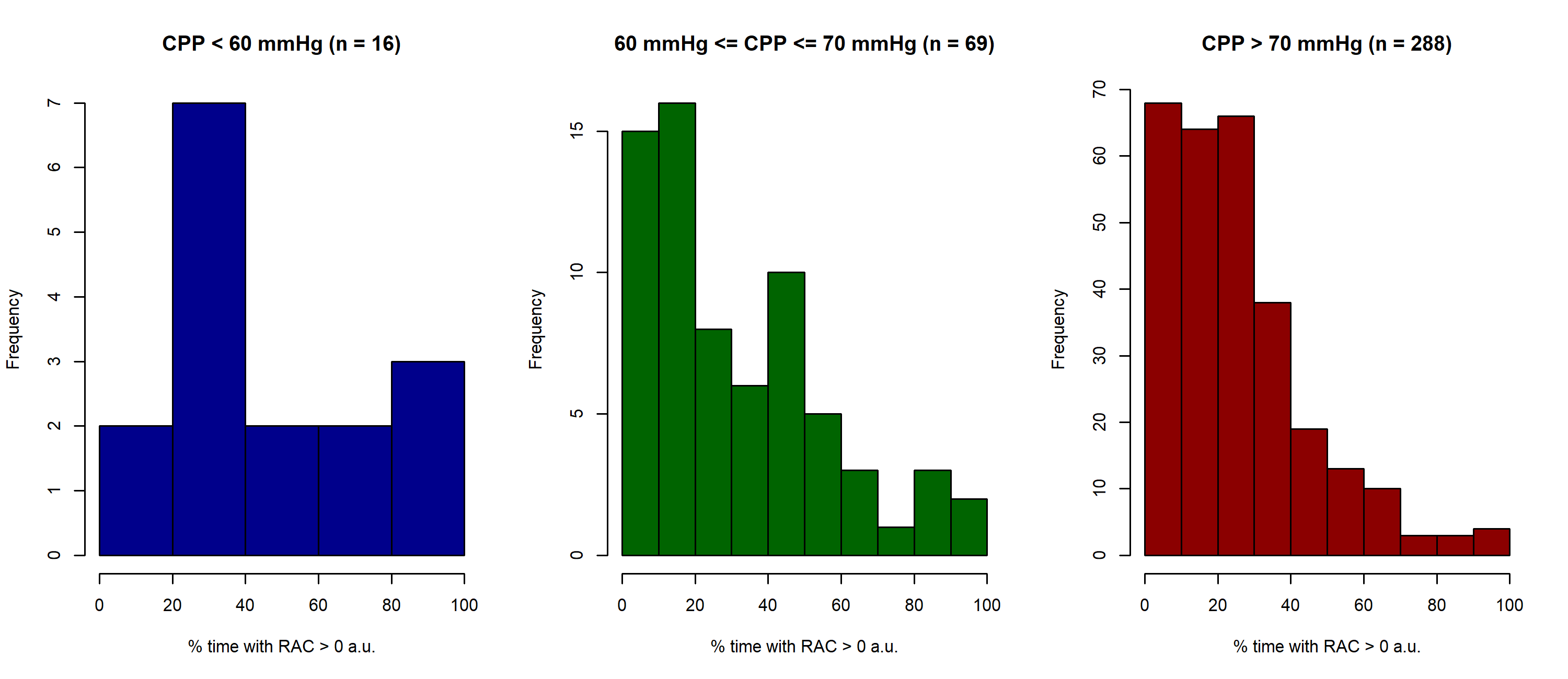

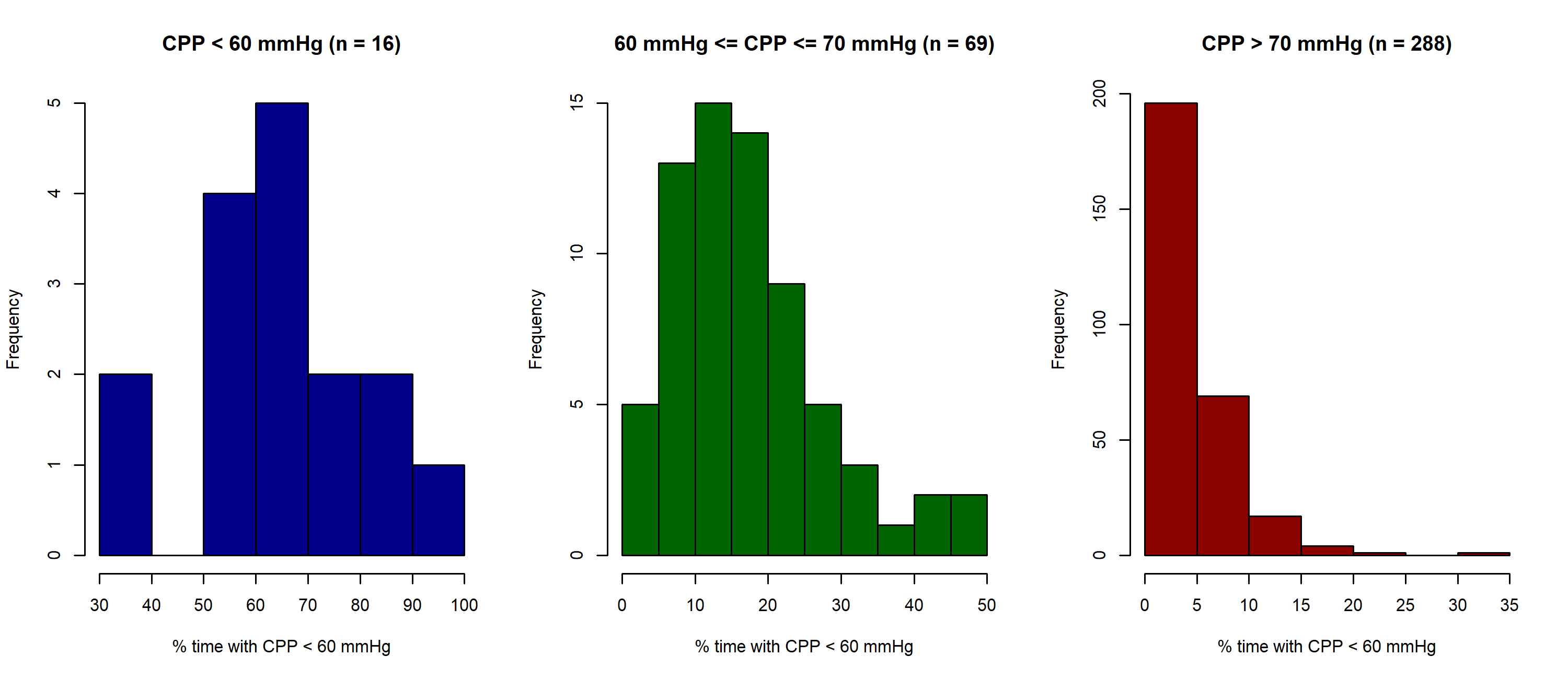

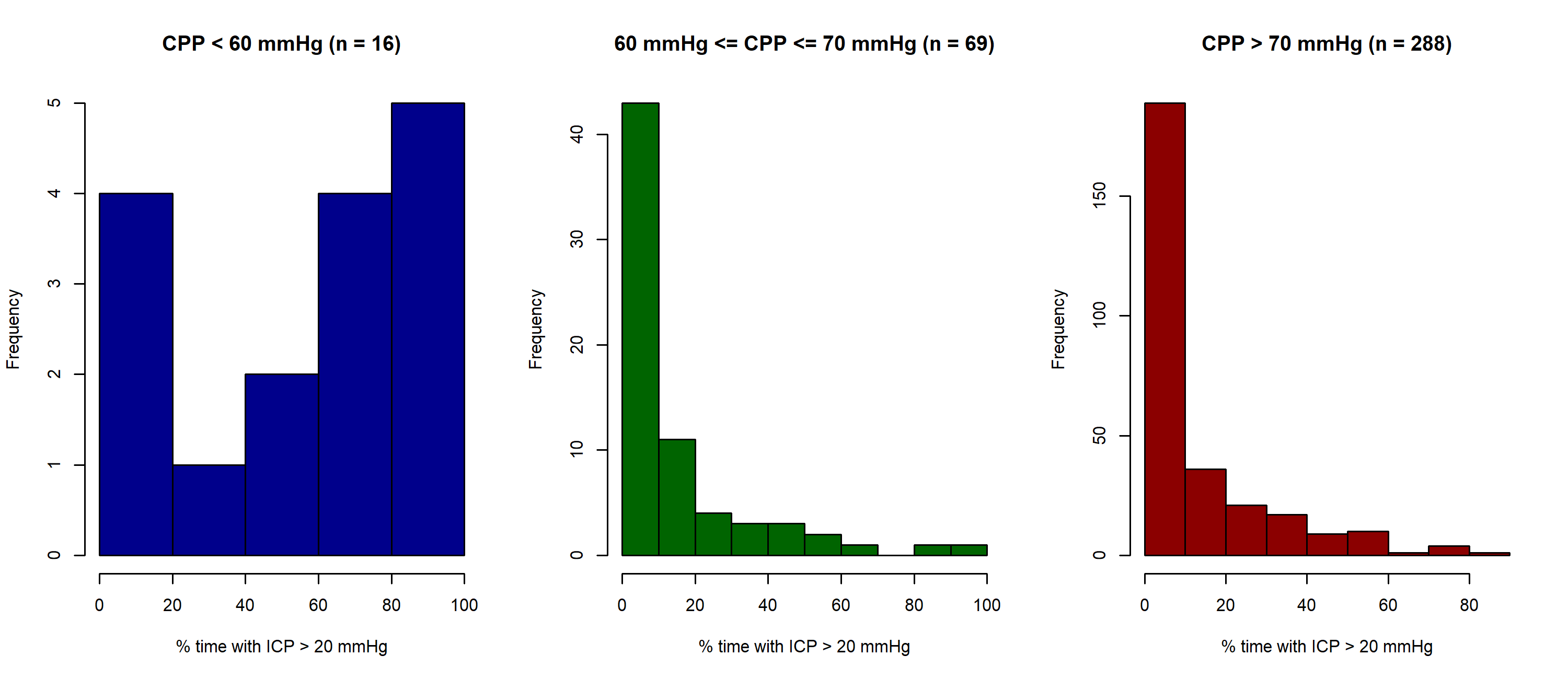

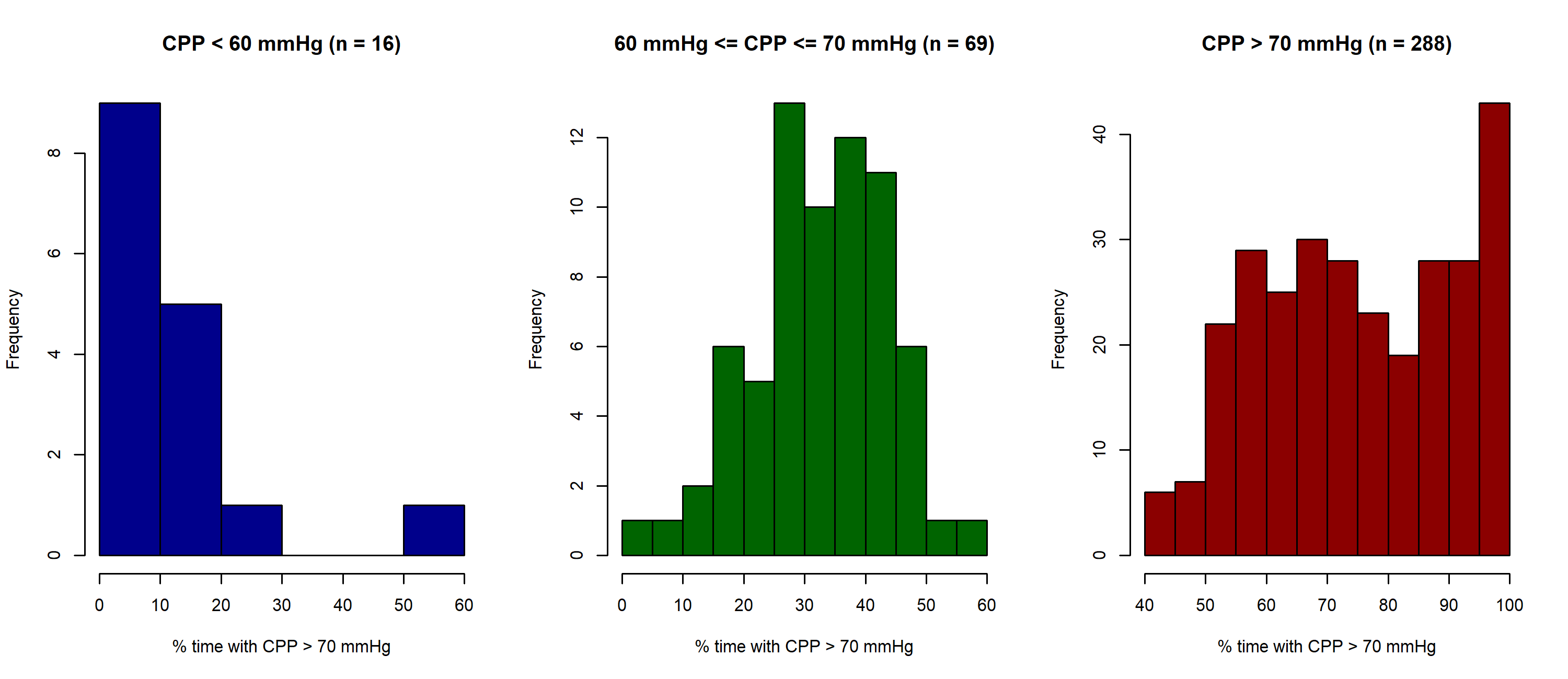


*AMP = pulse amplitude of ICP, COx = cerebral oxygenation index (correlation between rSO2 and CPP), CPP = cerebral perfusion pressure, ICP = intracranial pressure, MAP = mean arterial pressure, PAx = pulse amplitude index (correlation between AMP and MAP), PbtO2 = brain tissue oxygen tension, PRx = pressure reactivity index (correlation between ICP and MAP), RAC = correlation (R) between slow waves of AMP (A) and CPP (C), RAP = compensatory reserve index (correlation between AMP and ICP), rSO2 = regional cerebral oxygen saturation.*

Supplemental Appendix L. Insult Burden Histograms for PRx Trichotomization


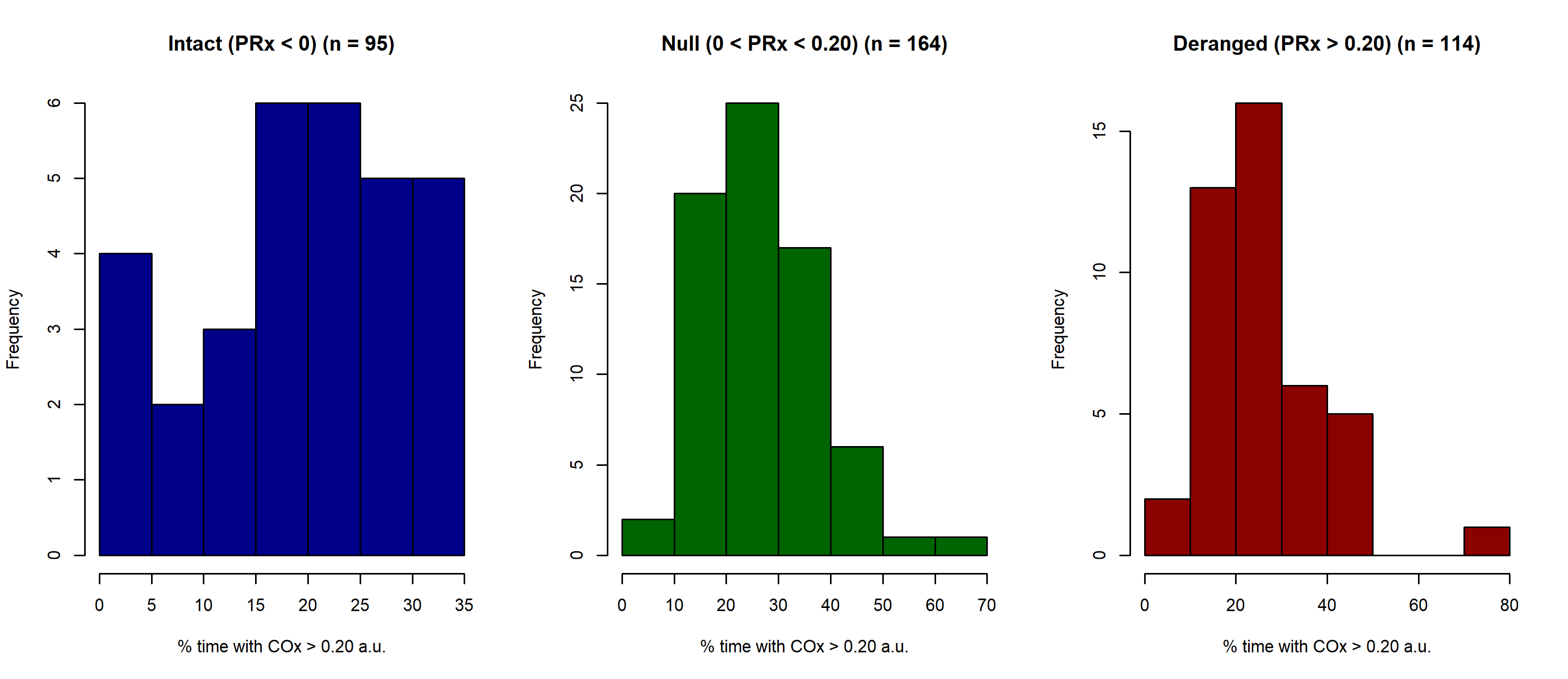

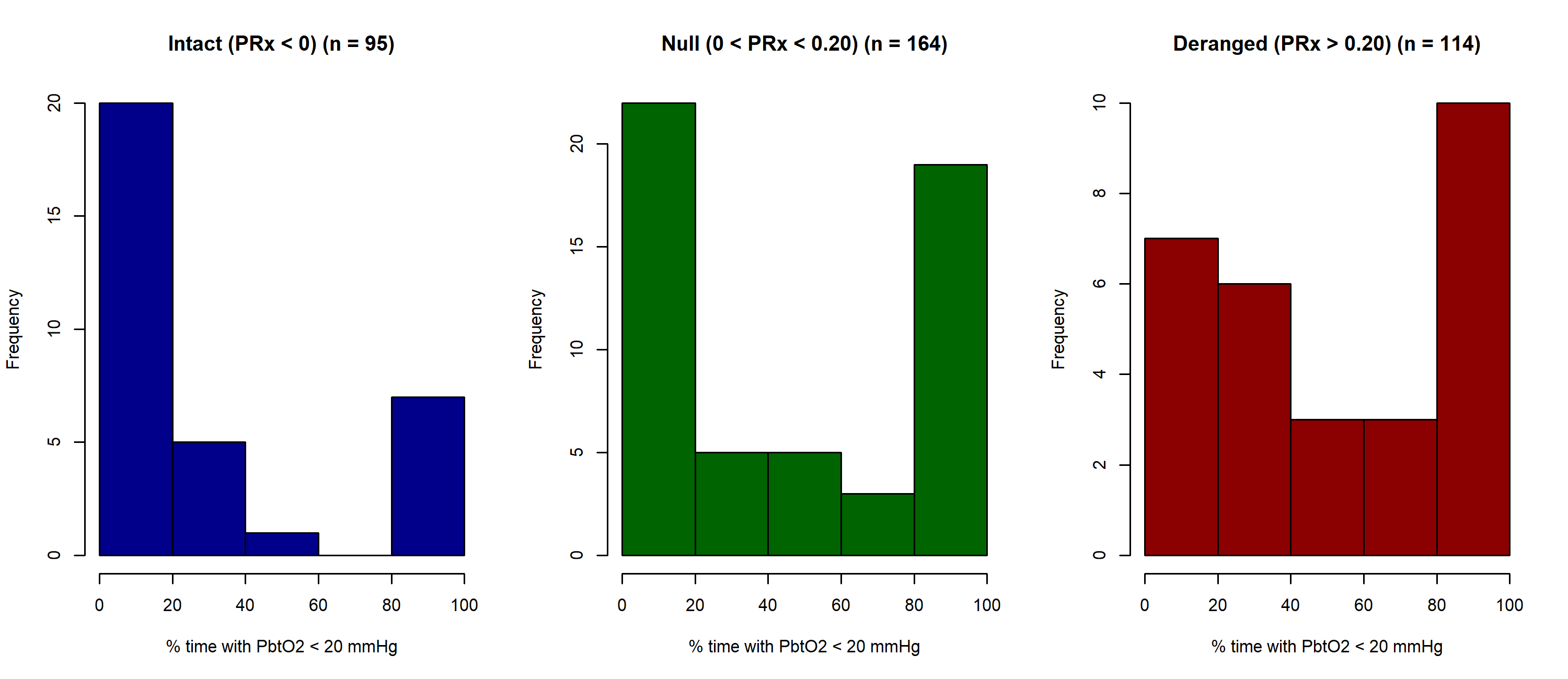

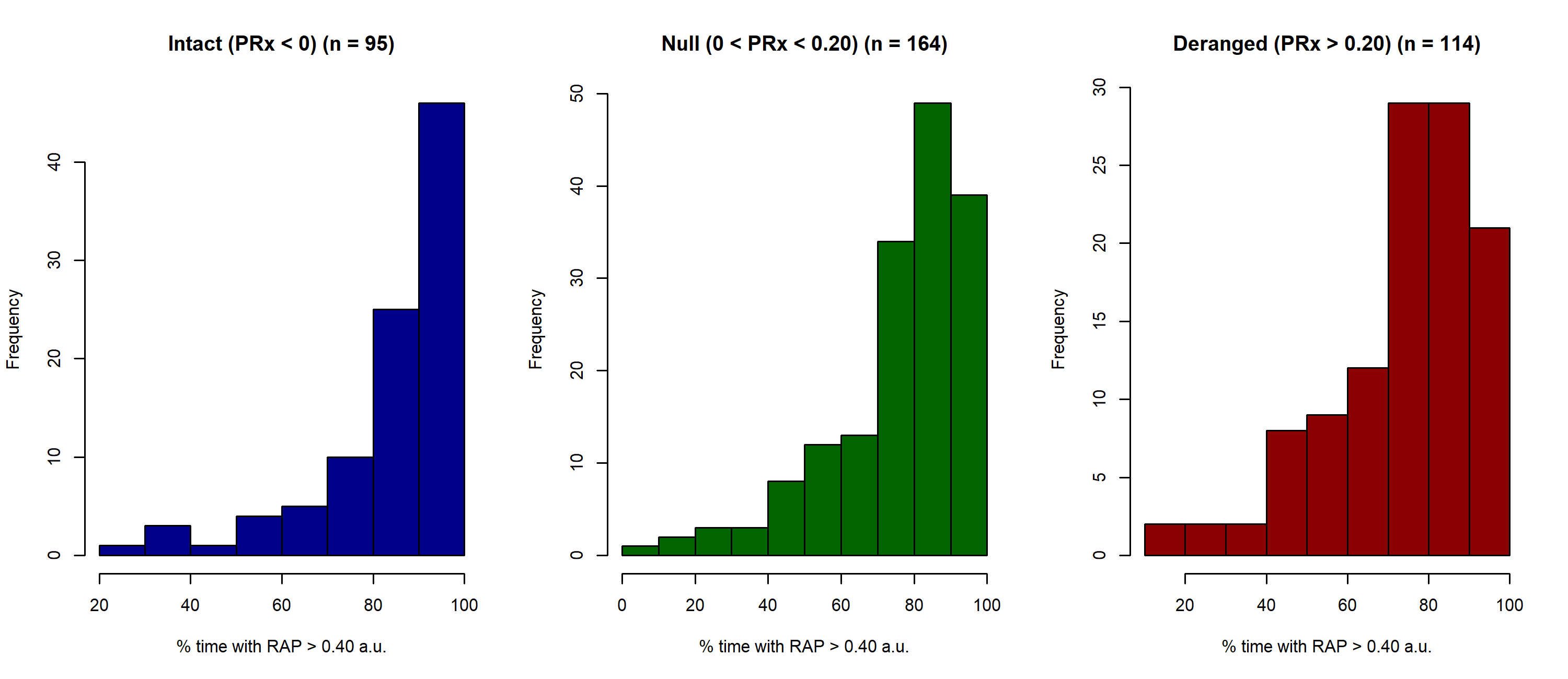

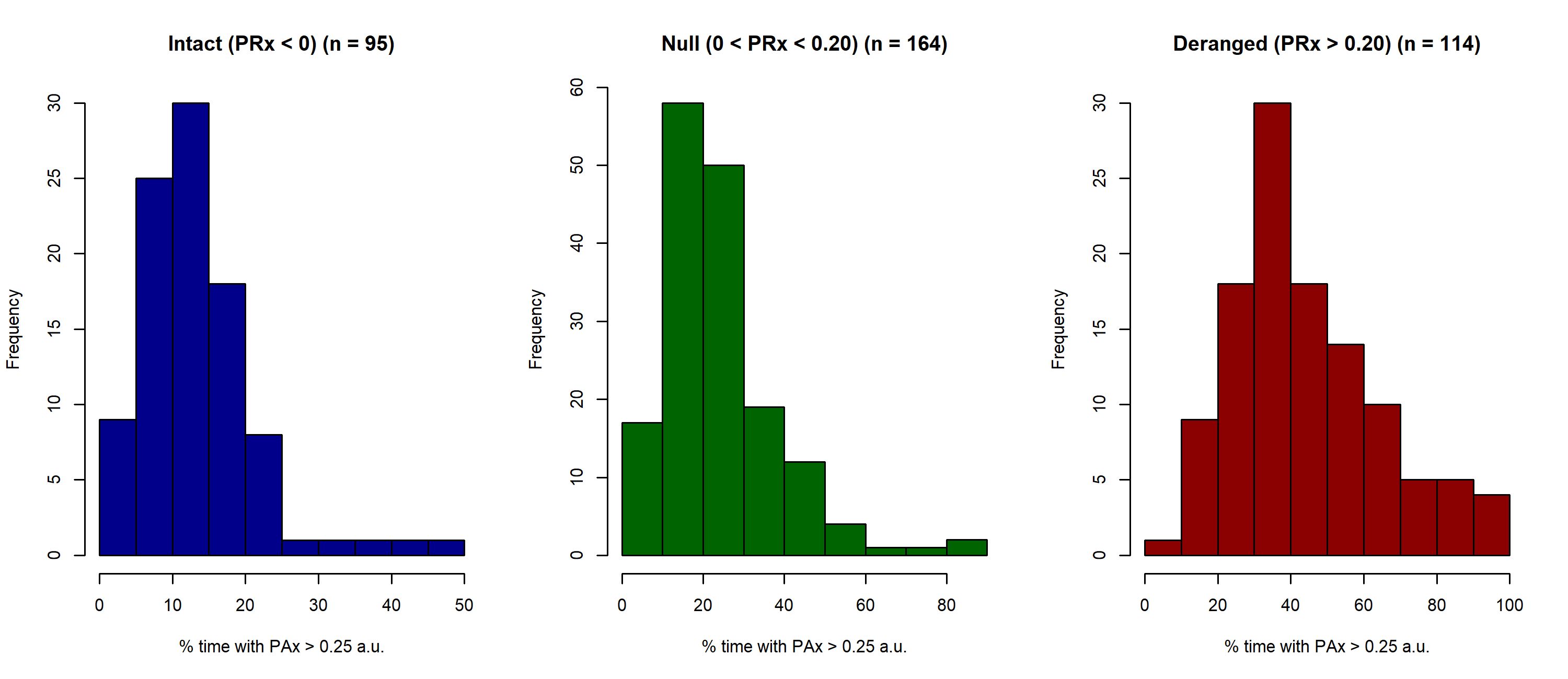

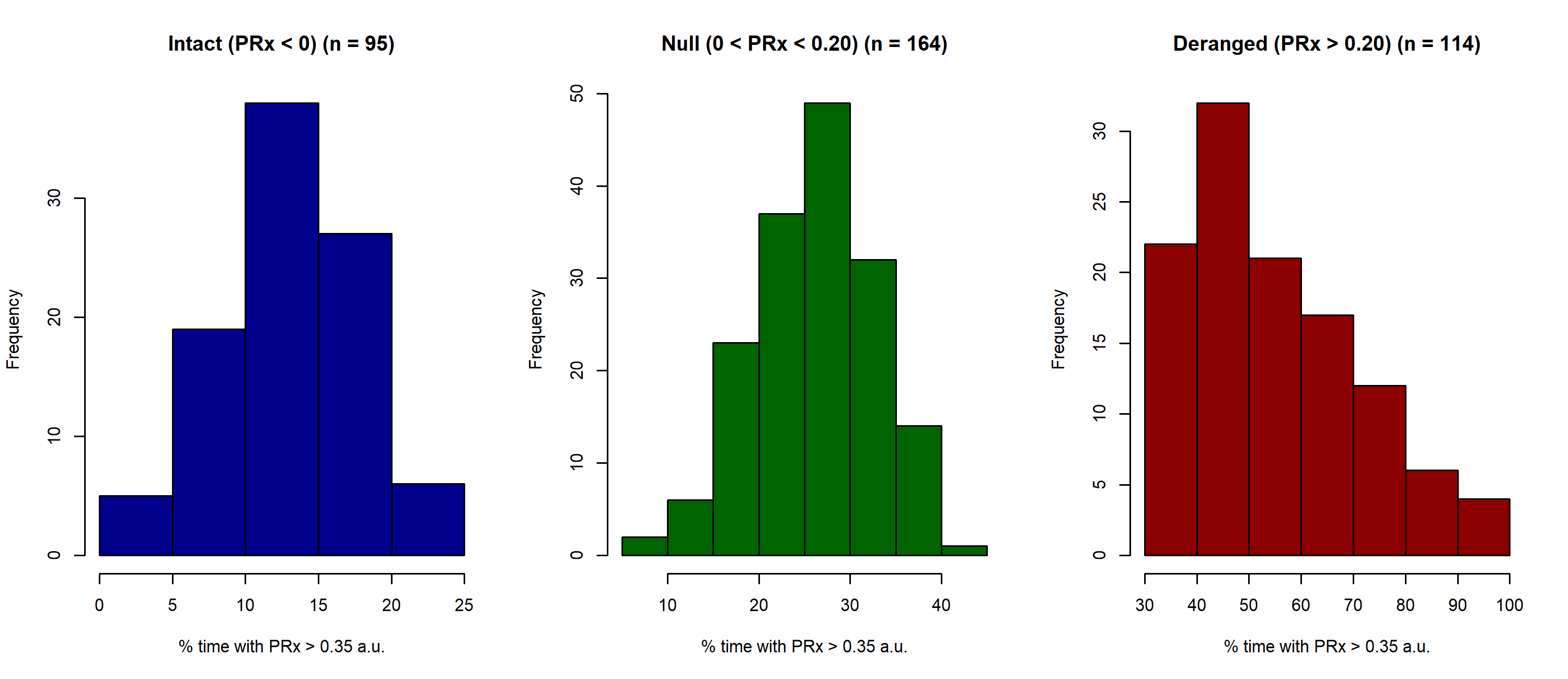

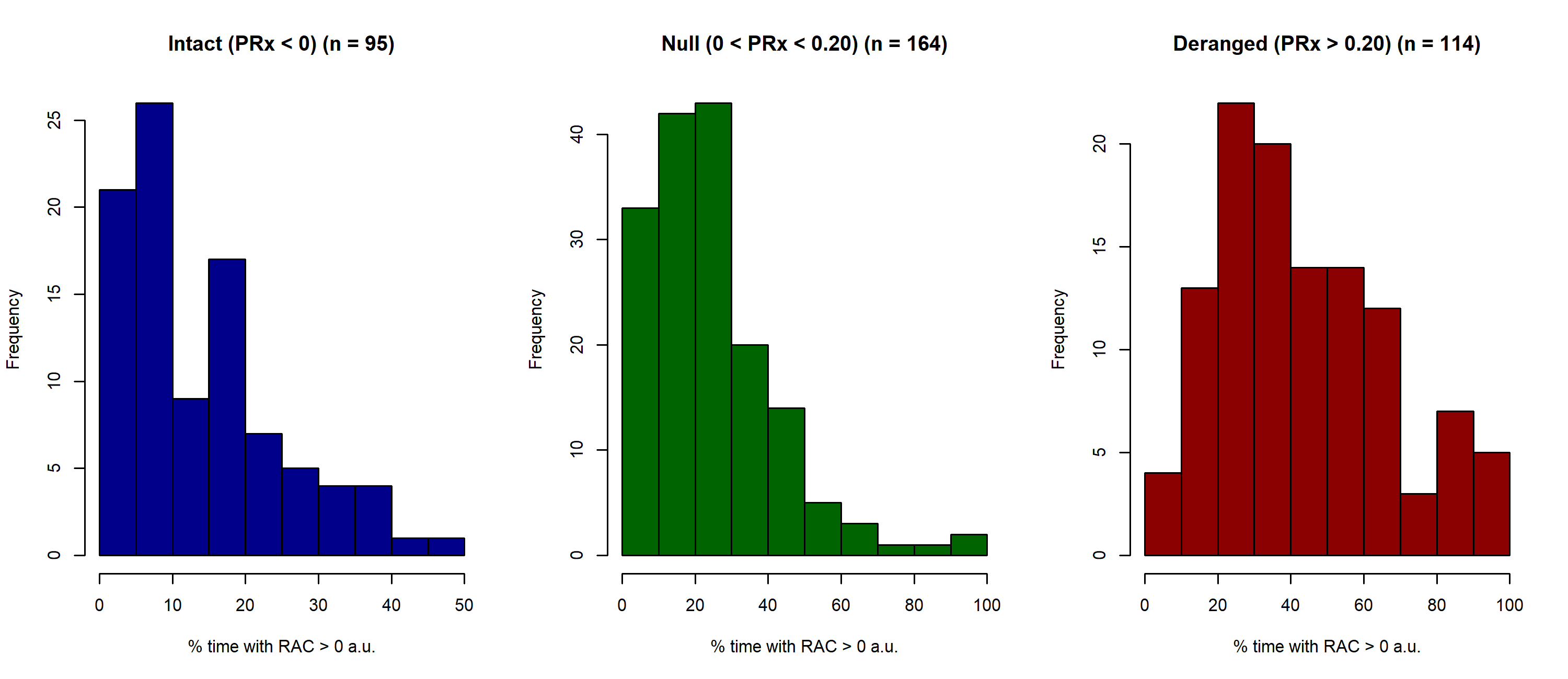

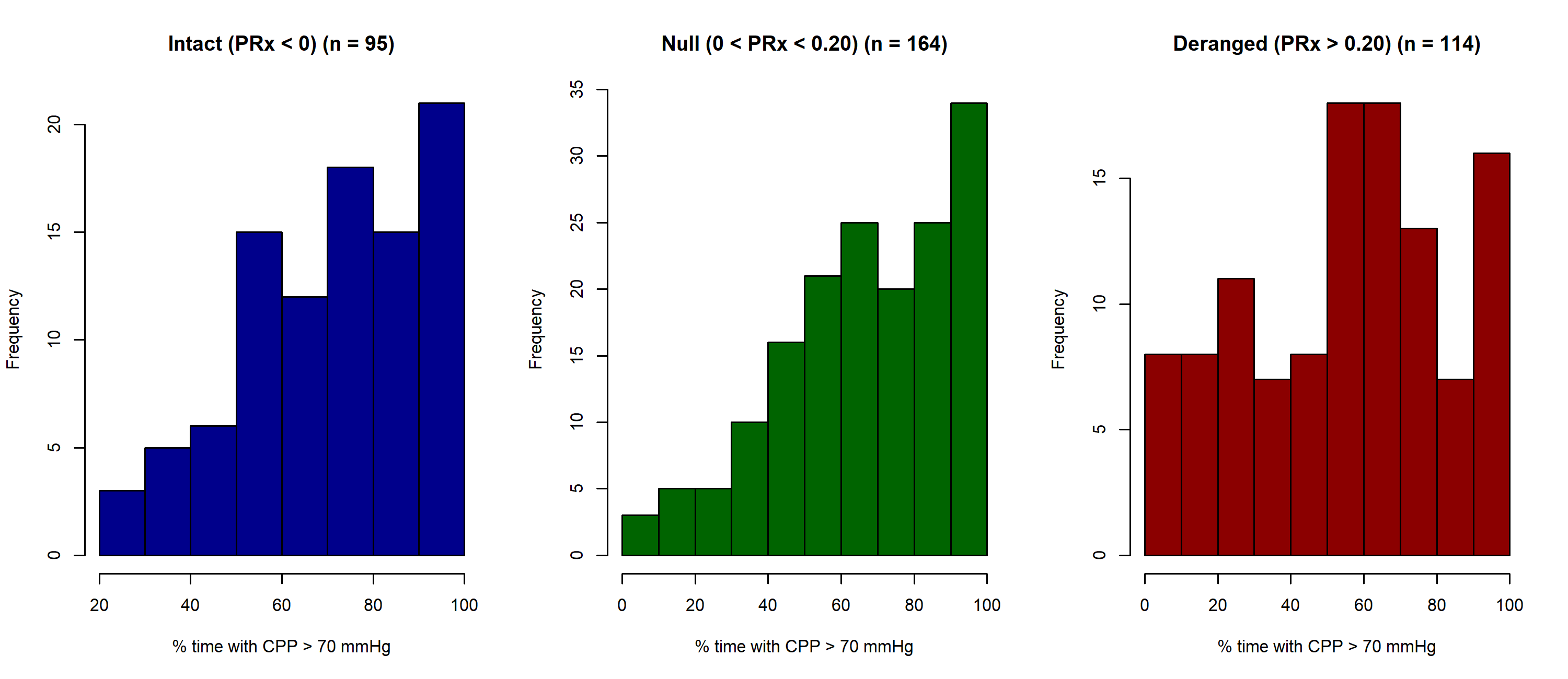

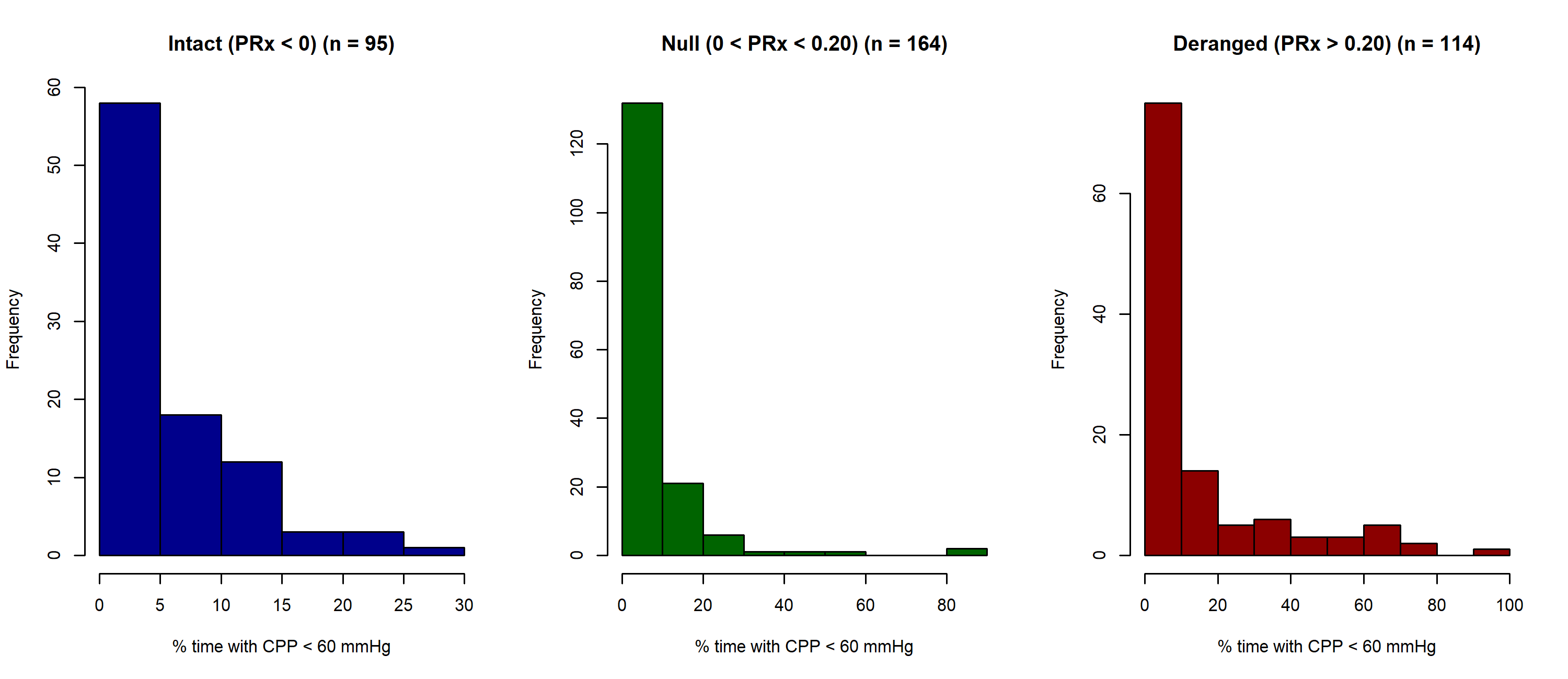

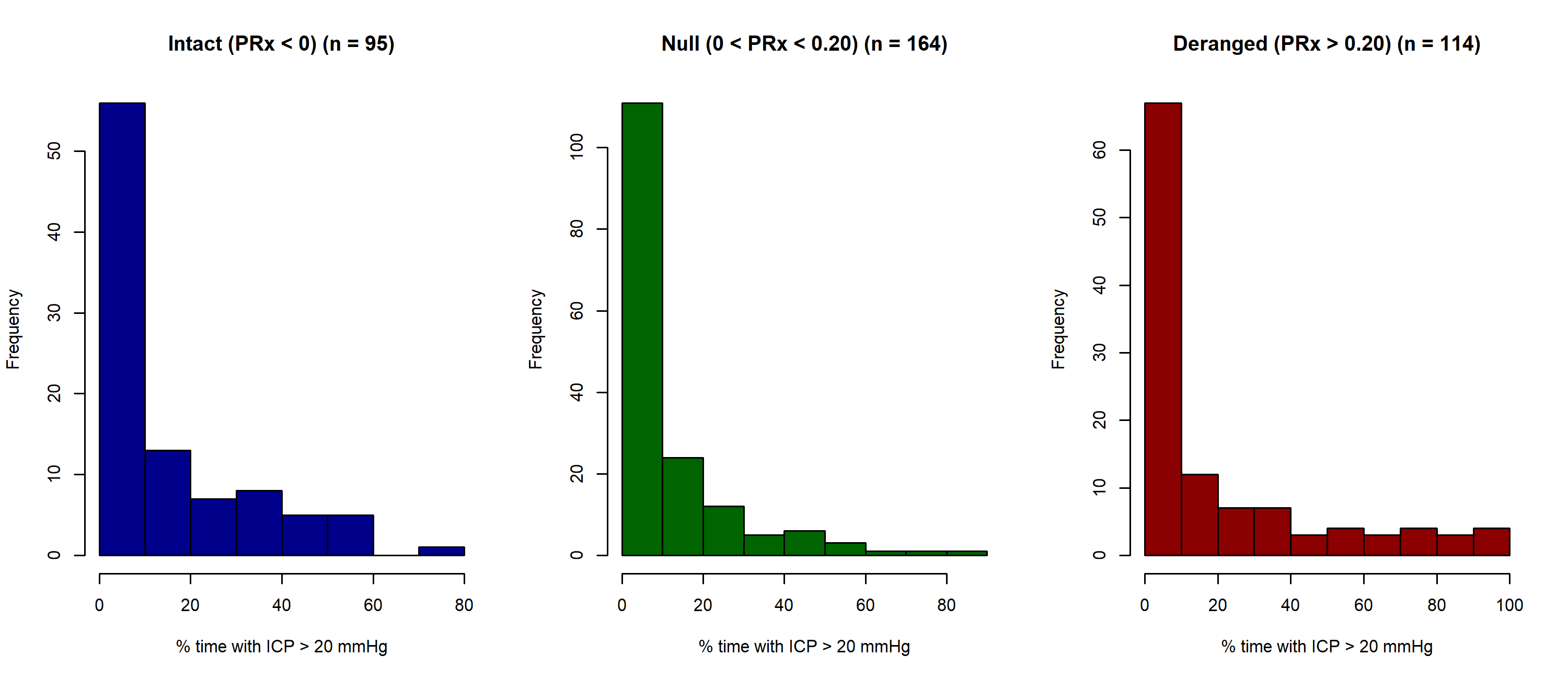


*AMP = pulse amplitude of ICP, COx = cerebral oxygenation index (correlation between rSO2 and CPP), CPP = cerebral perfusion pressure, ICP = intracranial pressure, MAP = mean arterial pressure, PAx = pulse amplitude index (correlation between AMP and MAP), PbtO2 = brain tissue oxygen tension, PRx = pressure reactivity index (correlation between ICP and MAP), RAC = correlation (R) between slow waves of AMP (A) and CPP (C), RAP = compensatory reserve index (correlation between AMP and ICP), rSO2 = regional cerebral oxygen saturation.*

Supplemental Appendix M. Insult Burden Histograms for PbtO_2_ Trichotomization


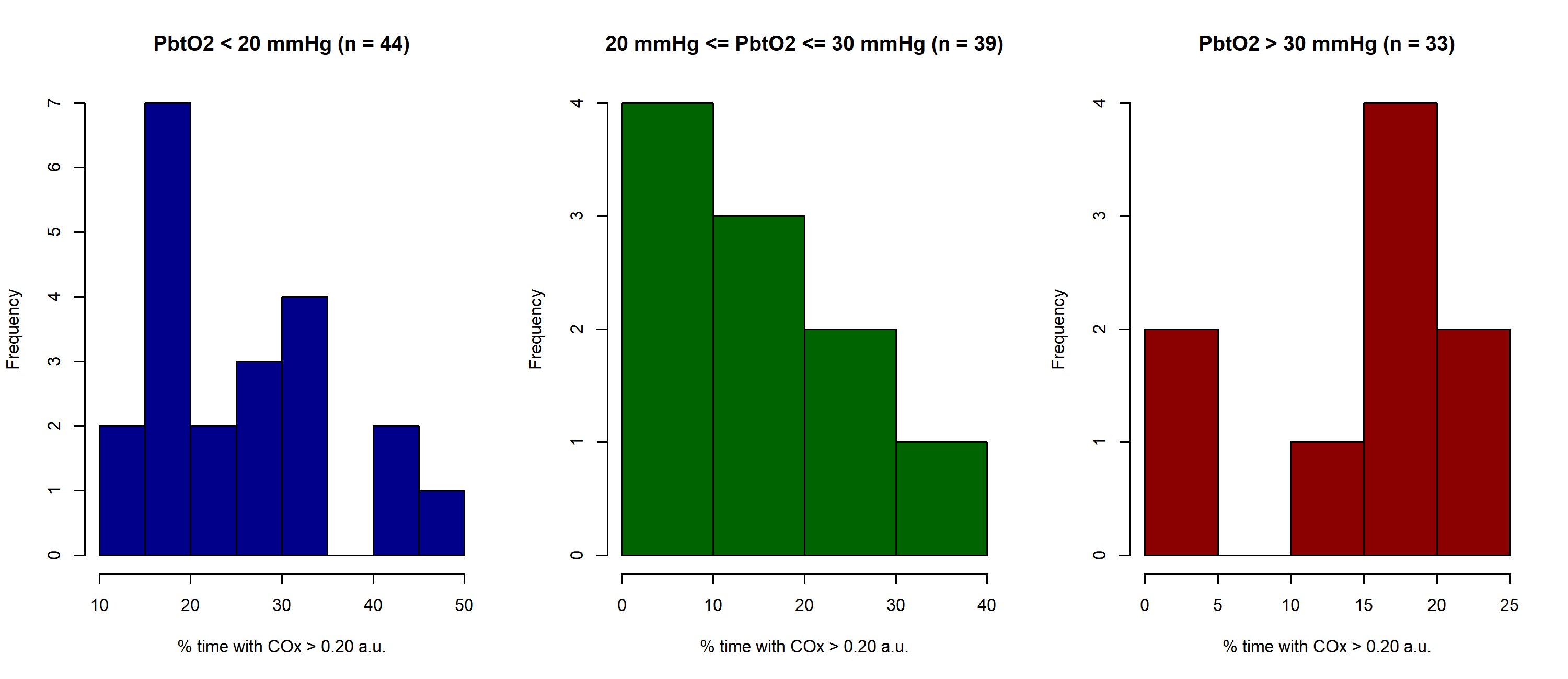

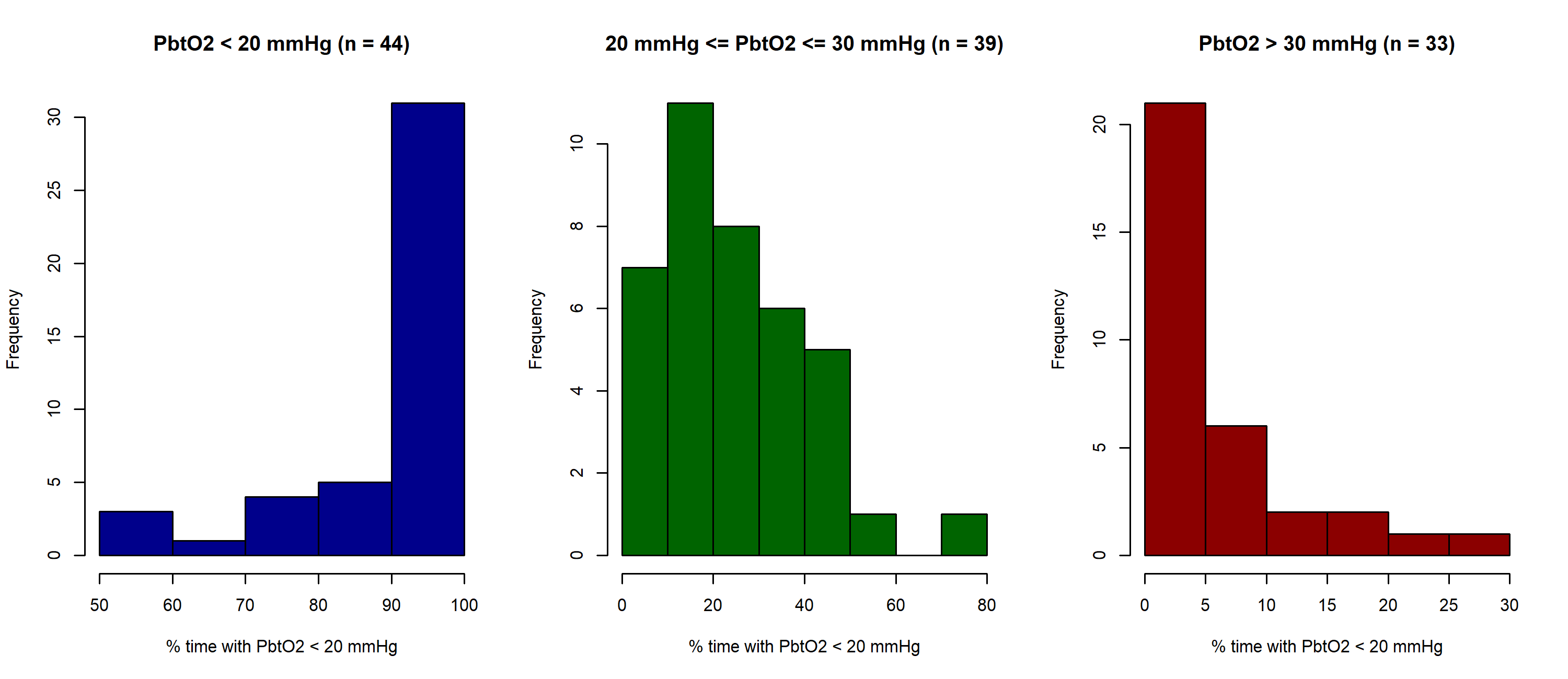

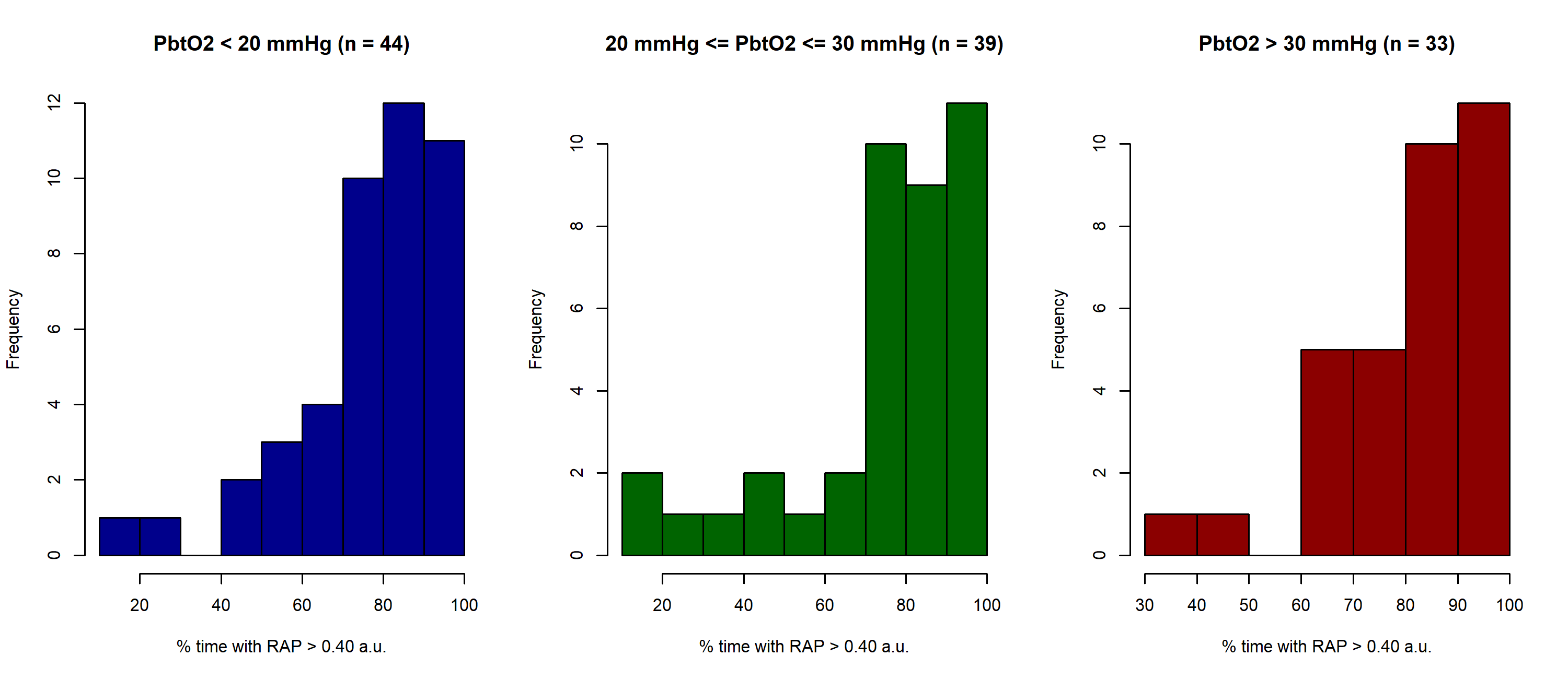

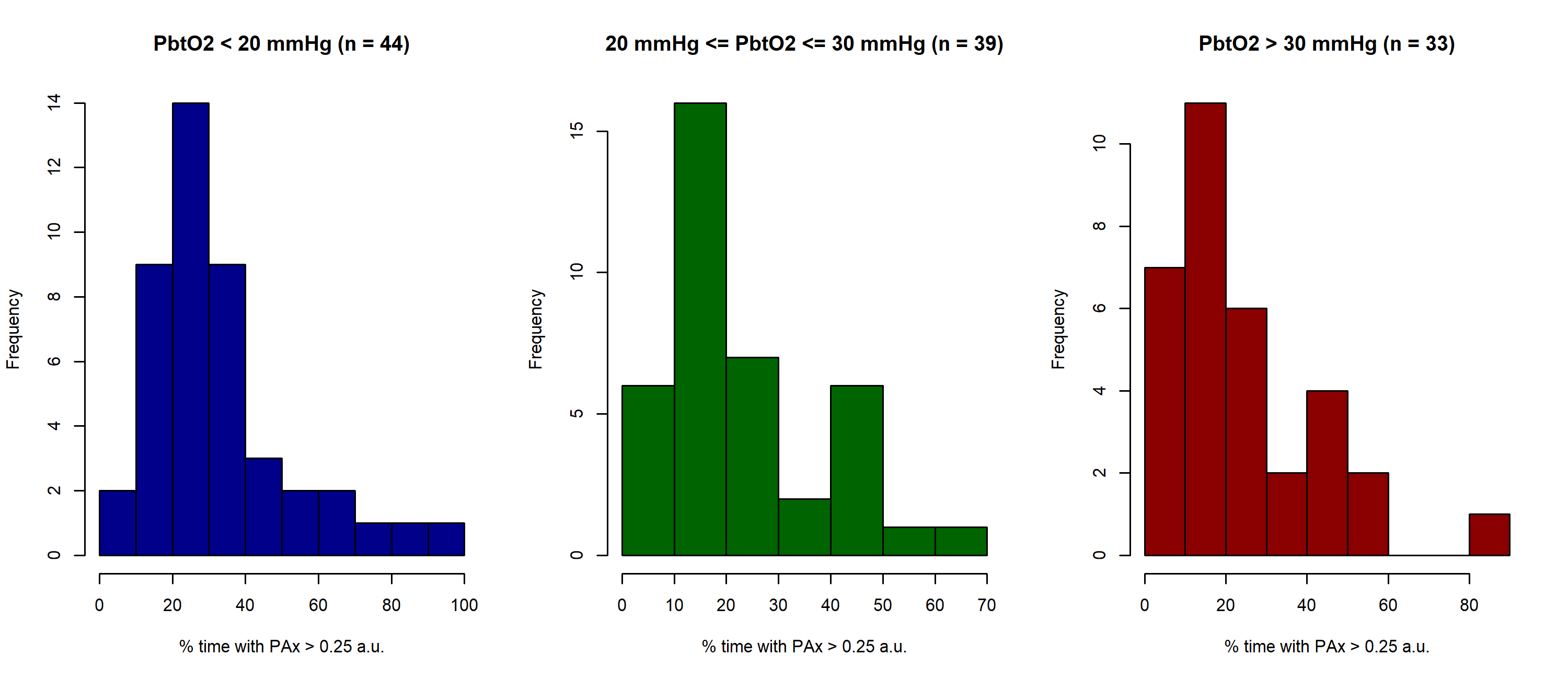

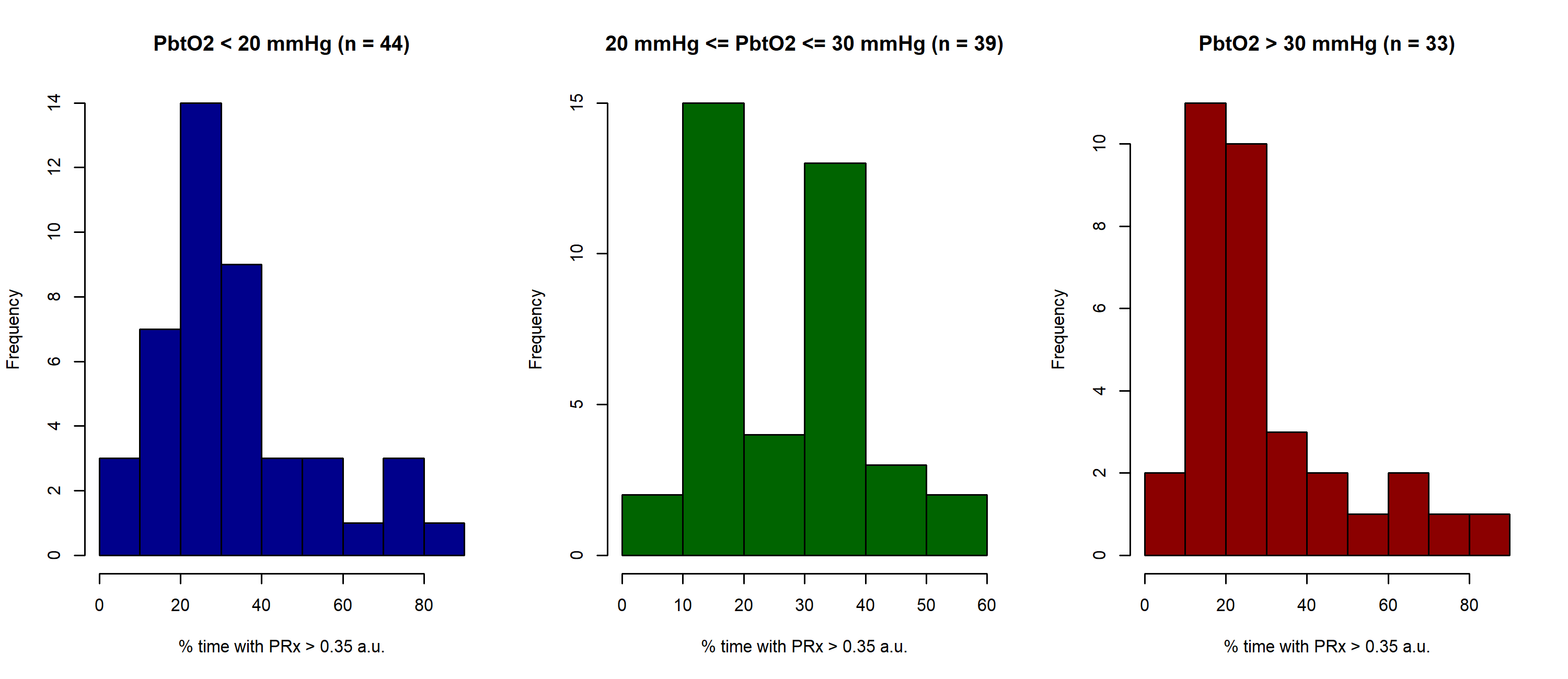

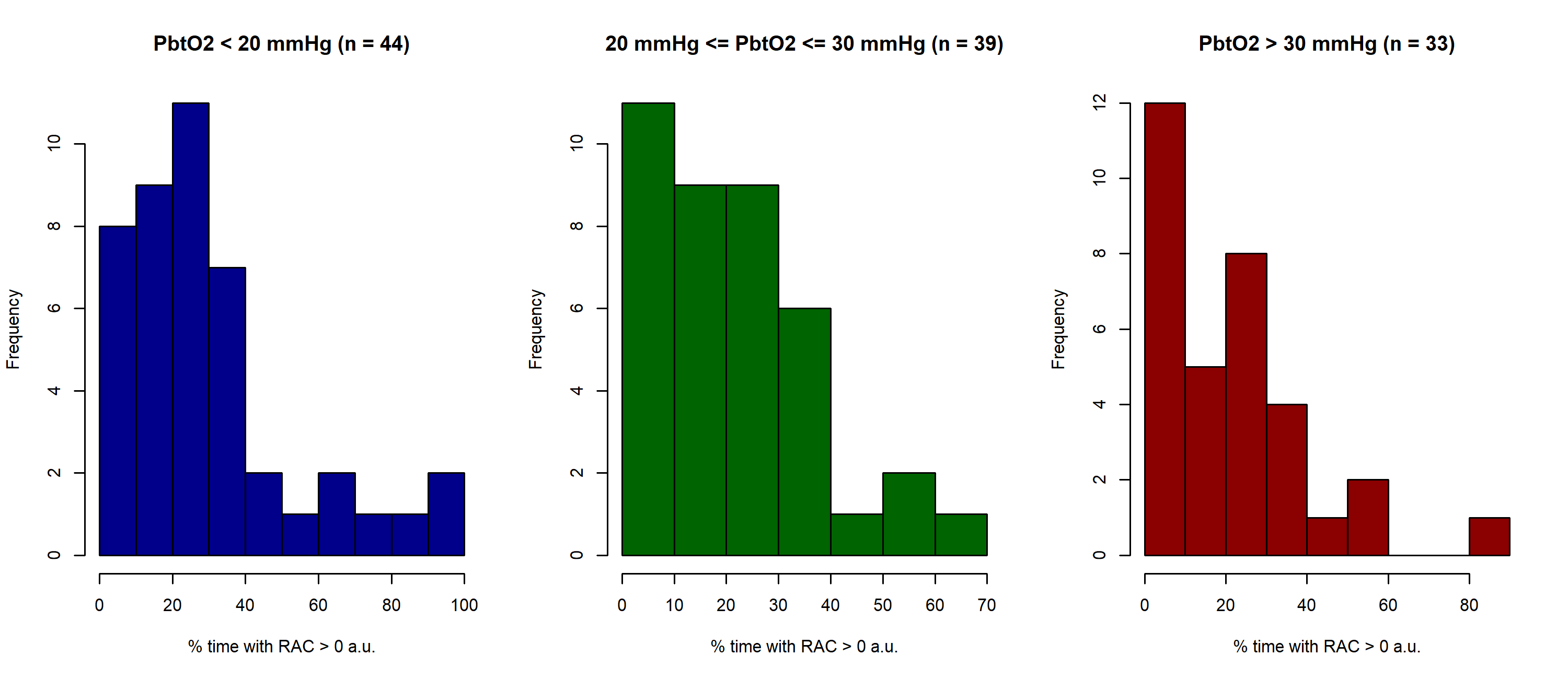

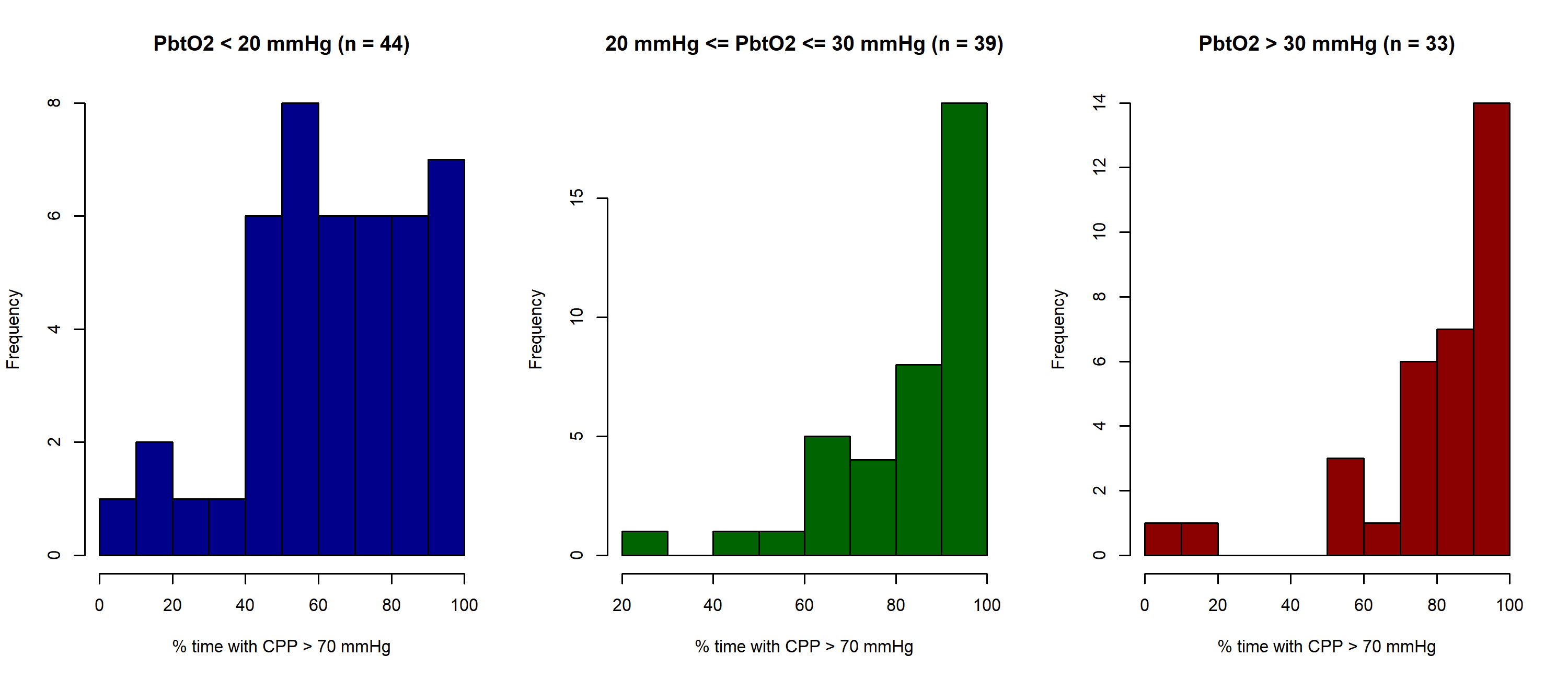

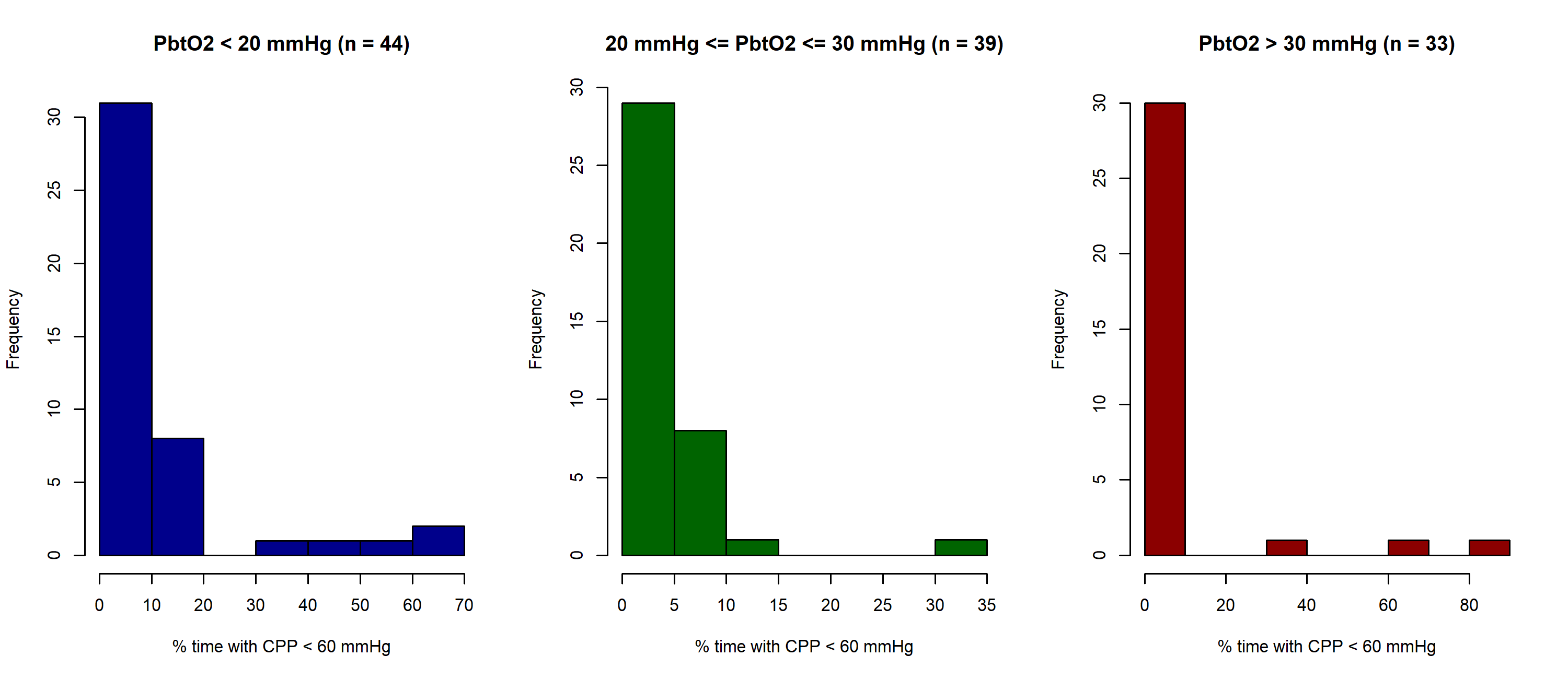

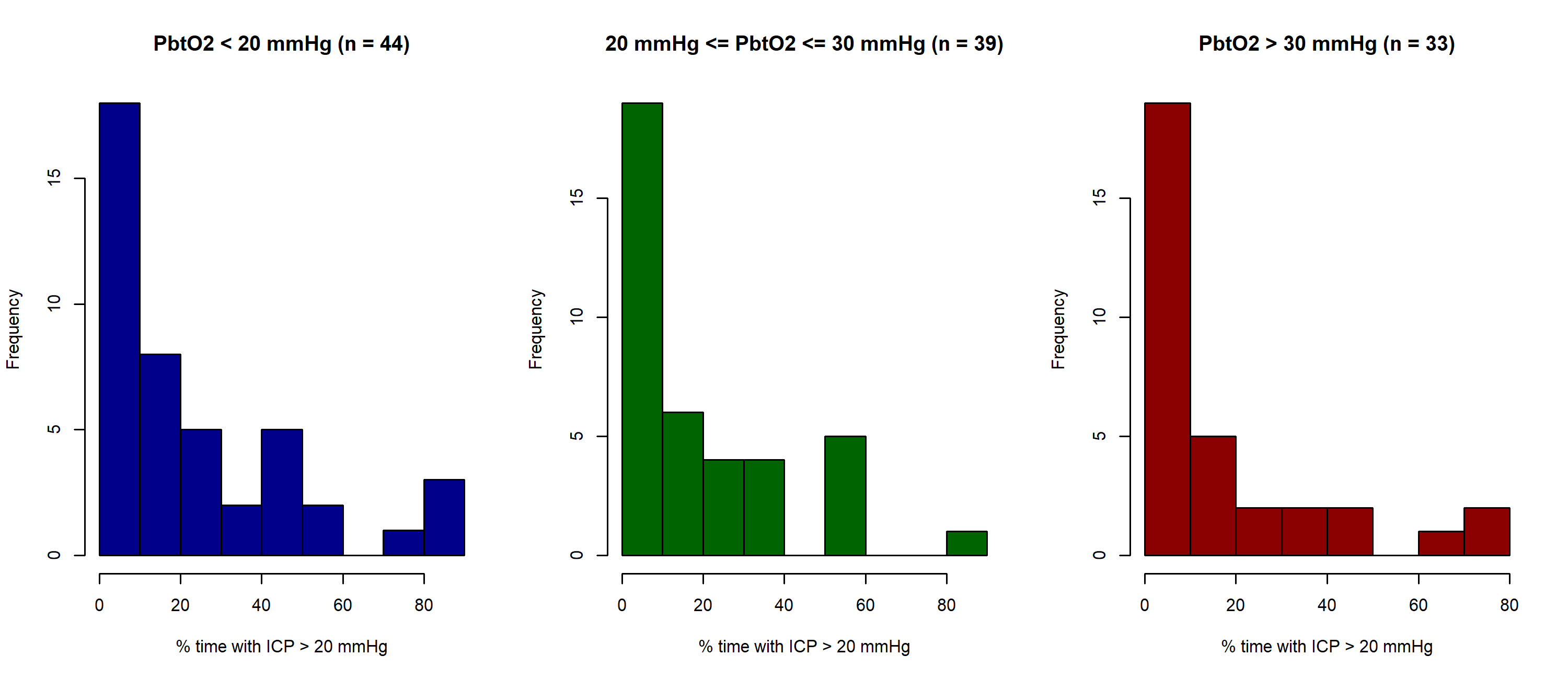


*AMP = pulse amplitude of ICP, COx = cerebral oxygenation index (correlation between rSO2 and CPP), CPP = cerebral perfusion pressure, ICP = intracranial pressure, MAP = mean arterial pressure, PAx = pulse amplitude index (correlation between AMP and MAP), PbtO2 = brain tissue oxygen tension, PRx = pressure reactivity index (correlation between ICP and MAP), RAC = correlation (R) between slow waves of AMP (A) and CPP (C), RAP = compensatory reserve index (correlation between AMP and ICP), rSO2 = regional cerebral oxygen saturation.*

Supplemental Appendix N. Insult Burden Histograms for rSO_2_ Trichotomization


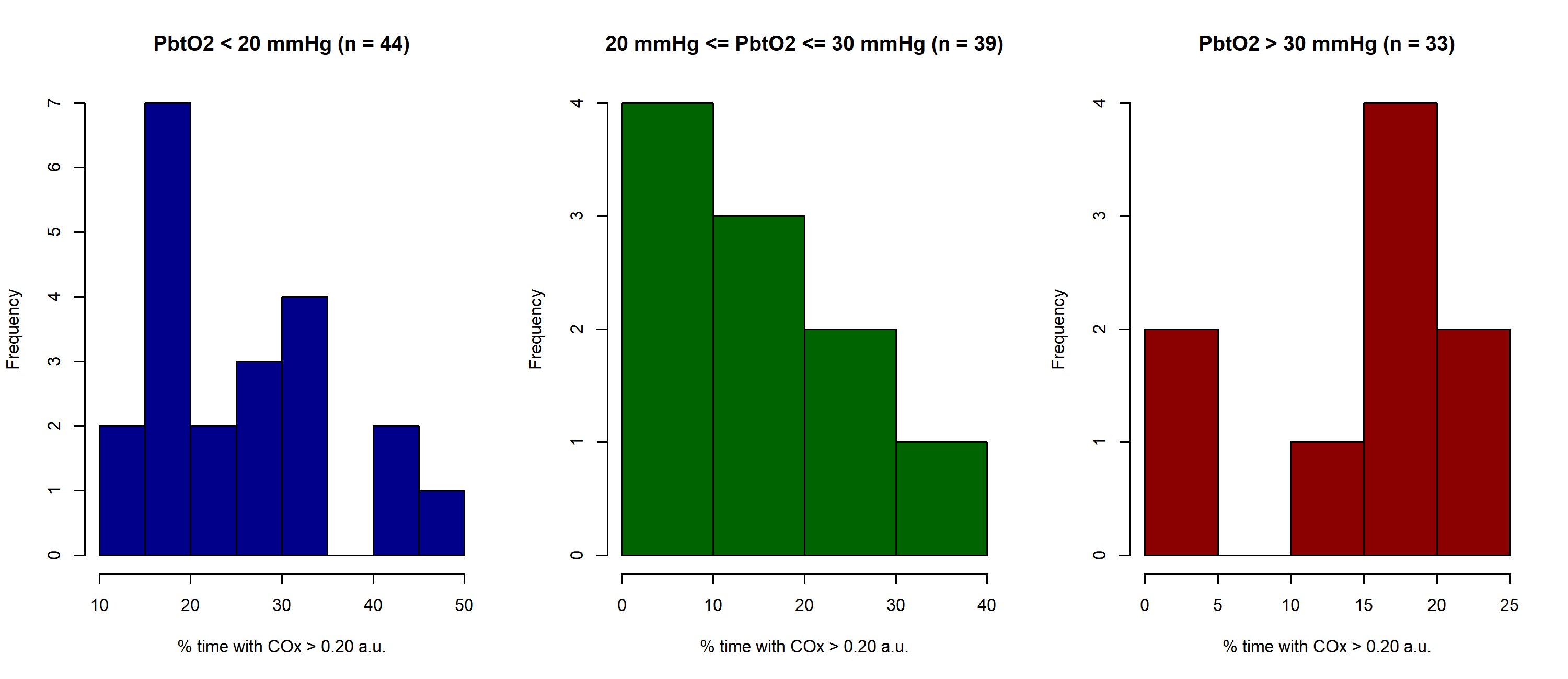

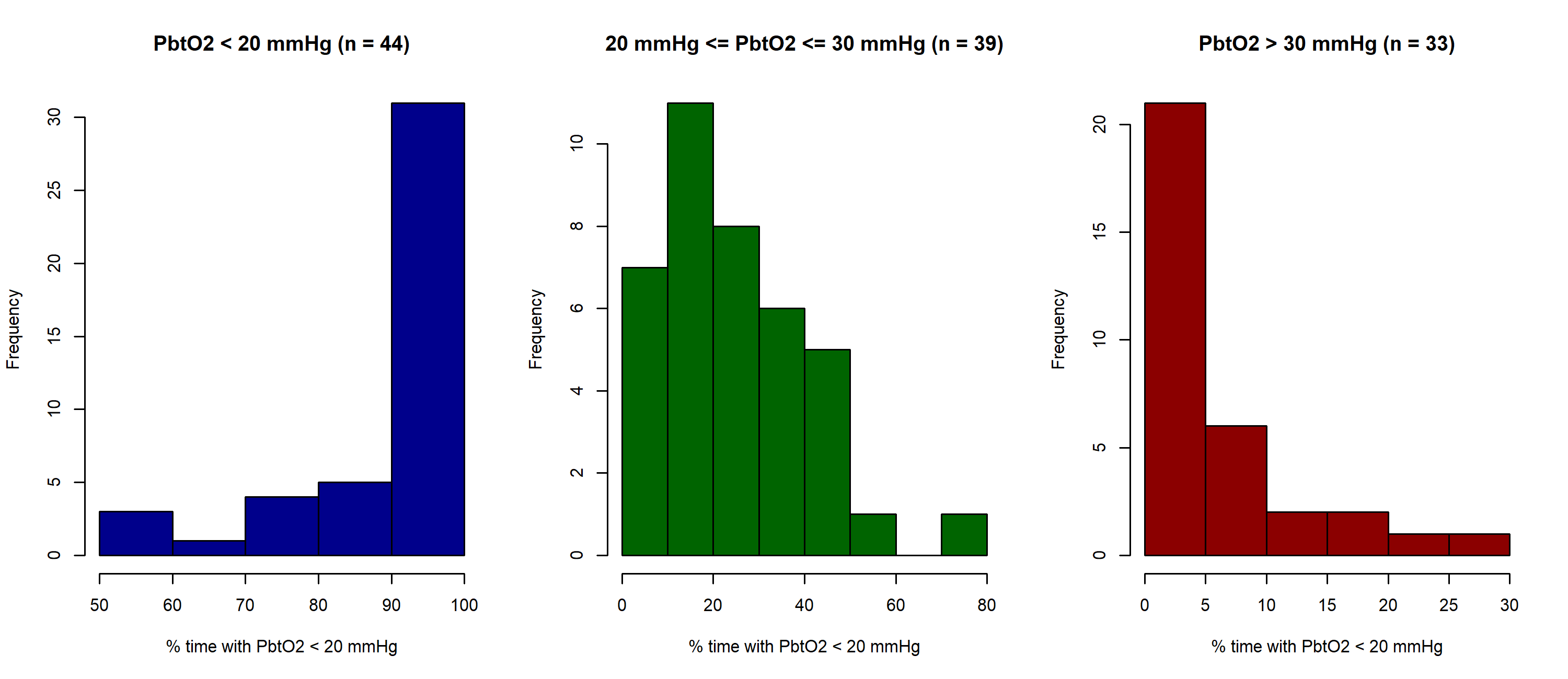

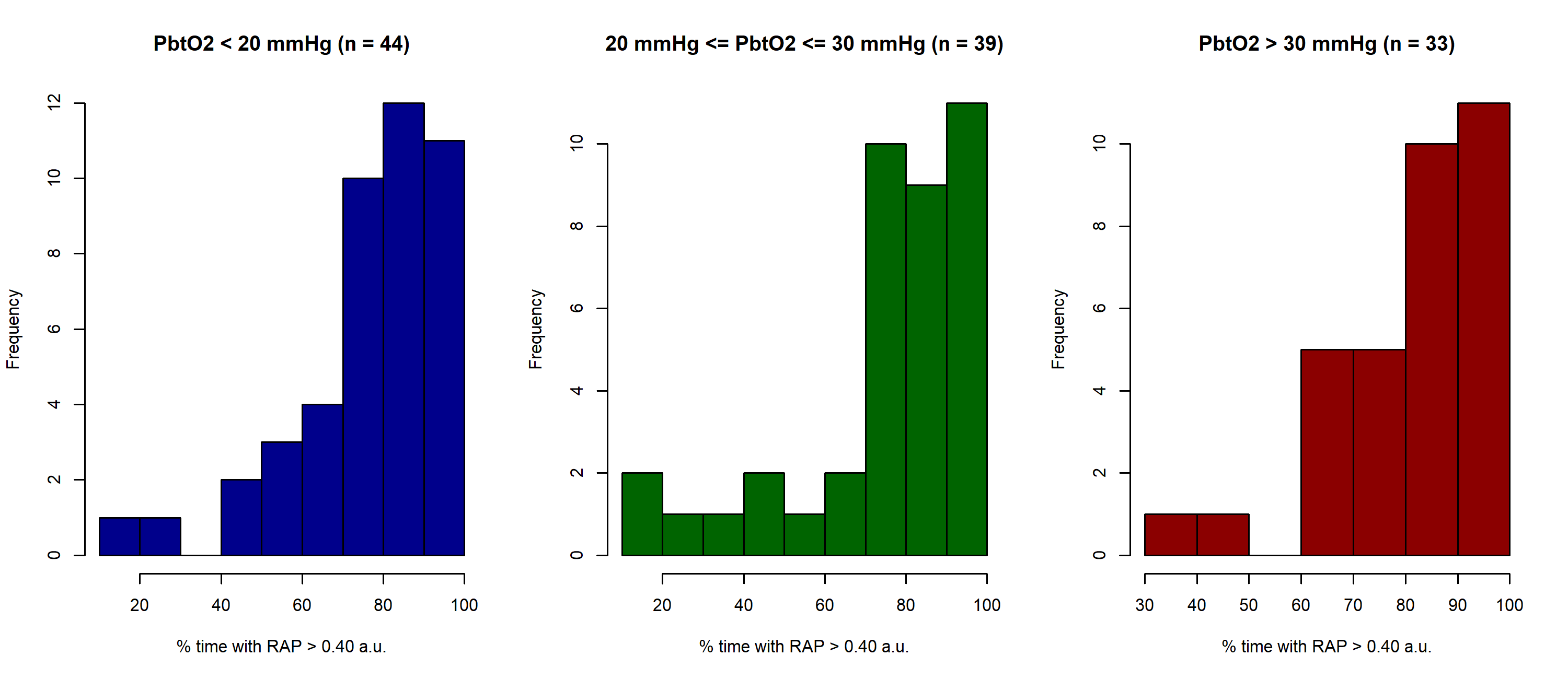

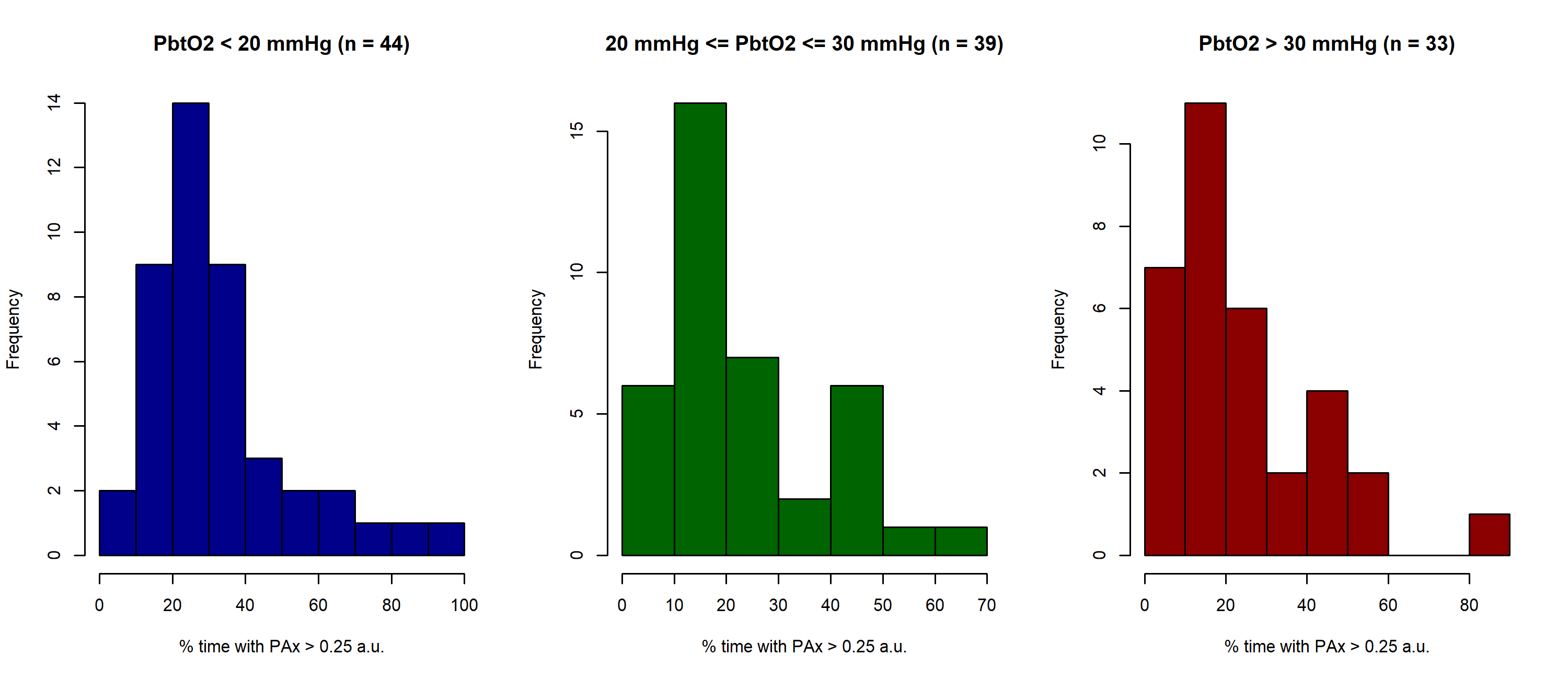

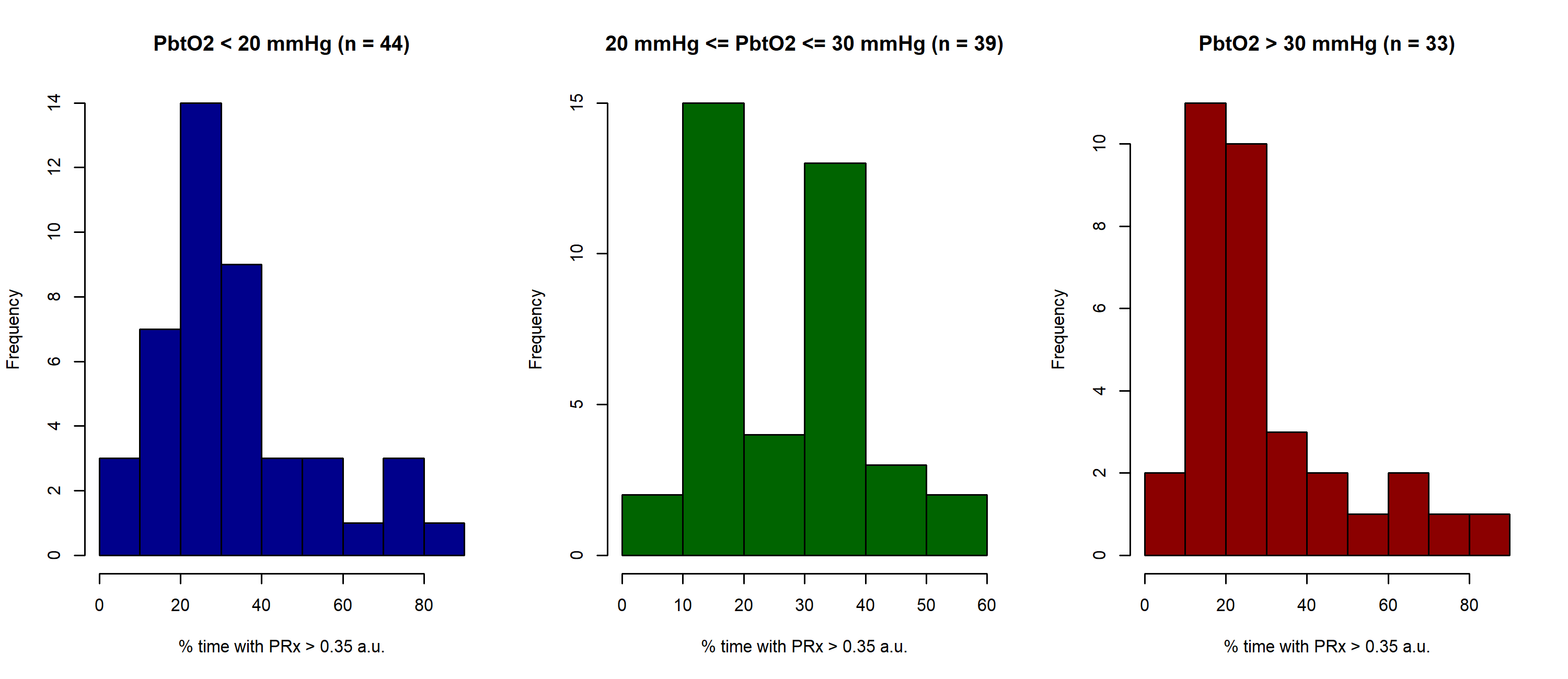

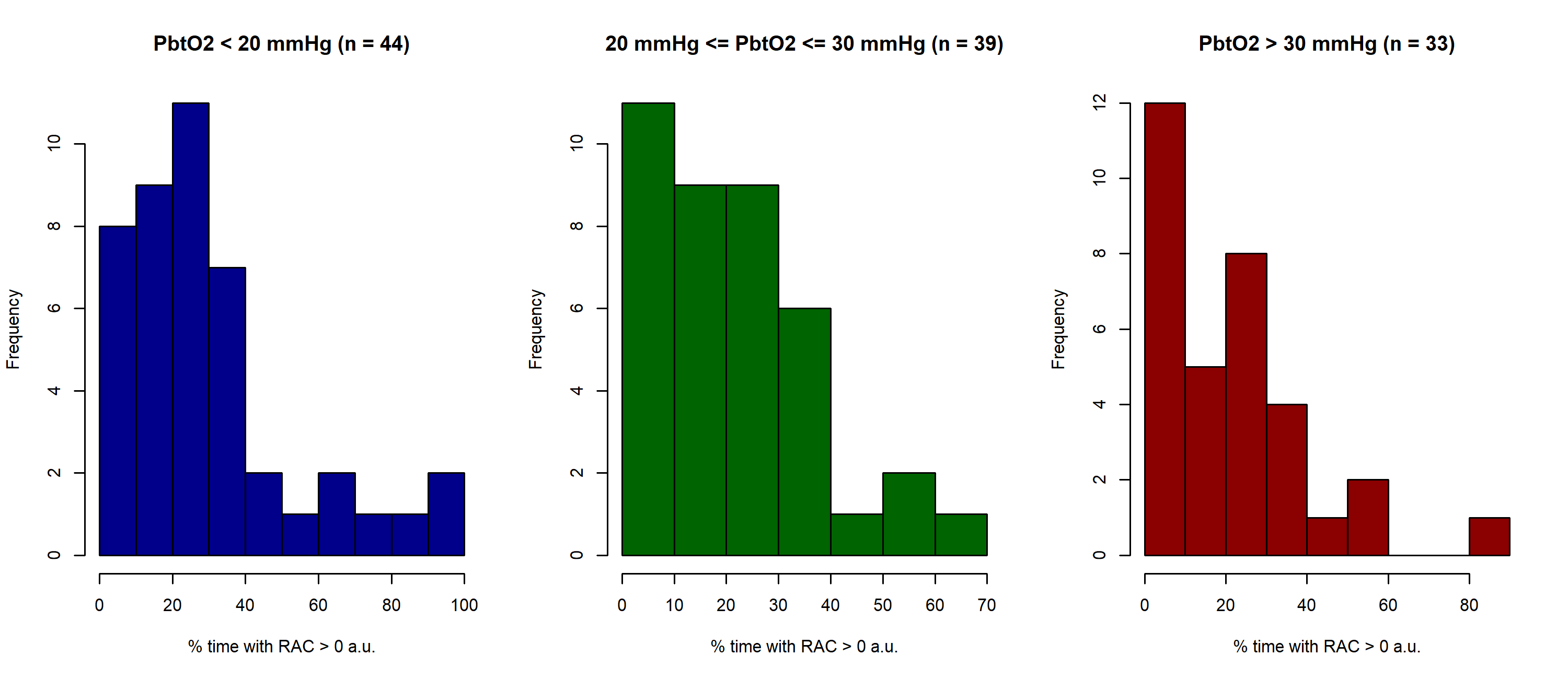

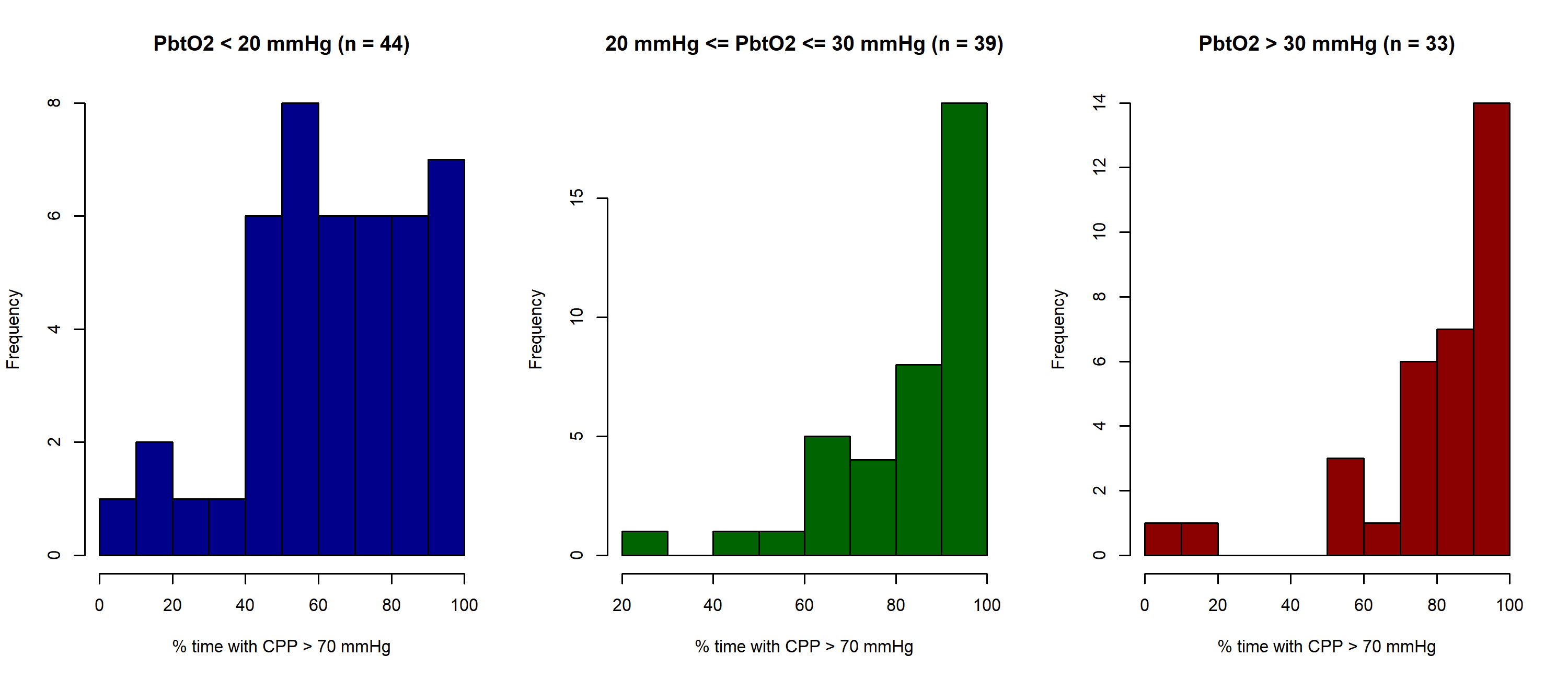

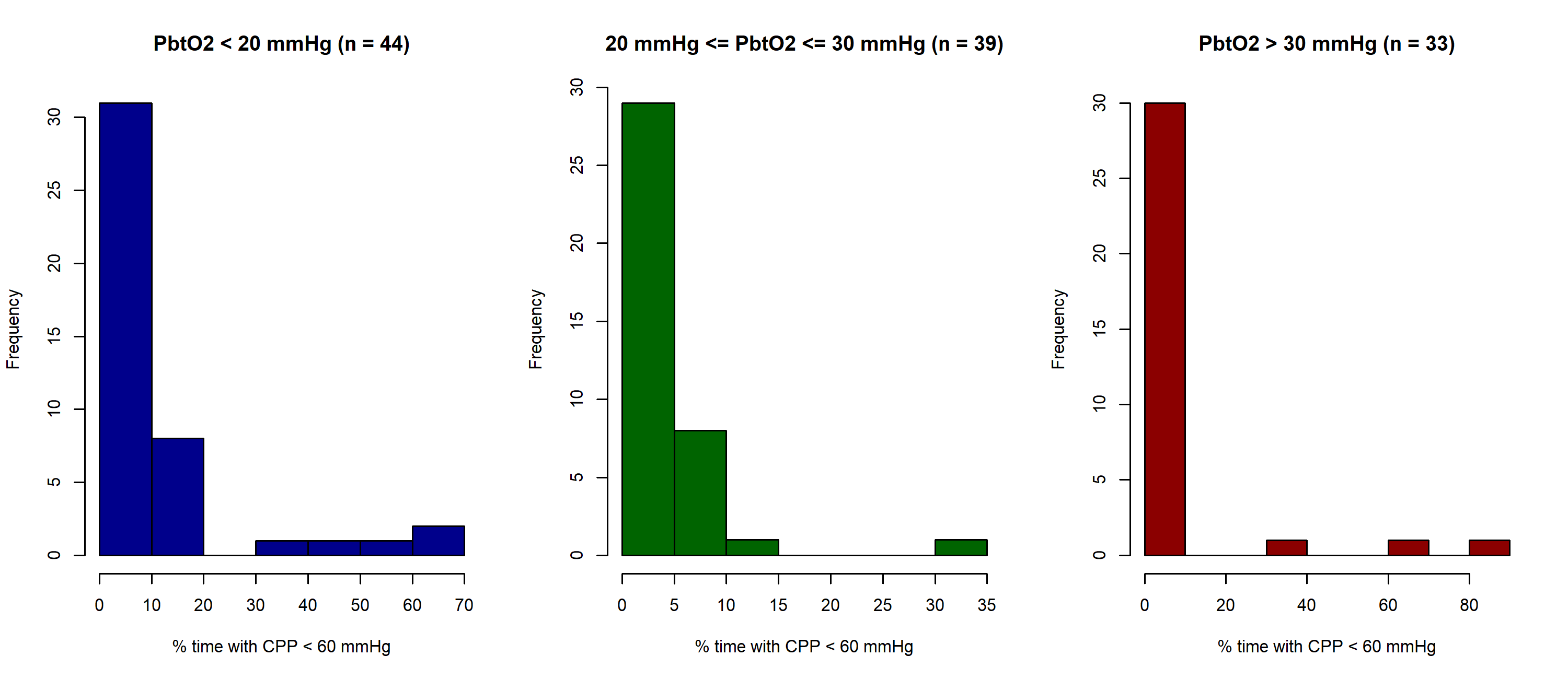

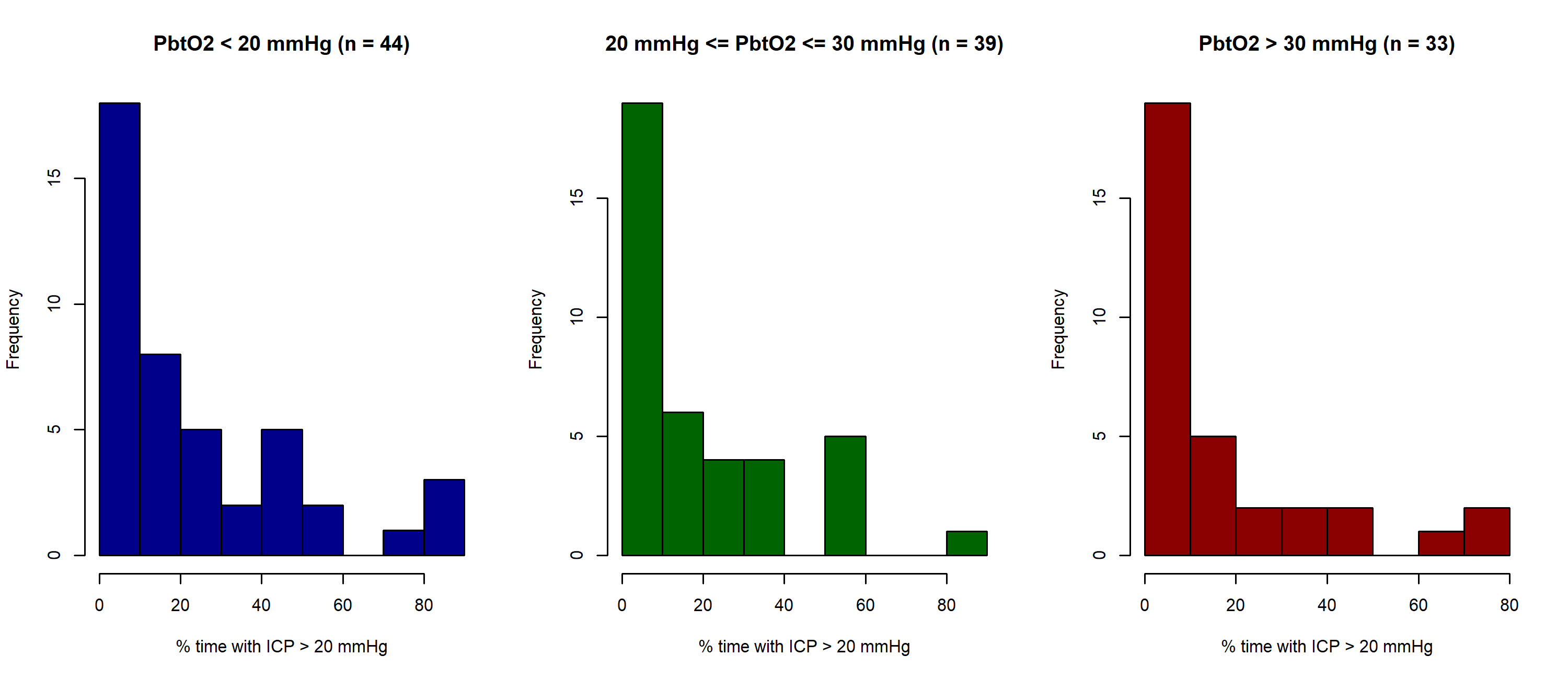


*AMP = pulse amplitude of ICP, COx = cerebral oxygenation index (correlation between rSO2 and CPP), CPP = cerebral perfusion pressure, ICP = intracranial pressure, MAP = mean arterial pressure, PAx = pulse amplitude index (correlation between AMP and MAP), PbtO2 = brain tissue oxygen tension, PRx = pressure reactivity index (correlation between ICP and MAP), RAC = correlation (R) between slow waves of AMP (A) and CPP (C), RAP = compensatory reserve index (correlation between AMP and ICP), rSO2 = regional cerebral oxygen saturation.*

Supplemental Appendix O. Contour Plots Demonstrating the Effects of Cerebrovascular Reactivity on the Relationship Between ICP and Global Cerebral Insult Burden.


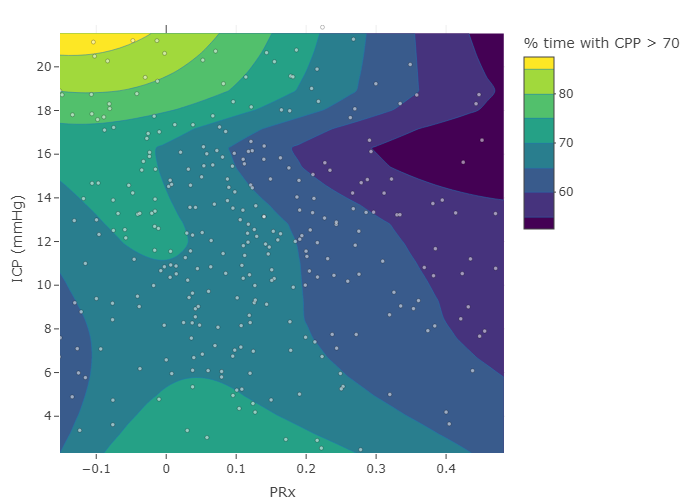

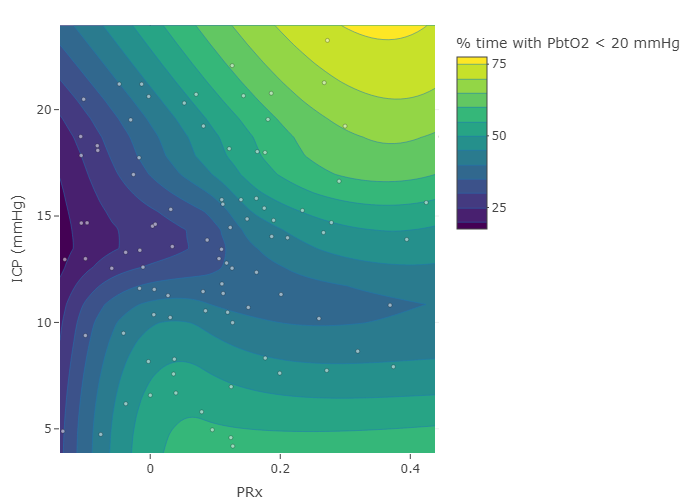

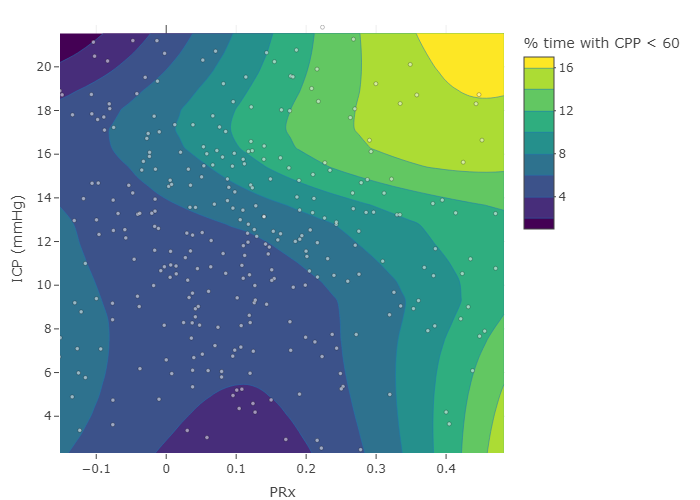

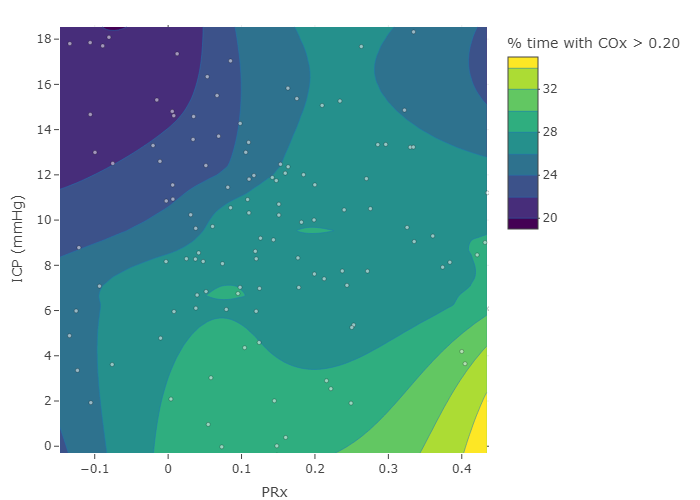

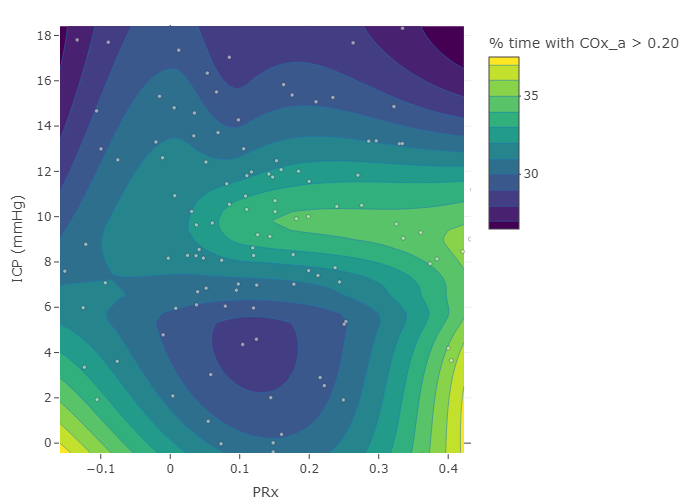


Data points used to construct the contour plots are overlayed on each plot. *ABP = arterial blood pressure, COx = cerebral oxygenation index (correlation between rSO2 and CPP), COx_a = cerebral oxygenation index (correlation between rSO2 and ABP),CPP = cerebral perfusion pressure, ICP = intracranial pressure, MAP = mean arterial pressure, PbtO2 = brain tissue oxygen tension, PRx = pressure reactivity index (correlation between ICP and MAP), rSO2 = regional cerebral oxygen saturation.*

Supplemental Appendix P. Contour Plots Demonstrating the Effects of Cerebrovascular Reactivity on the Relationship Between CPP and Global Cerebral Insult Burden.


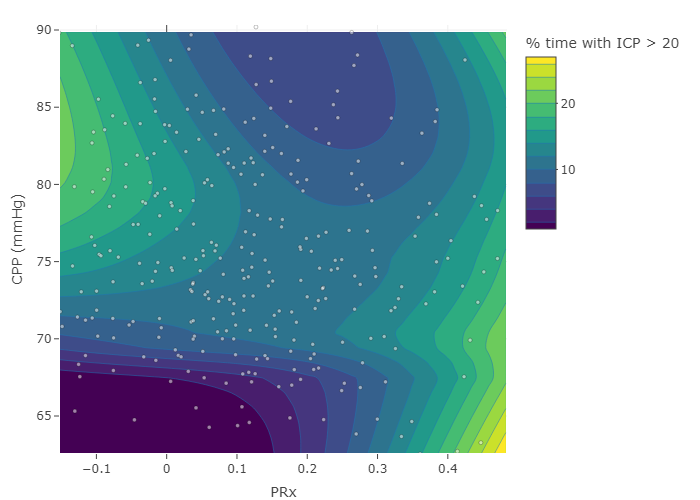

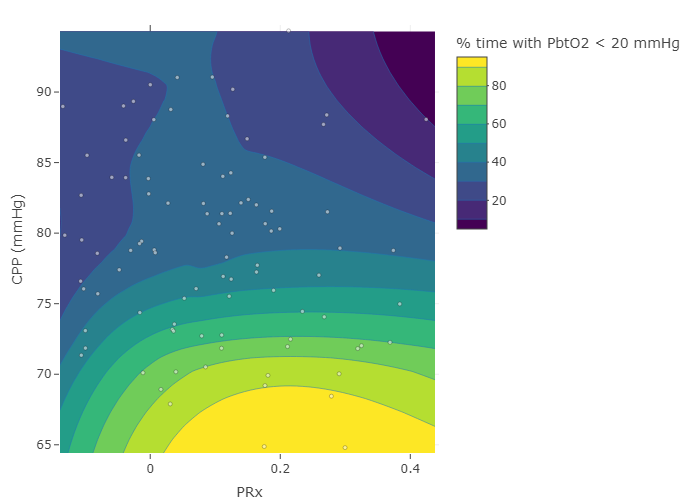

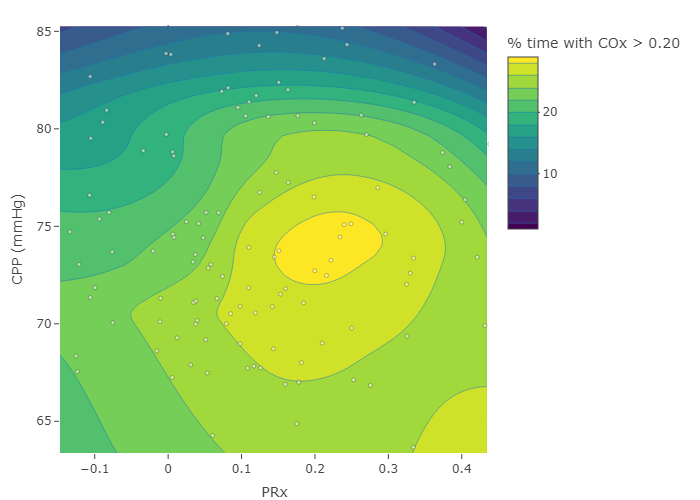

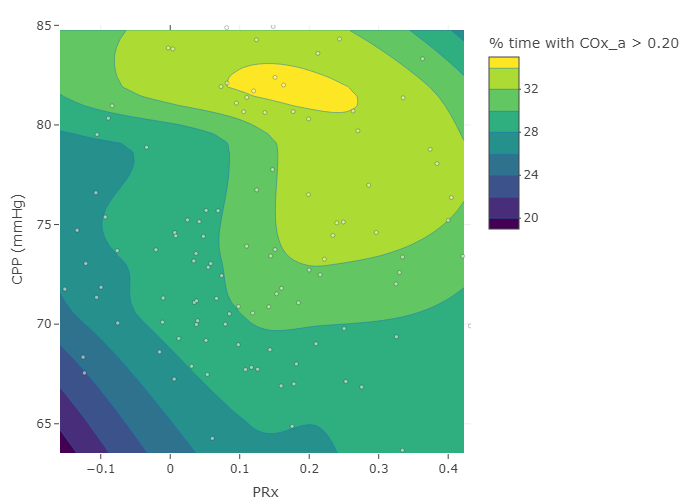


Data points used to construct the contour plots are overlayed on each plot. *ABP = arterial blood pressure, COx = cerebral oxygenation index (correlation between rSO2 and CPP), COx_a = cerebral oxygenation index (correlation between rSO2 and ABP),CPP = cerebral perfusion pressure, ICP = intracranial pressure, MAP = mean arterial pressure, PbtO2 = brain tissue oxygen tension, PRx = pressure reactivity index (correlation between ICP and MAP), rSO2 = regional cerebral oxygen saturation.*

Supplemental Appendix Q. Contour Plots Demonstrating the Effects of Cerebrovascular Reactivity on the Relationship Between PbtO_2_ and Global Cerebral Insult Burden.


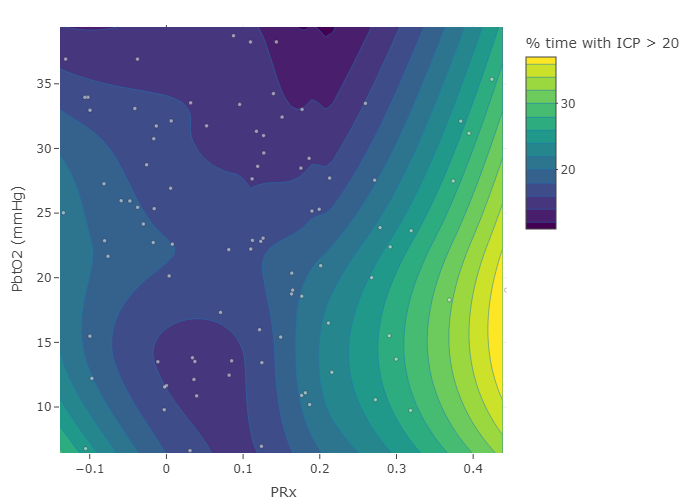

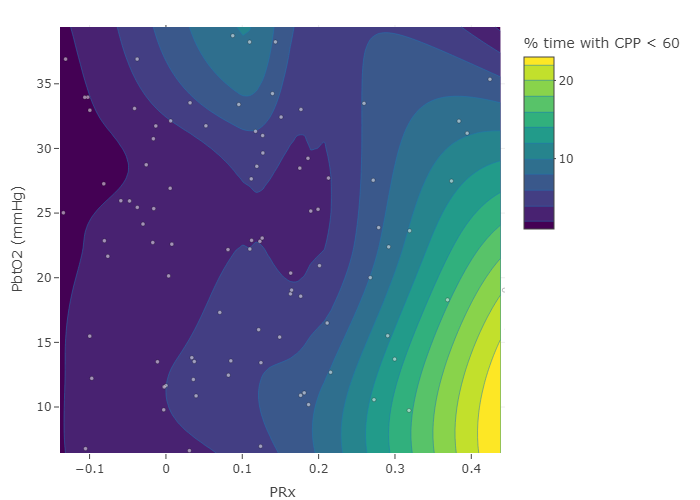

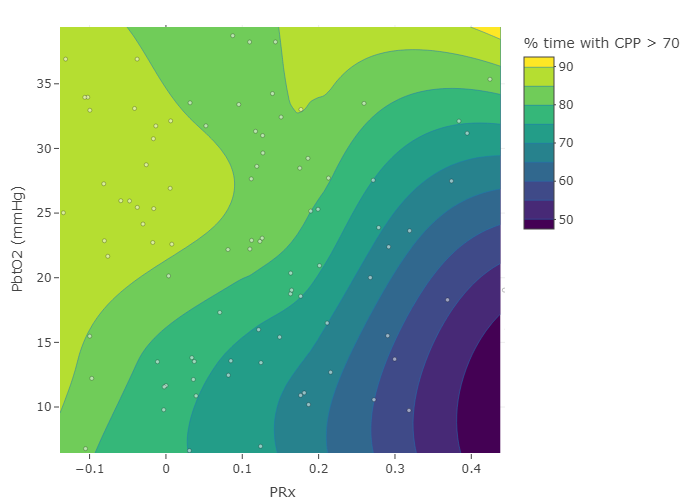

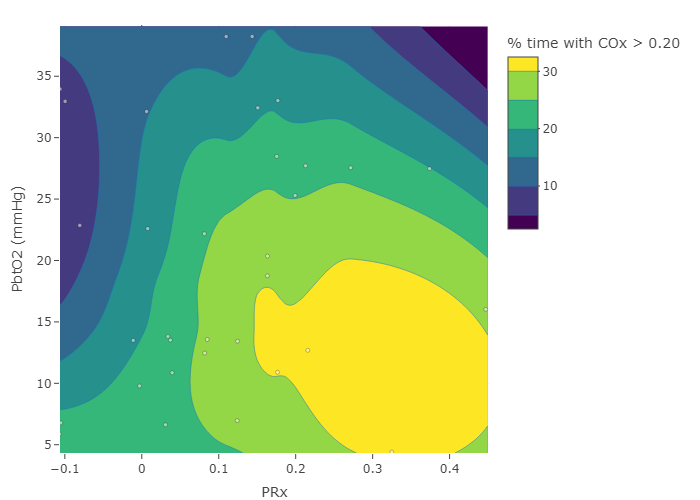

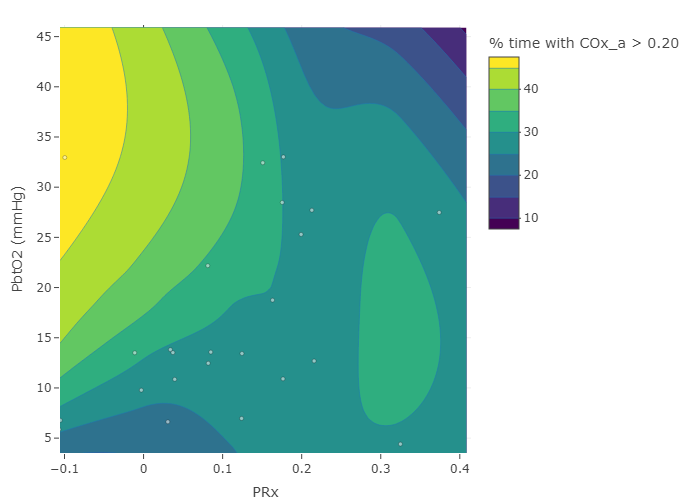


Data points used to construct the contour plots are overlayed on each plot. *ABP = arterial blood pressure, COx = cerebral oxygenation index (correlation between rSO2 and CPP), COx_a = cerebral oxygenation index (correlation between rSO2 and ABP),CPP = cerebral perfusion pressure, ICP = intracranial pressure, MAP = mean arterial pressure, PbtO2 = brain tissue oxygen tension, PRx = pressure reactivity index (correlation between ICP and MAP), rSO2 = regional cerebral oxygen saturation.*

Supplemental Appendix R. Contour Plots Demonstrating the Effects of Cerebrovascular Reactivity on the Relationship Between rSO_2_ and Global Cerebral Insult Burden.


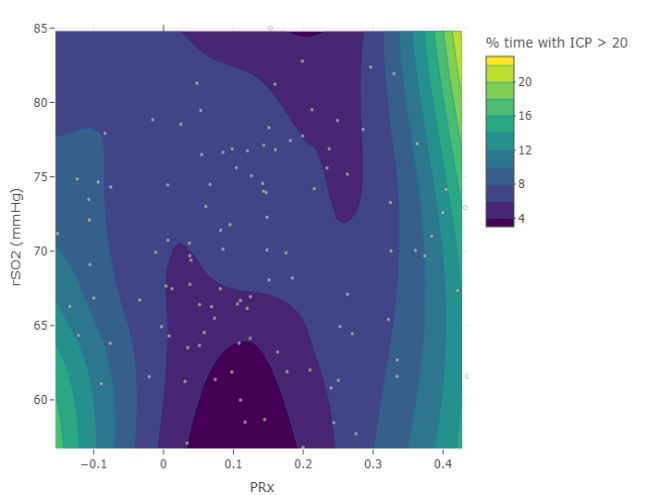

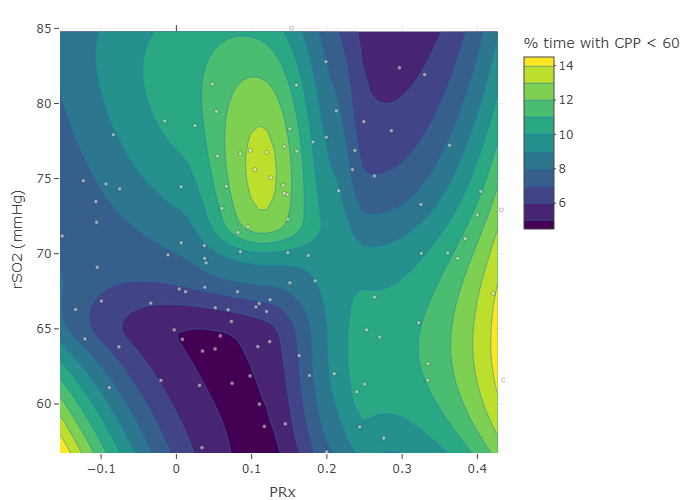

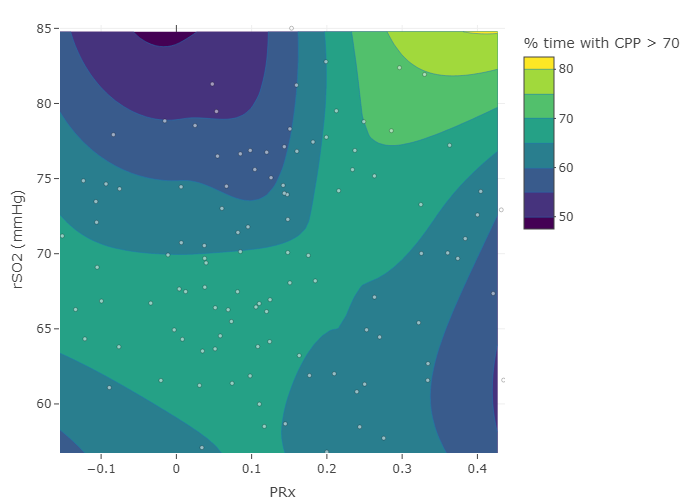

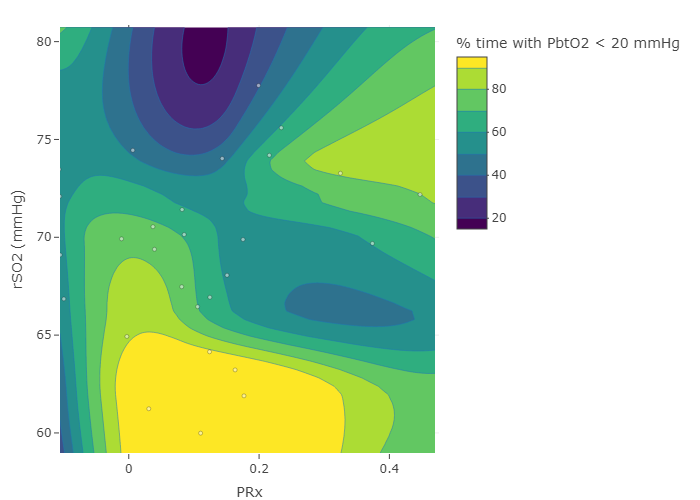


Data points used to construct the contour plots are overlayed on each plot. *ABP = arterial blood pressure, COx = cerebral oxygenation index (correlation between rSO2 and CPP), COx_a = cerebral oxygenation index (correlation between rSO2 and ABP),CPP = cerebral perfusion pressure, ICP = intracranial pressure, MAP = mean arterial pressure, PbtO2 = brain tissue oxygen tension, PRx = pressure reactivity index (correlation between ICP and MAP), rSO2 = regional cerebral oxygen saturation.*
